# Biosynthesis of the Fully Saturated C_16_ Homosesquiterpene Descartane Through Deprotonation With Cyclopropanation

**DOI:** 10.1002/anie.1585472

**Published:** 2026-07-16

**Authors:** Kexin Yang, Philip Troycke, Bernd Goldfuss, Michael Groll, Jeroen S. Dickschat

**Affiliations:** ^1^ Kekulé Institute of Organic Chemistry and Biochemistry University of Bonn Bonn Germany; ^2^ Center for Protein Assemblies Department of Chemistry, TU Munich Garching Germany; ^3^ Department of Chemistry Institute of Organic Chemistry University of Cologne Cologne Germany

**Keywords:** biosynthesis, C_16_ sesquiterpenes, enzyme mechanisms, isotopes, methyltransferases

## Abstract

Non‐canonical terpene biosynthesis expands structural diversity beyond the classical C_5n_ logic. Here we report the discovery of descartane, a C_16_ homosesquiterpene with an unprecedented scaffold produced by a bacterial type I terpene cyclase from *Morganella morganii* (MmTC). Unlike most terpene cyclase products, descartane is fully saturated and lacks both alkene and alcohol functions, while containing a cyclopropane ring formed in an unusual termination step. Functional characterisation demonstrated that MmTC converts α‐presodorifen pyrophosphate (α‐PSPP) into descartane. The cyclisation cascade was investigated by extensive isotopic labelling experiments and density functional theory (DFT) calculations, revealing a complex pathway involving fragmentation‐recombination, multiple hydride shifts, and cyclopropane formation. Site‐directed mutagenesis identified active site residues that control product formation and redirect the cascade towards related terpenes, including voltairene, malebranchene, de‐beauvoirol, and pythagorene. These findings establish the biosynthetic origin of descartane and show how subtle changes in the active site can reprogram complex terpene cyclisation cascades.

## Graphical abstract

A non‐canonical C_16_ sesquiterpene synthase from *Morganella morganii* produces the unusual scaffold descartane. Its cyclisation mechanism was rationalised by isotopic labelling experiments and DFT calculations. Site‐directed mutagenesis altered enzyme function, resulting in alternative products, including voltairene, which requires a different mechanistic explanation.

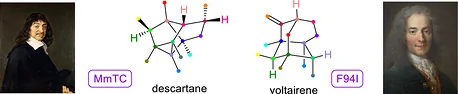

## 1 Introduction

**SCHEME 1:**
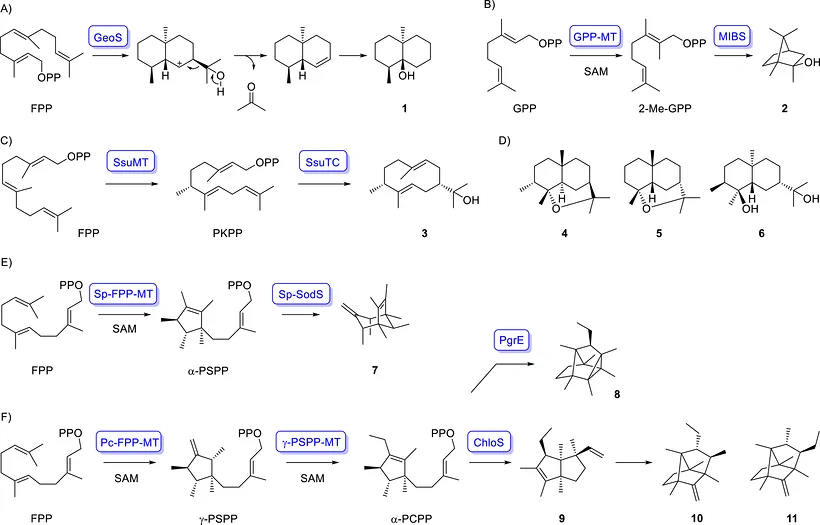
Biosynthesis of non‐canonical terpenes. Biosynthesis of (A) geosmin (**1**), (B) 2‐methylisoborneol (**2**), (C) weberol (**3**) and (D) structural revision of hegelenether (**4**). Biosynthesis of (E) sodorifen (**7**), and (F) chlororaphens A (**10**) and B (**11**) and grimophan (**8**).

Despite the enormous structural diversity of terpenes, their biosynthesis follows surprisingly simple principles. The two C_5_ building blocks, dimethylallyl pyrophosphate (DMAPP) and its double bond regioisomer isopentenyl pyrophosphate (IPP), account for the formation of thousands of terpenes [1, 2, 3]. Recent work has expanded this classical C_5n_ logic. Geosmin [4] (**1**, C_12_) is a degraded sesquiterpene that forms through loss of acetone in reactions catalysed by the bifunctional geosmin synthase GeoS (Scheme 1A). In this enzyme, a class I terpene cyclase (TC) domain is accompanied by a second domain that initiates fragmentation and elimination [5, 6, 7, 8]. In contrast, 2‐methylisoborneol [9] (**2**, C_11_, MIB) is produced by a different strategy. Geranyl pyrophosphate (GPP) is first methylated at C2 by an S‐adenosylmethionine (SAM)‐dependent methyltransferase (MT), followed by cyclisation catalysed by the terpene synthase MIBS (Scheme 1B) [10, 11, 12]. A related approach has also been described for farnesyl pyrophosphate (FPP) methylation at C6. This reaction yields the acyclic intermediate prekantenol pyrophosphate (PKPP), which is converted by TCs into eudesmane‐type homosesquiterpenes such as weberol (**3**) (Scheme 1C) [13]. Another reported product is hegelenether (**4**), exhibiting a seemingly impossible structure (Scheme 1D). A methylated derivative of evuncifer ether (**5**) [14] would be a realistic alternative, but a structural revision favoured the structure of marxdiol (**6**) [15].

Recent studies uncovered a further role of MTs in terpene biosynthesis. In these systems, a methyl transfer modifies a prenyl substrate and at the same time initiates a carbocation cascade within the active site. The first reported example is sodorifen [16] (**7**, C_16_), which is made by a methylation‐induced cyclisation of FPP by Sp‐FPP‐MT to yield α‐presodorifen pyrophosphate (α‐PSPP), followed by the sodorifen synthase (Sp‐SodS)‐catalysed cyclisation (Scheme 1E) [17]. Notably, α‐PSPP is accepted by several other TCs to generate a variety of C_16_ terpenes [18, 19]. Most homosesquiterpenes derived from α‐PSPP arise through a complex fragmentation‐recombination process uncovered through advanced isotopic labelling strategies in combination with computational approaches [20, 21]. This reactivity also extends to larger systems. In Pseudomonas chlororaphis, C_17_ terpenes are formed through methylation‐induced cyclisation of FPP to γ‐PSPP, followed by a second methylation to α‐prechlororaphen pyrophosphate (α‐PCPP). ChloS converts this intermediate via bicycloprechlororaphen (**9**) into chlororaphens A (**10**) and B (**11**), while related enzymes form other bishomosesquiterpenes such as grimophan (**8**) (Scheme 1F) [22, 23, 24, 25]. In recent work we and others have investigated first enzyme structural principles for the biosynthesis of methylated sesquiterpenes and demonstrated how structure‐based site‐directed mutagenesis can install functional switches in this highly interesting class of enzymes [15, 26, 27].

Building on this chemistry, here we present a new non‐canonical C_16_ sesquiterpene cyclase that produces the unusual compound descartane. Like grimophan, descartane lacks both an alkene and an alcohol function and instead contains a cyclopropane ring. This feature suggests a pathway involving terminal deprotonation coupled to cyclopropanation. We therefore investigated its cyclisation mechanism using a combined experimental and theoretical approach.

## 2 Results and Discussion

A phylogenetic analysis of 5000 amino acid sequences of bacterial TC homologs revealed a distinct clade of uncharacterised enzymes, with the anaximandrene synthases from Pseudomonas fluorescens FW300‐N2E3 and *Photorhabdus luminescens* DSM 3368 as the closest characterised relatives (Figure S1) [18, 21]. Notably, the corresponding genes are consistently located next to a gene homologous to the α‐PSPP‐forming methyltransferase Sp‐FPP‐MT (Figure S2) [17, 26]. This genomic organisation suggests that these enzymes act together with an MT and may therefore use α‐PSPP as substrate, pointing to the formation of new methylated sesquiterpenes. To test this hypothesis, we selected two putative non‐canonical TCs from Burkholderia sp. TSV86 (BsTC) and *Morganella morganii* AR_0133 (MmTC) for functional analysis. BsTC contains a slightly modified Asp‐rich motif (ARM) ^90^DDVFT^94^ [28], the pyrophosphate (PP) sensor Arg183 [29], a regular NSE triad ^227^NDLFSFRKE^235^ [30], and the ^315^RY segment [31] (Figure S3). It shares 93% sequence identity with heraclitene synthase from Burkholderia [18]. In contrast, MmTC exhibits the canonical ARM ^97^DDEYCD^102^, the PP sensor Arg188, and the ^322^RY segment, but a strongly modified NSE triad ^234^TEPHAYLKE^242^ (Figure S4). This enzyme shows 45% sequence identity to anaximandrene synthase from Pseudomonas fluorescens [21]. As demonstrated in this study, the replacement of Asn by Thr and Ser by Ala in the NSE triad of MmTC does not impair enzyme activity. This observation raises the question of how the third Mg^2+^ of the trinuclear cluster typically observed in class I TCs is coordinated or whether cofactor and substrate binding are differently organised in MmTC. Resolving this question will require future structural studies.

**SCHEME 2:**
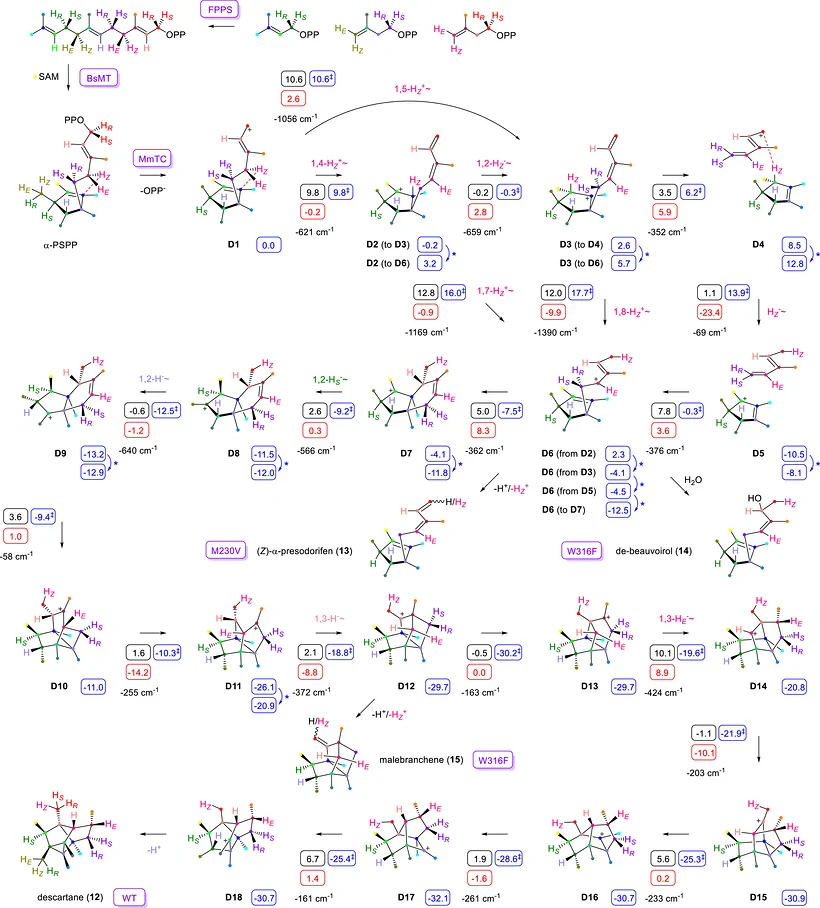
Biosynthesis of descartane (**12**) and related compounds **13**–**15**. For each compound the source enzyme variant is mentioned. All relevant hydrogens are specified in the structures of FPP, α‐PSPP and **12**, and are shown with the same colour as the carbon they originate from. Results of DFT calculations using wB97M‐V/Def2‐TZVPPD//B97D3/6‐31G (d, p) (298 K) are shown in boxes. Blue boxes indicate Gibbs energies relative to **D1** (‡ denotes free energies of transition states), black boxes show Gibbs reaction barriers, and red boxes indicate free reaction energies (all in kcal/mol). Imaginary frequencies of transition states are given in cm^−1^. Minor conformational changes between the product structure of one computed transformation and the starting structure of the next step are indicated by blue asterisks.

For functional characterisation, FPP and SAM were incubated with purified BsMT, which is known to produce α‐PSPP [20, 26], together with the two purified TC homologues (Figure S5). The cognate MmMT encoded near MmTC in the *M. morganii* locus was inactive under the experimental conditions and thus not used for functional characterisation of MmTC. Incubation with BsTC yielded heraclitene, thereby identifying this enzyme as a new heraclitene synthase and confirming the functionality of the in vitro system (Figure S6). In contrast, MmTC produced an unknown methylated sesquiterpene (Figure S7), which was isolated and identified by NMR spectroscopy (Figures S8–S15, Table S1) as descartane (**12**) (Scheme 2).

The biosynthesis of descartane (**12**) can be rationalised mechanistically via the cationic cascade shown in Scheme 2. Ionisation of α‐PSPP to **D1** is followed by a 1,4‐proton shift to **D2** and a subsequent 1,2‐hydride shift to **D3**. At this stage, fragmentation into a pentadienyl cation and a cyclopentene (**D4**) takes place. A hydride transfer between these fragments leads to **D5**, and recombination yields **D6**. This intermediate undergoes cyclisation to **D7**, followed by two successive 1,2‐hydride shifts to **D8** and **D9**. A second cyclisation and a dyotropic rearrangement furnish **D10** and **D11**, which rearranges with a simultaneous 1,3‐hydride transfer to **D12**. Subsequent skeletal rearrangement to **D13**, followed by a 1,3‐hydride shift to **D14** and four additional rearrangements (**D15**–**D18**), results in the precursor of **12**, which ultimately forms by deprotonation with concomitant cyclopropanation. Terminal cyclopropanation reactions are unusual in terpene biosynthesis but are relevant to a number of other fully saturated compounds ranging from mono‐ to sesterterpenes [32, 33, 34, 35, 36, 37, 38]. The mechanism of cyclopropane formation in terpene biosynthesis has so far not been studied in detail, but it likely requires an active site base such as pyrophosphate, a basic enzyme residue, or a water molecule.

This proposed mechanism for descartane was checked for its plausibility in all aspects through isotopic labelling experiments (Table 1 and Table S2). The experiments performed here make use of the detailed knowledge of α‐PSPP biosynthesis [20], as indicated by the colour code of labelled carbons and hydrogens from FPP and its precursors. Any viable mechanistic proposal must account for the experimentally determined carbon origins. This is the case for the mechanistic hypothesis for **12**, as demonstrated in incubation experiments with all 15 isotopomers of (^13^C)FPP [39] and (*methyl*‐^13^C)SAM, generated from (*methyl*‐^13^C)methionine using SalL from salinosporamide biosynthesis [40] (Figure S16). The biosynthesis of **12** was additionally investigated for all hydrogen migrations. This can generally be done by replacement of a migrating hydrogen by deuterium, with simultaneous substitution of its target position by ^13^C, which will result in a slightly upfield shifted triplet in the ^13^C‐NMR spectrum (Δ*δ* ≈ –0.4 ppm, ^1^ *J*_C,D_ ≈ 20 Hz). Alternatively, if a neighbouring carbon of the target position is substituted by ^13^C, an upfield shift of Δ*δ* ≈ –0.1 ppm will be observed, and even small isotope shifts can usually be detected if the substituted carbon is two positions away from the target position (Δ*δ* ≈ –0.01 ppm).

**TABLE 1:** Summary of isotopic labelling experiments on the biosynthesis and absolute configuration of **12**.

Experiment	Results shown in	Effect	Interpretation
Individual labelling of the 16 carbons	Figure S16	enhanced ^13^C‐NMR signals of **12**	Biosynthetic origin of the C backbone
GPP plus (*E*)‐(4‐^13^C,4‐^2^H)IPP, SAM, FPPS, BsMT, MmTC	Figure S17	C4: Δ*δ* = –0.1 ppm	^2^H in neighbouring position to C4
GPP plus (*Z*)‐(4‐^13^C,4‐^2^H)IPP, SAM, FPPS, BsMT, MmTC	Figure S18	C4: Δ*δ* = –0.02 ppm	^2^H two positions away from C4
GPP plus (1‐^13^C,4,4,4,5,5,5‐^2^H_6_)DMAPP, SAM, IDI, FPPS, BsMT, MmTC	Figure S19	C1: Δ*δ* = –0.34 ppm, ^1^ *J*_C,D_ = 19.1 Hz	^2^H migrates from C4 to C1
(2‐^13^C,1,1‐^2^H_2_)DMAPP plus IPP, SAM, FPPS, BsMT, MmTC	Figure S20	C10: Δ*δ* = –0.54 ppm, ^1^ *J*_C,D_ = 18.9 Hz	^2^H migrates from C9 to C10
(2‐^2^H)GPP plus IPP, SAM, FPPS, BsMT, MmTC	Figure S21	Same MS as for unlabelled **12**	H6 is lost in the cyclopropanation
(3‐^13^C,2‐^2^H)FPP plus SAM, BsMT, MmTC	Figure S22	C3: Δ*δ* = –0.03	^2^H is one or two positions away from C3
(*R*)‐ or (*S*)‐(1‐^13^C,1‐^2^H)GPP plus IPP, SAM, FPPS, BsMT, MmTC	Figure S23	Stereoselective deuteration (HSQC)	Absolute configuration of **12**

The hydrogens at C4 of FPP can be stereoselectively labelled using the substrate combination of GPP and (*E*)‐ or (*Z*)‐(4‐^13^C,4‐^2^H)IPP [41] with *Streptomyces coelicolor* FPP synthase (FPPS) [42]. If SAM, BsMT and MmTC are added to the same pot, the conversion will proceed towards labelled **12**. The experiment with (*E*)‐(4‐^13^C,4‐^2^H)IPP yielded a slightly shielded singlet (Δδ = –0.1 ppm), indicating the presence of deuterium in a neighbouring position (Figure S17). This can only be C3, because the other two neighbours of C4 (C2 and C7) are quaternary. Substitution of H4*_Z_* using (*Z*)‐(4‐^13^C,4‐^2^H)IPP returned a singlet with a small, but clear upfield shift (Δ*δ* = –0.02 ppm), being located two positions away (Figure S18). The target position was identified through an incubation of GPP, (1‐^13^C,4,4,4,5,5,5‐^2^H_6_)DMAPP [20] and SAM with *E. coli* isopentenyl diphosphate isomerase (IDI) [43], FPPS, BsMT and MmTC, leading to an upfield shifted triplet for C1 (Δ*δ* = –0.34 ppm, ^1^ *J*_C,D_ = 19.1 Hz; Figure S19). These data are in agreement with the proposed complex migrations of H4*_Z_* in the initial steps towards **D6** and the 1,3‐hydride shift of H4*_E_* from **D13** to **D14**.

The 1,2‐hydride shift from **D7** to **D8** was investigated using (2‐^13^C,1,1‐^2^H_2_)DMAPP [42], IPP and SAM with FPPS, BsMT, and MmTC, which resulted in an upfield shifted triplet for C10 (Δδ = –0.54 ppm, ^1^ *J*_C,D_ = 18.9 Hz; Figure S20). It has been demonstrated in previous work that H9*_R_* migrates into the Me group C8 of α‐PSPP [20], and thus it is H9*_S_* that is bound to C10 in **12**. The next 1,2‐hydride transfer from **D8** to **D9** cannot be visualised, because the migrating H6, known to be located at C7 in α‐PSPP, is lost in the final deprotonation to **12**, as is evident from an incubation of (2‐^2^H)GPP [44], IPP and SAM with FPPS, BsMT and MmTC (Figure S21). Finally, the proposed 1,3‐hydride shift from **D11** to **D12** was supported through an enzymatic conversion of (3‐^13^C,2‐^2^H)FPP [45] and SAM with BsMT and MmTC, revealing a slightly shielded singlet for C3 of **12** (Δ*δ* = –0.03; Figure S22). This finding is consistent with deuterium being located one or two positions away from C3. Two bonds away from C3, only quaternary carbons are present (C2, C7, and C11), ruling out this possibility. Regarding direct neighbours, the experiments discussed below for the determination of the absolute configuration of **12** confirm that both hydrogens from C5 stay at this carbon, and it is reasonable to assume that Me15 remains unchanged. Therefore, C4 is the only possible target for H2.

The absolute configurations of terpenes can be determined through stereoselective deuterations in CH_2_ groups that remain unchanged during terpene cyclisation. Upon TC catalysis, enantiotopic hydrogens in FPP will turn into diastereotopic hydrogens in the product, which can be discriminated through nuclear Overhauser spectroscopy (NOESY). Accordingly, artificially introduced stereogenic centers of known configuration in stereoselectively deuterated CH_2_ groups serve as anchors and allow assignment of the absolute configuration of a terpene by determination of the relative configuration with respect to the deuterated anchor. In the biosynthesis of **12**, including the fragmentation pathway **D4**–**D5** none of the CH_2_ groups of FPP remain unchanged. If the fragmentation is skipped through direct reactions from **D2** or **D3** to **D6**, C5 would be the only suitable anchor for stereoselective deuteration experiments. However, for the fragmentation pathway, a clear stereochemical course for the fragmentation to **D4** and the recombination of the fragments to **D6** can be assumed that should result in the same configuration at C5 in **D6** as for any direct formation from **D2** or **D3**. Any change in the configuration at C5 would require a change of the relative orientation of the fragments towards each other, leading to a different face selectivity at C5 in the recombination. This would require turning of the whole upper fragment, or at least of the vinyl group C4–C5, by 180°, for which no evidence is available. With these considerations and the known configurational inversion at C1 in the elongation of oligoprenyl diphosphates with IPP [46], the incubation of (*R*)‐ and (*S*)‐(1‐^13^C,1‐^2^H)GPP [47], IPP and SAM with FPPS, BsMT and MmTC allowed the assignment of the absolute configuration of **12** as shown (Figure S23). In these experiments, the additional ^13^C‐label allowed for a sensitive detection of deuterium incorporation through heteronuclear single quantum correlation (HSQC).

The biosynthesis of **12** was further studied through DFT computations (Scheme 2, Table S3, Figure S24). For the proton transfer of H4*_Z_* from C4 to the cyclopentene, a two‐step process is energetically preferred over a direct reaction from **D1** to **D3** (activation barrier of 9.8 kcal/mol vs. 10.6 kcal/mol), as previously reported for sodorifen biosynthesis [20]. Notably, the fragmentation pathway to **D6** is energetically favoured over any direct formation from **D2** or **D3** (the highest reaction barriers are 13.9 kcal/mol vs. 16.0 kcal/mol and 17.7 kcal/mol above the level of **D1**). It should be emphasised that labelling experiments cannot distinguish between the different possibilities at this point. All downstream transformations reveal small activation barriers or are barrierless, with only one step from **D13** to the secondary cation **D14** exhibiting a barrier above 10 kcal/mol.

**FIGURE 1:**
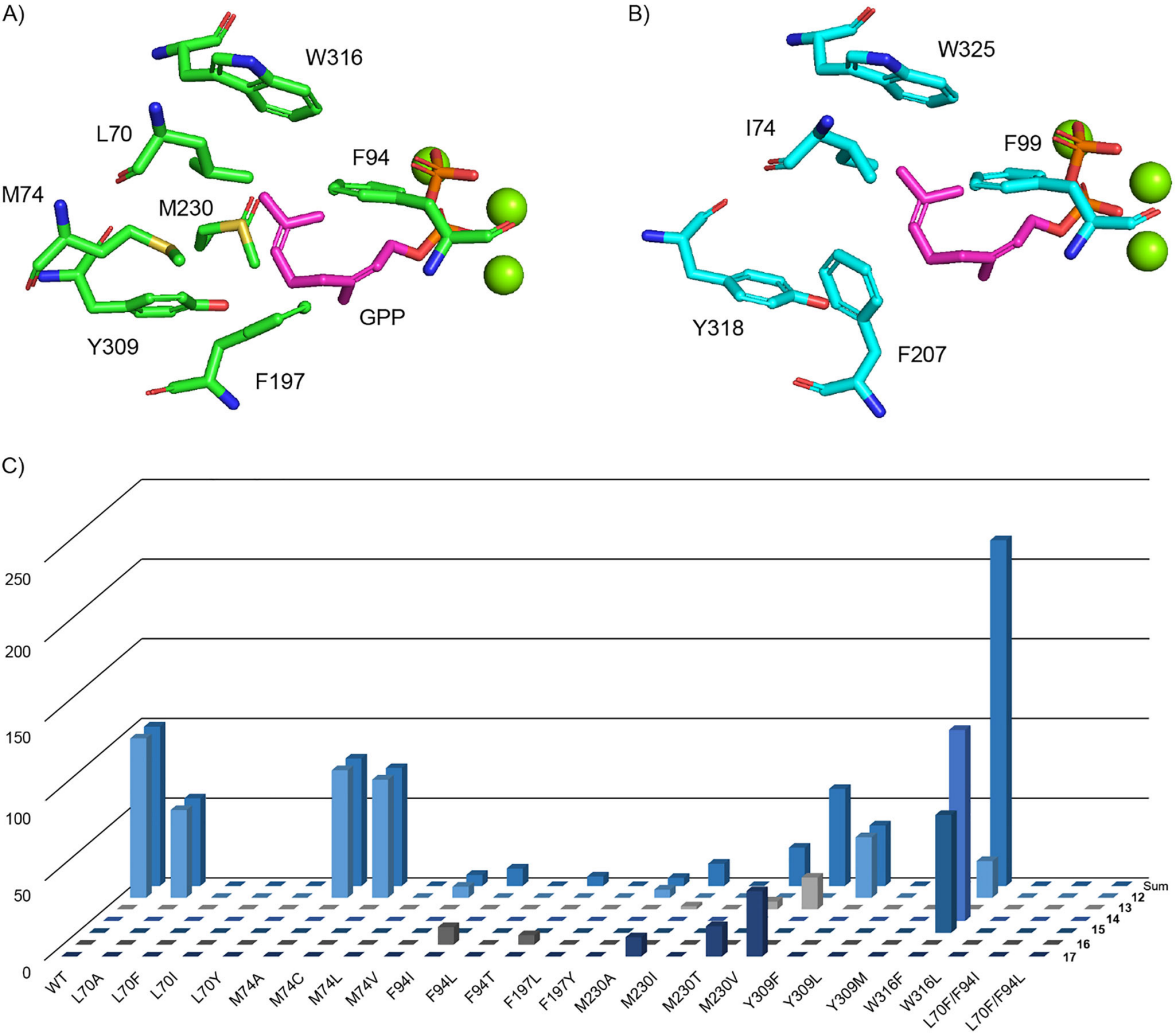
Site‐directed mutagenesis of MmTC. (A) Active site residues identified from an AlphaFold3 model are aligned to the x‐ray structure of Sp‐SodS (PDB: 2I1I) [27]. GPP and the three Mg^2+^ cations are taken from the Sp‐SodS structure. (B) Active site of Sp‐SodS for comparison. The indicated residues were mutated in previous work [27]. (C) Product formation by MmTC variants relative to the production of **12** by wild‐type MmTC (set to 100%). Bars represent mean values from triplicates; standard deviations are given in Table S5.

These results indicate that the overall reaction pathway is largely governed by the intrinsic reactivity of the carbocation intermediates, while the enzyme environment controls pathway selection and product outcome. To probe this influence, we investigated MmTC by site‐directed mutagenesis. Recent structure‐based studies on Sp‐SodS have shown that mutations of active site residues can lead to the formation of new terpene products (Figure 1B) [27]. Although these compounds have not yet been fully characterised, the findings point to pronounced active site plasticity and suggest that small structural changes can redirect the cyclisation cascade. Guided by these observations and the close structural similarity between Sp‐SodS and MmTC, active site residues in MmTC were selected for mutagenesis based on an AlphaFold3 model [48] in comparison with the x‐ray structure of Sp‐SodS in complex with GPP and three Mg^2+^ cations [27]. The model aligns well with MmTC, with a root mean square deviation (RMSD) of 1.6 Å across 303 C^α^ atoms. Targeted positions included Leu70, Met74, Phe94, Phe197, Met230, Tyr309, and Trp316 (Figure 1A). The L70A variant showed reduced product formation (55% ± 2% of wild‐type level), whereas L70F, L70I, and L70Y were inactive. The inactivity of L70F is notable, since some closely related enzymes naturally contain a Phe residue at this position, suggesting that activity depends on additional substitutions. Substitutions at Met74 revealed minor effects for M74A (80% ± 9%) and M74C (74% ± 22%) but strongly reduced activity for M74V (7% ± 1%) and complete loss of activity for M74L.

Interesting results were obtained for mutations at Phe94. The F94L variant was inactive, whereas substitutions to Ile and Thr resulted in the formation of a new compound in low amounts (11% ± 0.2% and 6% ± 1%, respectively). This product was isolated and identified as the new compound voltairene (**16**) (Figures S26–S34, Table S6). The sequence analysis revealed a clear pattern. Enzymes closely related to MmTC that contain Leu70 consistently exhibit Phe94, whereas those with Phe70 carry Ile94, indicating conservation of one aliphatic and one aromatic residue at these positions (Figure S35). Notably, these residues are located next to each other (Figure 1), and the inactivity of the L70F variant can be explained by a steric clash between Phe70 and Phe94. The functional switch to a voltairene synthase, combined with the reduced activity of the F94I variant, prompted us to test whether a double substitution could restore higher production levels. Surprisingly, this was not the case, and both the L70F‐F94I and L70F‐F94L double mutants were inactive, demonstrating that the outcome of this rationally designed variant remains unpredictable.

Mutations at Phe197 resulted in a strongly reduced productivity. The variants F197L and F197Y showed no detectable formation of **12** and only trace amounts of additional products that could not be isolated. Substitutions at Met230 were also critical for catalysis. M230I was inactive, whereas M230V, M230T, and M230A produced two new compounds. The M230V variant exhibited the highest productivity (41% ± 3% and 20% ± 2% relative production, Figure S36), allowing isolation and identification of known pythagorene (**17**) [18] and (*Z*)‐α‐presodorifen (**13**) [18]. The Y309F variant retained reduced production (38% ± 7%), whereas Y309L and Y309M were inactive, indicating an essential role of this position. The W316L variant was inactive, while W316F produced two new compounds in high amounts (74% ± 13% and 120% ± 22%, Figure S37), in addition to residual formation of **12** (23% ± 4%). These products were isolated and identified as the new compounds malebranchene (**15**) and de‐beauvoirol (**14**) (Figures S38–S52, Tables S7 and S8). Taken together, the results of these mutagenesis experiments revealed a particularly important role of aromatic residues in the active site (Phe94, Phe197, Tyr309 and Trp316). These residues may be involved in the stabilisation of intermediates through cation‐π interactions, explaining the dramatic effects of their exchange against non‐aromatic residues, while replacements by other aromatic residues usually resulted in a maintained or even improved performance.

**SCHEME 3:**
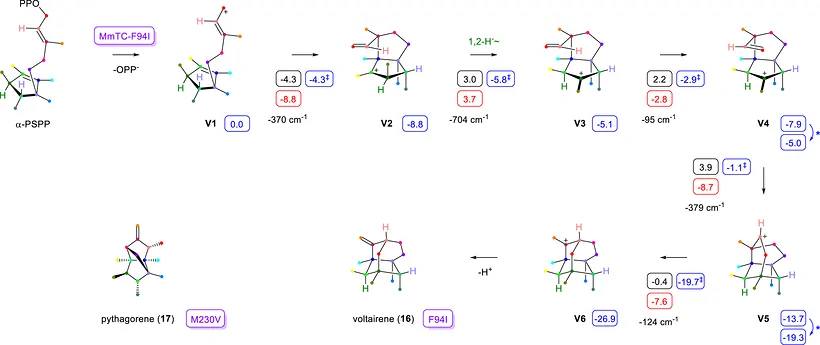
Biosynthesis of voltairene (**16**) and structure of pythagorene (**17**) (for its biosynthesis cf. ref [18]). For explanations regarding the computational data, compare the legend of Scheme 2.

The formation of these products can be rationalised on the basis of cationic intermediates along the pathway to **12** (Scheme 2). Compound **13** arises from **D6** by deprotonation, whereas de‐beauvoirol (**14**) is formed from the same intermediate by attack of water. Malebranchene (**15**) closely follows the pathway to descartane and derives from the late intermediate **D12**. A modified model explains voltairene (**16**) biosynthesis that follows a more direct pathway (Scheme 3). After ionisation to **V1**, a 3,10‐cyclisation leads to **V2**, which undergoes a 1,2‐hydride shift to **V3**. Rotation of the vinyl group results in **V4**, followed by cyclisation to **V5**, skeletal rearrangement to **V6**, and final deprotonation to yield **16**. This mechanism is also supported computationally by the determined low reaction barriers for all elementary steps (Table S9, Figure S53).

## 3 Conclusions

In summary, we have identified a new non‐canonical terpene synthase MmTC from *Morganella morganii* that produces the structurally unique and fully saturated compound descartane from α‐presodorifen diphosphate. Our results provide a coherent picture of its enzymatic formation through combined experimental and computational data that support a sophisticated cyclisation cascade. The unusual reaction sequence suggested in this study involves fragmentation‐recombination steps, proceeds through a total of 18 cationic intermediates and culminates in cyclopropane formation. Enzyme variants obtained through site‐directed mutagenesis yielded related compounds, revealing the role of key active site residues in controlling product formation. Several of the newly obtained compounds represent deprotonation products of pathway intermediates, giving additional support for the proposed unusually long carbocation cascade towards descartane. While all experimental and computational data obtained in this study are fully consistent with the proposed mechanism of descartane biosynthesis, further investigations may provide additional insights into this unusual cyclisation cascade. We will continue our mechanistic investigations on other non‐canonical terpene pathways to uncover additional mechanistic riddles in our future work.

## Author Contributions

**Kexin Yang**: investigation, writing – review and editing, visualization, data curation. **Philip Troycke**: investigation, methodology, validation. **Bernd Goldfuss**: investigation, data curation, resources, writing – review and editing, methodology. **Michael Groll**: conceptualization, funding acquisition, writing – review and editing, formal analysis, project administration, supervision, resources. **Jeroen S. Dickschat**: conceptualization, funding acquisition, writing – original draft, project administration, supervision, resources, visualization.

## Conflicts of Interest

The authors declare no conflicts of interest.

## Supporting information



The authors have cited additional references within the Supporting Information [49, 50, 51, 52, 53, 54, 55, 56, 57, 58, 59, 60, 61, 62, 63, 64, 65, 66].
**Supporting File**: anie73480‐sup‐0001‐SuppMat.docx.

## Data Availability

The data that supports the findings of this study are available in the Supporting Information of this article.
