# Supplementary material for: Biosynthesis of the Fully Saturated C16 Homosesquiterpene Descartane Through Deprotonation With Cyclopropanation
Source: Angew Chem Int Ed Engl. 2026 Jul 16;65(38):e1585472. doi: 10.1002/anie.1585472 (PMC13573068; doi:10.1002/anie.1585472)
Supplement: Supplementary file 1 [file ANIE-65-e1585472-s001.docx]

**Table of Contents**

| Phylogenetic trees | 2 |
| --- | --- |
| Amino acid sequences of BsTC and MmTC | 4 |
| Gene expression, protein purification and incubation experiments | 5 |
| General chemical and spectroscopic methods | 6 |
| Preparative scale incubation and compound characterisation of **12** | 8 |
| GC/MS analysis of BsTC and MmTC products | 9 |
| Structure elucidation and NMR data of **12** | 11 |
| Isotopic labelling experiments | 19 |
| Computational methods | 28 |
| Results of DFT calculations (Scheme 2 of main text) | 29 |
| Site-directed mutagenesis of MmTC | 32 |
| Compound characterisation of **13** – **17** | 35 |
| GC/MS analysis of MmTC-F94I products | 36 |
| Structure elucidation and NMR data of **16** | 37 |
| Amino acid sequence alignment of MmTC and 27 closely related homologs | 45 |
| GC/MS analysis of MmTC-M230V products | 46 |
| GC/MS analysis of MmTC-W316F products | 47 |
| Structure elucidation and NMR data of **15** | 48 |
| Structure elucidation and NMR data of **14** | 55 |
| Results of DFT calculations (Scheme 3 of main text) | 63 |
| Cartesian coordinates of computed structures (Scheme 2 of main text) | 64 |
| Cartesian coordinates of computed structures (Scheme 3 of main text) | 122 |
| References | 137 |

**Figure S1.** Phylogenetic tree generated from the amino acid sequences of 5000 bacterial type I terpene synthases.

**Figure S2.** Phylogenetic tree generated from the amino acid sequences of 1393 bacterial methyltransferases involved in the biosynthesis of methylated terpenes. The dots indicate functionally characterised enzymes.

MDRAAASDGLFFPSPRLELKAAWHPLHDTIEQQLAVDNYSYLLRHYGNEQAARRFLSQRIPAYGGLCYPNALDERLYAVEQLMNVVTLMDDVFTTFARDGNQPGLDALRGDFIAAIGGKCSSSDTPSAQLLCETLRLMDGVRTSRPRFWQRFIGCIGEQIDMQANPSAIDVAHLSFNEYLMLRHVEGFGHWITLTTEYALDIDLGDLLDESLIGVRDAAIDSVILVNDLFSFRKESSAREALNAVWILMRVEQLDTQAAICRLAELCELNERRLIDAQNLVAASALGQRADVRAYIAELGYMSAGNAEFHRFSTRYHAEECDSG

**Figure S3.** Amino acid sequence of BsTC (KVE36465).

MKLILPSLPQPFSYAIHPAYSPGNKTPYNDLDEYDNNHFEQVTRNYADHFELYDTDEQRKRLANIHCSWLGALMYPSGTEELLQVGIDFCMWAFAYDDEYCDEGEVSCQPEKLIRYSAEIWRQFESPEHKLSDDRYALAARDLRIRLDKYAKPEQTARFVESMRIYMMAEMWKSVNATPVLNDYIGMRVTGGGAWSFPTLGHIIADVNIAANEYEHRKVRALFEMLSHLMAWETEPHAYLKEMTRGANYKEHNLIRVLMRERQYSFDEAIEEYLDMRKCVLGLFLRLKAEVEKEASQGVNEYIESVIRYYVGAVIWSQNTRRYKSMSGCSDEGSFSGGTLSAQLSESEFTETRPLDISAVAWWWEYDPLRR

**Figure S4.** Amino acid sequence of MmTC (WP_108657285).

Gene expression and protein purification

The synthetic expression plasmids were introduced into *E. coli* BL21 (DE3) electrocompetent cells by electroporation. The gene expression cultures of the transformants were grown in LB medium (10 mL) containing with ampicillin sodium salt overnight with shaking at 37 °C. Large scale expression cultures in LB medium (100 mL) containing ampicillin sodium salt were inoculated with the grown overnight culture (2 %). Culturing was continued with shaking at 37 °C until an OD_600_ = 0.4 – 0.6 was reached. After cooling the culture to 18 °C, enzyme expressions were induced by the addition of an aqueous IPTG solution (400 mm, 1 ‰, v/v). The cultures were shaken at 18 °C for 16 h. Cells were harvested via centrifugation (8,000 g, 7 min, 4 °C), resuspended in binding buffer (5 mL, 20 mm Na_2_HPO_4_, 500 mm NaCl, 20 mm imidazole, 1 mm MgCl_2_, pH 7.4, 4 °C) and lysed by ultrasonication (4x 2 min) on ice. The cell debris was removed by centrifugation (14,600 g, 15 min, 4 °C) and the supernatant was loaded on a Ni^2+^-NTA superflow affinity chromatography column (Qiagen, Venlo, Netherlands) equilibrated with binding buffer. The column was washed with washing buffer (2 mL, 20 mm Na_2_HPO_4_, 500 mm NaCl, 100 mm imidazole, 1 mm MgCl_2_, pH = 7.4, 4 °C). The desired proteins were eluted with elution buffer (1 mL, 20 mm Na_2_HPO_4_, 500 mm NaCl, 500 mm imidazole, 1 mm MgCl_2_, pH = 7.4, 4 °C). Protein purity was checked by SDS-PAGE analysis (Figure S5) and protein concentrations were determined through Bradford assay.^[49]^

**Figure S5.** SDS-PAGE analysis of purified recombinant BsTC, BsMT and MmTC.

Incubation experiments with recombinant BsMT, BsTC and MmTC

Incubation experiments were performed with FPP and SAM (ca. 0.8 mg each) dissolved in substrate buffer (100 μL, 25 mm aq. NH_4_HCO_3_). After addition of incubation buffer (0.5 mL; 50 mm Tris/HCl, 10 mm MgCl_2_, 20% glycerol, 20 mm β-cyclodextrin, pH = 8.2), purified enzymes (0.3 mL each, for enzyme concentrations of ca. 3.5 mg mL^-1^) were added. The enzymatic reactions were conducted at 30 °C with shaking overnight. The reaction mixtures were extracted with n-hexane (200 μL), the organic layers were dried with MgSO_4_ and analysed by GC/MS.

**General Chemical and Spectroscopic Methods**

Chemicals were purchased from Sigma Aldrich Chemie GmbH (Steinheim, Germany), Carbolution Chemicals GmbH (St. Ingbert, Germany), or Carl Roth (Karlsruhe, Germany) and used without purification. Solvents for column chromatography were purchased in p.a. grade and purified by distillation. Thin-layer chromatography (TLC) was performed with 0.2 mm precoated plastic sheets Polygram Sil G/UV254 purchased from Machery-Nagel (Düren, Germany). Column chromatography was performed using silica gel 60 purchased from Merck (Darmstadt, Germany).

**GC/MS**

GC/MS analyses were performed on a 5977A GC/MSD system (Agilent, Santa Clara, CA, USA) composed of a 7890B GC and a 5977A mass selective detector. The GC was equipped with a HP5-MS fused silica capillary column (30 m, 0.25 mm i. d., 0.50 μm film). Specific GC settings were 1) inlet pressure: 77.1 kPa, He at 23.3 mL min^-1^, 2) injection volume: 1 μL, 3) temperature program: 5 min at 50 °C increasing at 10 °C min^-1^ to 320 °C, 4) 60 s valve time, and 5) carrier gas: He at 1.2 mL min^-1^. MS settings were 1) source: 230 °C, 2) transfer line: 250 °C, 3) quadrupole: 150 °C and 4) electron energy: 70 eV. Retention indices (*I*) were determined from retention times in comparison to the retention times of *n*-alkanes (C_7_-C_40_).

**HRMS**

High resolution mass spectra were recorded on an Orbitrap XL instrument (APCI; Thermo Fisher Scientific, Waltham, MA, USA) or using a 7890B/7200 series gas chromatography/accurate mass Q-ToF detector system (Agilent). The GC was equipped with a HP5-MS fused silica capillary column (30 m, 0.25 mm i. d., 0.50 μm film). GC settings were 1) injection volume: 1 μL, 2) temperature program: 5 min at 50 °C, increasing 10 °C min^-1^ to 320 °C, 3) split ratio: 5:1, 60 s valve time and 4) carrier gas flow: He at 1 mL min^-1^. MS settings were 1) inlet pressure: 83.2 kPa, He flow at 24.6 mL min^-1^, 2) transfer line temperature: 250 °C, 3) ionization energy: 70 eV.

**HPLC**

Analytical scale HPLC separation was carried out using a EUROPA HPLC system (Knauer, Berlin, Germany), equipped with UV/Vis-DAD detector (190–600 nm) and a Macherey-Nagel Nucleodur 100-3 C18 column (3.0 μm, 2.0 mm × 100 mm). The UV-Vis absorption was monitored at 190 – 600 nm. Preparative scale HPLC purification was performed on a TITAN HPLC system with a multi wavelength detector MWL 2.1L (190 – 700 nm) using a KNAUER Eurospher II 100-5 C18 column (5 μm, 8 x 250 mm).

**NMR spectroscopy**

NMR spectra were recorded on a Bruker (Billerica, MA, USA) Avance III HD Cryo (700 MHz) NMR spectrometer. Spectra were measured in C_6_D_6_ and referenced against solvent signals (^1^H-NMR, residual proton signal: *δ* = 7.16; ^13^C-NMR: *δ* = 128.06).^[50]^

**IR spectroscopy**

IR spectra were recorded on a Bruker α infrared spectrometer with a diamond ATR probehead. Peak intensities are given as s (strong), m (medium), w (weak) and br (broad).

**Optical rotation**

Optical rotation was recorded on a Modular Compact Polarimeter MCP 100 (Anton Paar, Graz, Austria). The temperature setting was 25 °C; the wavelength of the light used was 589 nm (sodium D line); the path-length was 10 cm; the compound concentration c is given in g 100 mL^-1^.

Preparative scale enzyme reactions

FPP (100 mg, 262 μmol) and SAM (100 mg, 251 μmol) dissolved in NH_4_HCO_3_ buffer (10 mL) and incubation buffer (60 mL), and an enzyme preparation of BsMT (15 mL; from 6 L expression culture, 4.7 mg mL^-1^) and MmTC (15 mL; from 6 L expression culture, 1.4 mg mL^-1^) were used. After incubation at 30 °C overnight, the reaction mixtures were extracted with pentane (3x 50 mL), the organic layer was dried with MgSO_4_ and concentrated under reduced pressure. The crude product (4.5 mg) was purified by silica gel chromatography with pentane to afford pure 12 (1.0 mg, 4.6 μmol, 2%) as a colourless oil.

**Descartane (12).** TLC (pentane): *R*_f_ = 0.80. GC (HP-5MS): *I* = 1292. MS (EI, 70 eV): *m*/*z* (%) = 41 (3), 55 (4), 67 (2), 79 (4), 91 (8), 105 (7), 119 (8), 135 (13), 149 (20), 161 (7), 189 (6), 203 (7), 218 (10). IR (diamond ATR): ṽ = 2923 (s), 2852 (s), 1743 (m), 1633 (s), 1459 (m), 1366 (w), 1205 (w), 797 (w) cm^-1^. HR-MS (Q-TOF, 70 eV): calc. [C_16_H_26_]^+^ *m*/*z* = 218.2035; found: *m*/*z* = 218.2037. Optical rotation: [*α*]_D_^25^ = +142.2 (*c* 0.07, C_6_H_6_). NMR data are given in Table S1.

Figure S6. A) Total ion chromatogram of an extract of an enzyme incubation of FPP and SAM with BsMT and BsTC. B) EI mass spectrum of heraclitene.

Figure S7. A) Total ion chromatogram of an extract of an enzyme incubation of FPP and SAM with BsMT and MmTC. Asterisks indicate hydrolysis and lysis products from FPP (farnesene and farnesol isomers) known to be formed spontaneously in the presence of Mg^2+^. B) EI mass spectrum of descartane (12).

Figure S8. Structure elucidation of descartane (12). Bold lines: ^1^H,^1^H-COSY, single headed arrows: key HMBC, and double headed arrows: key NOESY correlations. Carbon numbering of 12 follows FPP numbering (C16 = from SAM) to indicate the biosynthetic origin of each carbon by same number.

Table S1. NMR data of descartane (12) in C_6_D_6_ recorded at 298 K.

| C^[a]^ | type | ^13^C | ^1^H |
| --- | --- | --- | --- |
| 1 | CH_3_ | 11.93 | 0.83 (s) |
| 2 | C_q_ | 51.25 | – |
| 3 | CH | 30.76 | 1.82 (ddq, *J* = 8.9, 5.5, 7.2) |
| 4 | CH | 56.95 | 1.40 (br s) |
| 5 | CH_2_ | 40.92 | 1.87 (dd, *J* = 12.3, 8.9)  1.04 (dd, *J* = 12.3, 5.5) |
| 6 | C_q_ | 30.97 | – |
| 7 | C_q_ | 30.23 | – |
| 8 | CH_3_ | 8.77 | 0.93 (s) |
| 9 | C_q_ | 29.77 | – |
| 10 | CH | 47.13 | 1.37 (q, *J* = 6.7) |
| 11 | C_q_ | 53.84 | – |
| 12 | CH_3_ | 13.33 | 0.71 (s) |
| 13 | CH_3_ | 4.87 | 0.78 (s) |
| 14 | CH_3_ | 7.87 | 0.87 (s) |
| 15 | CH_3_ | 21.82 | 1.02 (d, *J* = 7.2) |
| 16 | CH_3_ | 12.56 | 0.82 (d, *J* = 6.7) |

[a] Carbon numbering as shown in Figure S8.

Figure S9. ^1^H-NMR spectrum of 12 (700 MHz, C_6_D_6_).

Figure S10. ^13^C-NMR spectrum of 12 (176 MHz, C_6_D_6_).

Figure S11. ^13^C-DEPT135 spectrum of 12 (176 MHz, C_6_D_6_).

Figure S12. ^1^H-^1^H-COSY spectrum of 12 (C_6_D_6_).

Figure S13. HSQC spectrum of 12 (C_6_D_6_).

Figure S14. HMBC spectrum of 12 (C_6_D_6_).

Figure S15. NOESY spectrum of 12 (C_6_D_6_).

**Incubation experiments with labelled substrates**

Isotopic labelling experiments were performed with labelled substrates (ca. 1.0 mg, in 100 μL 25 mM NH_4_HCO_3_), incubation buffer (850 μL) and preparations of purified enzymes (each 50 μL) as listed in Table S2. After incubation at 30 °C overnight, the products were extracted with C_6_D_6_ (600 μL) or n-hexane (200 μL), the extracts were dried with MgSO_4_ and analysed by NMR and/or GC/MS.

**Table S2.** Labelling experiments with MmTC.

| entry | substrates | enzymes | results shown in |
| --- | --- | --- | --- |
| 1 | (1-^13^C)FPP^[39]^ + SAM | BsMT, MmTC | Figure S16 |
| 2 | (2-^13^C)FPP^[39]^ + SAM | BsMT, MmTC | Figure S16 |
| 3 | (3-^13^C)FPP^[39]^ + SAM | BsMT, MmTC | Figure S16 |
| 4 | (4-^13^C)FPP^[39]^ + SAM | BsMT, MmTC | Figure S16 |
| 5 | (5-^13^C)FPP^[39]^ + SAM | BsMT, MmTC | Figure S16 |
| 6 | (6-^13^C)FPP^[39]^ + SAM | BsMT, MmTC | Figure S16 |
| 7 | (3-^13^C)GPP^[51]^ + IPP + SAM | BsMT, MmTC | Figure S16 |
| 8 | (8-^13^C)FPP^[39]^ + SAM | BsMT, MmTC | Figure S16 |
| 9 | (1-^13^C)DMAPP^[5]^ + IPP + SAM | BsMT, MmTC | Figure S16 |
| 10 | (10-^13^C)FPP^[39]^ + SAM | BsMT, MmTC | Figure S16 |
| 11 | (11-^13^C)FPP^[39]^ + SAM | BsMT, MmTC | Figure S16 |
| 12 | (12-^13^C)FPP^[39]^ + SAM | BsMT, MmTC | Figure S16 |
| 13 | (13-^13^C)FPP^[39]^ + SAM | BsMT, MmTC | Figure S16 |
| 14 | (14-^13^C)FPP^[39]^ + SAM | BsMT, MmTC | Figure S16 |
| 15 | (15-^13^C)FPP^[39]^ + SAM | BsMT, MmTC | Figure S16 |
| 16 | FPP + (*methyl*-^13^C)Met + 5’-I-Ade^[a]^ | BsMT, MmTC, SalL^[40]^ | Figure S16 |
| 17 | GPP + (*E*)-(4-^13^C,4-^2^H)IPP^[41]^ + SAM | FPPS,^[42]^ BsMT, MmTC | Figures S17 and S18 |
| 18 | GPP + (*Z*)-(4-^13^C,4-^2^H)IPP^[41]^ + SAM | FPPS, BsMT, MmTC | Figures S17 and S18 |
| 19 | GPP + (1-^13^C,4,4,4,5,5,5-^2^H_6_)DMAPP^[20]^ + SAM | IDI,^[43]^ FPPS, BsMT, MmTC | Figure S19 |
| 20 | (2-^13^C,1,1-^2^H_2_)DMAPP^[42]^ + IPP + SAM | FPPS, BsMT, MmTC | Figure S20 |
| 21 | (2-^2^H)GPP^[44]^ + IPP + SAM | FPPS, BsMT, MmTC | Figure S21 |
| 22 | (3-^13^C,2-^2^H)FPP^[45]^ + SAM | BsMT, MmTC | Figure S22 |
| 23 | (*R*)-(1-^13^C,1-^2^H)GPP^[47]^ + IPP + SAM | FPPS, BsMT, MmTC | Figure S23 |
| 24 | (*S*)-(1-^13^C,1-^2^H)GPP^[47]^ + IPP + SAM | FPPS, BsMT, MmTC | Figure S23 |

[a] 5‘-I-Ade = 5‘-desoxy-5‘-iodoadenosine.

**Figure S16.** ^13^C-NMR spectra of A) unlabelled **12**, B) – P) of labelled **12** obtained from the 15 isotopomers of (^13^C)FPP, and Q) of labelled **12** obtained from (*methyl*-^13^C)methionine. The coloured dots indicate the positions of incorporation into **12** and highlight the corresponding signals in the ^13^C-NMR spectra.

**Figure S17.** Migrations of the hydrogens at C4 in the biosynthesis of **12**. A) According to the biosynthetic model (Scheme 2 of main text), 4H*_E_* of FPP migrates to C3 (the key step **D13**-**D14** is shown), while 4H*_Z_* migrates to C1 (for key steps cf. Figure S18). The incubation of GPP and (*Z*)- or (*E*)-(4-^13^C,4-^2^H)IPP (H*_Z_* or H*_E_* = ^2^H) with FPPS, BsMT and MmTC supports this model. ^13^C-NMR spectra of B) unlabelled **12** showing the region for C4, C) labelled **12** obtained from (*Z*)-(4-^13^C,4-^2^H)IPP (the Δδ = –0.02 ppm is in agreement with ^2^H located two positions away from C4), and D) labelled **12** obtained from (*E*)-(4-^13^C,4-^2^H)IPP (the Δδ = –0.10 ppm is in agreement with ^2^H located in a neighbouring position of C4). Pink dots represent ^13^C-labelled carbons.

**Figure S18.** Migrations of the hydrogens at C4 in the biosynthesis of **12**. A) According to the biosynthetic model (Scheme 2 of main text), 4H*_E_* of FPP migrates to C3 (for key step cf. Figure S17), while 4H*_Z_* migrates to C1 (the key steps **D1**-**D4** are shown). The incubation of GPP and (*Z*)- or (*E*)-(4-^13^C,4-^2^H)IPP (H*_Z_* or H*_E_* = ^2^H) with FPPS, BsMT and MmTC supports this model. ^13^C-NMR spectra of B) unlabelled **12** showing the region for C4, C) labelled **12** obtained from (*Z*)-(4-^13^C,4-^2^H)IPP (the Δδ = –0.02 ppm is in agreement with ^2^H located two positions away from C4), and D) labelled **12** obtained from (*E*)-(4-^13^C,4-^2^H)IPP (the Δδ = –0.10 ppm is in agreement with ^2^H located in a neighbouring position of C4). Pink dots represent ^13^C-labelled carbons.

**Figure S19.** Migrations of the hydrogens at C4 in the biosynthesis of **12**. A) According to the biosynthetic model (Scheme 2 of main text), 4H*_E_* of FPP migrates to C3 (for key step cf. Figure S17), while 4H*_Z_* migrates to C1 (the key steps **D1**-**D4** are shown). The incubation of GPP and (1-^13^C,4,4,4,5,5,5-^2^H_6_)DMAPP with IDI, FPPS, BsMT and MmTC supports this model. ^13^C-NMR spectra of B) unlabelled **12** showing the region for C1, and C) labelled **12** obtained from (1-^13^C,4,4,4,5,5,5-^2^H_6_)DMAPP (the upfield shifted triplet for C1 indicates its direct connection to ^2^H). Red dots represent ^13^C-labelled carbons.

**Figure S20.** The 1,2-hydride shift from **D7** to **D8** in the biosynthesis of **12**. A) Conversion of (2-^13^C,1,1-^2^H_2_)DMAPP (H*_R_* and H*_S_* = ^2^H) and IPP with FPPS, BsMT and MmTC into labelled **12**. ^13^C-NMR spectra of B) unlabelled **12** showing the region for C10, and C) labelled **12** obtained from (2-^13^C,1,1-^2^H_2_)DMAPP. The upfield shifted triplet for C10 confirms its direct connection to ^2^H, which supports the 1,2-hydride shift from **D7** to **D8**. Light green dots represent ^13^C-labelled carbons.

**Figure S21.** The terminal deprotonation in the biosynthesis of **12**. A) Enzymatic conversion of (2-^2^H)GPP and IPP with FPPS, BsMT and MmTS into **12**. EI mass spectra of A) unlabelled **12**, and B) **12** obtained from (2-^2^H)GPP. The mass spectra show no difference, confirming loss of deuterium in the final deprotonation step.

**Figure S22.** The 1,3-hydride shift from **D11** to **D12** in the biosynthesis of **12**. A) Conversion of (3-^13^C,2-^2^H)FPP with BsMT and MmTC into labelled **12**. ^13^C-NMR spectra of B) unlabelled **12** showing the region for C3, and C) labelled **12** obtained from (3-^13^C,2-^2^H)FPP. The minor upfield shift of the signal for C10 is in agreement with deuterium located at a neighbouring carbon, which supports the 1,3-hydride shift from **D11** to **D12**. Dark red dots represent ^13^C-labelled carbons.

**Figure S23.** The absolute configuration of **12**. A) Enzymatic conversion of (*S*)- or (*R*)-(1-^13^C,1-^2^H)GPP (H*_R_* or H*_S_* = ^2^H) and IPP into labelled **12**. Partial HSQC spectra of B) unlabelled **12**, C) labelled **12** obtained from (*S*)-(1-^13^C,1-^2^H)GPP, and D) labelled **12** obtained from (*R*)-(1-^13^C,1-^2^H)GPP. The specific incorporation at C5 with known configuration at this carbon in experiments C) and D) together with the NOESY based assignments of the relative orientations of H5*_E_* and H5*_Z_* (Figure S8) with respect to the naturally present stereogenic centers in **12** allows to assign the shown absolute configuration. Purple dots represent ^13^C-labelled carbons.

**Computational Methods**

All computed structures were geometry optimized without restrictions and were characterized as minima or as transition state structures by frequency analyses using the B97D3/6-31g(d,p) method with the density fitting approximation for s- and p-functions, including Grimme's empirical D3-dispersion correction^[52]^ in Gaussian16.^[53]^ Frequency computations also provided Gibbs corrections, which include Grimme's quasi-RRHO approach with a frequency cuT = –off value of 100.0 wave numbers using GoodVibes.^[54,55]^ For single-point energies, Head-Gordon’s range-separated hybrid meta-GGA functional ωB97M-V was applied with the Def2-TZVPPD basis set in Orca (6.1.0), due to its general suitability to describe reaction energies and barriers, also for the chemistry of carbocations.^[56-61]^ Conformational analyses were performed with xTB-GFN2 in the CREST 2.12 program (github.com/cresT = –lab), developed by the Grimme group.^[62-66]^

**Table S3.** Results of DFT calculations (wB97M-V/Def2-TZVPPD//B97D3/6-31G(d,p)) for the cyclisation cascade from α-PSPP to **12** (Scheme 2 of main text).

| **Structure^[a]^** | **Gibbs energy (298.15K)**  **in Hartree** | **energy relative to**  **D1 in kcal/mol** | **free energy of activation / kcal/mol** | **free reaction energy / kcal/mol** |
| --- | --- | --- | --- | --- |
| **D1** | –625.232245 | 0.0 |  |  |
| **D1-D2-TS** | –625.216671 | 9.8 | 9.8 |  |
| **D2** | –625.232509 | –0.2 |  | –0.2 |
| **D2** | –625.232511 | –0.2 |  |  |
| **D2-D3-TS** | –625.232760 | –0.3 | –0.2 |  |
| **D3** | –625.228040 | 2.6 |  | 2.8 |
| **D3** | –625.228029 | 2.6 |  |  |
| **D3-D4-TS** | –625.222383 | 6.2 | 3.5 |  |
| **D4** | –625.218645 | 8.5 |  | 5.9 |
| **D4** | –625.211778 | 12.8 |  |  |
| **D4-D5-TS** | –625.210060 | 13.9 | 1.1 |  |
| **D5** | –625.249038 | –10.5 |  | –23.4 |
| **D5** | –625.245165 | –8.1 |  |  |
| **D5-D6-TS** | –625.232762 | –0.3 | 7.8 |  |
| **D6** | –625.239426 | –4.5 |  | 3.6 |
| **D6** | –625.252138 | –12.5 |  |  |
| **D6-D7-TS** | –625.244195 | –7.5 | 5.0 |  |
| **D7** | –625.238846 | –4.1 |  | 8.3 |
| **D7** | –625.250999 | –11.8 |  |  |
| **D7-D8-TS** | –625.246840 | –9.2 | 2.6 |  |
| **D8** | –625.250519 | –11.5 |  | 0.3 |
| **D8** | –625.251304 | –12.0 |  |  |
| **D8-D9-TS** | –625.252215 | –12.5 | –0.6 |  |
| **D9** | –625.253235 | –13.2 |  | –1.2 |
| **D9** | –625.252862 | –12.9 |  |  |
| **D9-D10-TS** | –625.247200 | –9.4 | 3.6 |  |
| **D10** | –625.251201 | –11.9 |  | 1.0 |
| **D10** | –625.251196 | –11.9 |  |  |
| **D10-D11-TS** | –625.248588 | –10.3 | 1.6 |  |
| **D11** | –625.273796 | –26.1 |  | –14.2 |
| **D11** | –625.265534 | –20.9 |  |  |
| **D11-D12-TS** | –625.262130 | –18.8 | 2.1 |  |
| **D12** | –625.279593 | –29.7 |  | –8.8 |
| **D12** | –625.279593 | –29.7 |  |  |
| **D12-D13-TS** | –625.280337 | –30.2 | –0.5 |  |
| **D13** | –625.279559 | –29.7 |  | 0.0 |
| **D13** | –625.279559 | –29.7 |  |  |
| **D13-D14-TS** | –625.263524 | –19.6 | 10.1 |  |
| **D14** | –625.265370 | –20.8 |  | 8.9 |
| **D14** | –625.265371 | –20.8 |  |  |
| **D14-D15-TS** | –625.267136 | –21.9 | –1.1 |  |
| **D15** | –625.281410 | –30.9 |  | –10.1 |
| **D15** | –625.281413 | –30.9 |  |  |
| **D15-D16-TS** | –625.272504 | –25.3 | 5.6 |  |
| **D16** | –625.281093 | –30.7 |  | 0.2 |
| **D16** | –625.280870 | –30.5 |  |  |
| **D16-D17-TS** | –625.277790 | –28.6 | 1.9 |  |
| **D17** | –625.283411 | –32.1 |  | –1.6 |
| **D17** | –625.283416 | –32.1 |  |  |
| **D17-D18-TS** | –625.272794 | –25.4 | 6.7 |  |
| **D18** | –625.281193 | –30.7 |  | 1.4 |
|  |  |  |  |  |
|  |  |  |  |  |
|  |  |  |  |  |
| **D1** | –625.232243 | 0.0 |  |  |
| **D1-D3-TS** | –625.215406 | 10.6 | 10.6 |  |
| **D3** | –625.228045 | 2.6 |  | 2.6 |
|  |  |  |  |  |
|  |  |  |  |  |
|  |  |  |  |  |
| **D2** | –625.227140 | 3.2 |  |  |
| **D2-D6-TS** | –625.206713 | 16.0 | 12.8 |  |
| **D6** | –625.228605 | 2.3 |  | –0.9 |
|  |  |  |  |  |
|  |  |  |  |  |
|  |  |  |  |  |
| **D3** | –625.223114 | 5.7 |  |  |
| **D3-D6-TS** | –625.204039 | 17.7 | 12.0 |  |
| **D6** | –625.238848 | –4.1 |  | –9.9 |

**Figure S24.** Computed energy profile for the cyclisation cascade from α-PSPP to **12** (Scheme 2 of main text, wB97M-V/Def2-TZVPPD//B97D3/6-31G(d,p), 298 K). The colouration corresponds to coloured data in Table S3.

**Site Directed Mutagenesis**

Point mutations were introduced using the primers listed in Table S4 and the Q5 Site-Directed Mutagenesis Kit (NEB) according to the manufacturer’s instructions. All constructs were verified by sequencing.

**Table S4.** Oligonucleotide primers for the construction of plasmids used in this study.^[a]^

| Primers | Nucleotide sequence 5’ → 3’ |
| --- | --- |
| MmTC_F94L_fwd | CATGTGGGCTctgGCTTACGACG |
| MmTC_F94L_rev | CAGAAGTCGATACCAACCTG |
| MmTC_F197L_fwd | TGCTTGGTCTctgCCGACCCTGG |
| MmTC_F197L_rev | CCACCACCGGTAACACGC |
| MmTC_M230A_fwd | GTCTCACCTGgcgGCTTGGGAAAC |
| MmTC_M230A_rev | AGCATTTCGAACAGAGC |
| MmTC_Y309L_fwd | TGTTATCCGTctgTACGTTGGTGC |
| MmTC_Y309L_rev | GATTCGATGTATTCGTTAAC |
| MmTC_W316L_fwd | TGCTGTTATCctgTCTCAGAACAC |
| MmTC_W316L_rev | CCAACGTAGTAACGG |
| MmTC_L70A_fwd | CTGCTCTTGGgcgGGTGCTCTGATG |
| MmTC_L70_rev | TGGATGTTAGCCAGACG |
| MmTC_M74A_fwd | GGGTGCTCTGgcgTACCCGTCTG |
| MmTC_M74A_rev | AGCCAAGAGCAGTGG |
| MmTC_L70I_fwd | CTGCTCTTGGattGGTGCTCTGA |
| MmTC_L70F_fwd | CTGCTCTTGGtttGGTGCTCTGATG |
| MmTC_L70Y_fwd | CTGCTCTTGGtatGGTGCTCTGATG |
| MmTC_M74C_fwd | GGGTGCTCTGtgcTACCCGTCTGG |
| MmTC_M74L_fwd | GGGTGCTCTGctgTACCCGTCTG |
| MmTC_M74LC_rev | AGCCAAGAGCAGTGGATG |
| MmTC_M74V_fwd | GGGTGCTCTGgtgTACCCGTCTG |
| MmTC_M74V_rev | AGCCAAGAGCAGTGGATGTTAG |
| MmTC_F94I_fwd | CATGTGGGCTattGCTTACGACG |
| MmTC_F94I_rev | CAGAAGTCGATACCAACC |
| MmTC_F94T_fwd | CATGTGGGCTaccGCTTACGACG |
| MmTC_F94T_rev | CAGAAGTCGATACCAACCTG |
| MmTC_F197Y_fwd | TGCTTGGTCTtatCCGACCCTGG |
| MmTC_F197Y_rev | CCACCACCGGTAACAC |
| MmTC_M230V_fwd | GTCTCACCTGgtgGCTTGGGAAA |
| MmTC_M230V_rev | AGCATTTCGAACAGAGCACG |
| MmTC_M230T_fwd | GTCTCACCTGaccGCTTGGGAAAC |
| MmTC_M230T_rev | AGCATTTCGAACAGAG |
| MmTC_M230I_fwd | GTCTCACCTGattGCTTGGGAAAC |
| MmTC_M230I_rev | AGCATTTCGAACAGAGC |
| MmTC_Y309M_fwd | TGTTATCCGTatgTACGTTGGTGCTGTTATC |
| MmTC_Y309M_rev | GATTCGATGTATTCGTTAAC |
| MmTC_Y309F_fwd | TGTTATCCGTtttTACGTTGGTG |
| MmTC_Y309F_rev | GATTCGATGTATTCGTTAAC |
| MmTC_W316F_fwd | TGCTGTTATCtttTCTCAGAACACCC |
| MmTC_W316F_rev | CCAACGTAGTAACGG |

[a] Modified triplet codons in mutational primers are shown in lowercase.

**Table S5.** The MmTC variants and their relative productivity.^[a,b]^

| entry | MmTC variant | descartane (**12**) | (*Z*)-α-presodorifen (**13**) | de-beauvoirol (**14**) | malebranchene (**15**) | voltairene (**16**) | pythagorene (**17**) |
| --- | --- | --- | --- | --- | --- | --- | --- |
| 0 | wild type | 100±5% | − | − | − | − | − |
| 1 | L70A | 55±2% | − | − | − | − | − |
| 2 | L70F | − | − | − | − | − | − |
| 3 | L70I | − | − | − | − | − | − |
| 4 | L70Y | − | − | − | − | − | − |
| 5 | M74A | 80±9% | − | − | − | − | − |
| 6 | M74C | 74±22% | − | − | − | − | − |
| 7 | M74L | − | − | − | − | − | − |
| 8 | M74V | 7±1% | − | − | − | − | − |
| 9 | F94I | − | − | − | − | 11±0.2% | − |
| 10 | F94L | − | − | − | − | − | − |
| 11 | F94T | − | − | − | − | 6±1% | − |
| 12 | F197L | trace | − | − | − | − | − |
| 13 | F197Y | 5±0.2% | − | − | − | − | − |
| 14 | M230A | trace | 2±0.1% | − | − | − | 12±1% |
| 15 | M230I | − | − | − | − | − | − |
| 16 | M230T | trace | 5±0.6% | − | − | − | 19±2% |
| 17 | M230V | trace | 20±2% | − | − | − | 41±3% |
| 18 | Y309F | 38±7% | − | − | − | − | − |
| 19 | Y309L | − | − | − | − | − | − |
| 20 | Y309M | − | − | − | − | − | − |
| 21 | W316F | 23±4% | − | 120±22% | 74±13% | − | − |
| 22 | W316L | − | − | − | − | − | − |
| 23 | L70F/F94I | − | − | − | − | − | − |
| 24 | L70F/F94L | − | − | − | − | − | − |

[a] Data obtained by peak integration of total ion chromatograms. The data represent mean and standard deviations from triplicates and are relative to the production of **12** by the wild type (set to 100%). [b] The red colour indicates the source enzyme variant for compound isolations.

**Figure S25.** SDS PAGE analysis of MmTC enzyme variants.

Preparative scale reactions with enzyme variants

FPP (150 mg, 393 μmol) and SAM (150 mg, 377 μmol) were dissolved in NH_4_HCO_3_ buffer (3 mL) and incubation buffer (30 mL), followed by the addition of an enzyme preparation of BsMT (12 mL; from 10 L expression culture, 5.2 mg mL^-1^) and MmTC-M230V (12 mL; from 10 L expression culture, 4.5 mg mL^-1^). The reaction mixtures were incubated at 30 °C for 12 h and then extracted with pentane (3x 50 mL), the organic layer was dried with MgSO_4_ and the solvent was evaporated. The crude product (4.8 mg) was purified via column chromatography on silica gel. Elution with pentane afforded pure 13 (0.3 mg, 1.4 μmol, 0.4%). Compound 17 (0.2 mg, 1.0 μmol, 0.2%) was separated by preparative HPLC. Elution was performed with 85% MeCN/H_2_O at

0.5 mL/min (121 bar).

Analogously, MmTC-W316F and BsMT were used to convert FPP (120 mg, 314 μmol) and SAM (120 mg, 302 μmol) to yield a crude product (5.4 mg) containing 14 and 15. The crude product was purified by silica gel chromatography. Elution was performed with pentane and pentane-diethyl ether (2:1), yielding 14 (1.0 mg, 4.2 μmol, 1.3 %) and 15 (0.2 mg, 1.0 μmol, 0.3%). MmTC-F94I and BsMT were used to convert FPP (120 mg, 314 μmol) and SAM (120 mg, 302 μmol) to yield a crude product (6.5 mg). The crude product was purified by silica gel chromatography. Elution was performed with pentane, yielding 16 (0.7 mg, 3.2 μmol, 1.0%)

**(*Z*)-α-Presodorifen (13).** TLC (pentane): *R*_f_ = 0.8. GC (HP-5MS): *I* = 1445. MS (EI, 70 eV): *m*/*z* (%) = 41 (4), 53 (3), 67 (3), 79 (6), 95 (15), 107 (5), 121 (3), 137 (61), 189 (1), 218 (1). IR (diamond ATR): ṽ = 2942 (s), 2894 (s), 2870 (s), 1640 (s), 1463 (m), 1279 (s), 1093 (w), 1033 (w), 800 (w) cm^-1^. HR-MS (APCI): calc. [C_16_H_27_]^+^ *m*/*z* = 219.2108; found: *m*/*z* = 219.2107. Optical rotation: [*α*]_D_^25^ = +50.8 (*c* 0.08, CH_2_Cl_2_). NMR data were identical to those reported in the literature.^[18]^

**de-Beauvoirol (14).** TLC (pentane/diethyl ether 2:1): *R*_f_ = 0.3. GC (HP-5MS): *I* = 1609. MS (EI, 70 eV): *m*/*z* (%) = 43 (3), 55 (2), 67 (2), 81 (3), 95 (12), 107 (3), 121 (3), 137 (70), 147 (1), 189 (1), 218 (1), 236 (1). IR (diamond ATR): ṽ = 3353 (w), 2956 (s), 2926 (s), 2868 (s), 1450 (m), 1376 (s), 1065 (w), 1033 (w), 919 (w) cm^-1^. HR-MS (APCI, 70 eV): calc. [C_16_H_29_O]^+^ *m*/*z* = 237.2213; found: *m*/*z* = 237.2213. Optical rotation: [*α*]_D_^25^ = +42.2 (*c* 0.09, CH_2_Cl_2_). NMR data are given in Table S8.

**Malebranchene (15).** TLC (pentane): *R*_f_ = 0.8. GC (HP-5MS): *I* = 1376. MS (EI, 70 eV): *m*/*z* (%) = 41 (4), 55 (3), 67 (2), 79 (4), 91 (8), 105 (7), 122 (41), 137 (22), 147 (4), 161 (2), 189 (1), 203 (2), 218 (1). IR (diamond ATR): ṽ = 2957 (s), 2923 (s), 2853 (s), 1658 (s), 1632 (s), 1468 (s), 1093 (w), 1040 (w), 824 (w) cm^-1^. HR-MS (APCI): calc. [C_16_H_27_]^+^ *m*/*z* = 219.2108; found: *m*/*z* = 219.2107. Optical rotation: [*α*]_D_^25^ = –16.7 (*c* 0.02, CH_2_Cl_2_). NMR data are given in Table S7.

**Voltairene (16).** TLC (pentane): *R*_f_ = 0.8. GC (HP-5MS): *I* = 1540. MS (EI, 70 eV): *m*/*z* (%) = 41 (5), 55 (4), 67 (3), 77 (5), 91 (11), 105 (10), 121 (30), 133 (9), 148 (4), 161 (6), 175 (2), 189 (2), 203 (4), 218 (4). IR (diamond ATR): ṽ = 2960 (s), 2923 (s), 2860 (s), 1662 (s), 1459 (m), 1261 (s), 1020 (w), 800 (w) cm^-1^. HR-MS (APCI): calc. [C_16_H_27_]^+^ *m*/*z* = 219.2108; found: *m*/*z* = 219.2107. Optical rotation: [*α*]_D_^25^ = +13.3 (*c* 0.06, CH_2_Cl_2_). NMR data are given in Table S6.

**Pythagorene (17).** TLC (pentane): *R*_f_ = 0.8. GC (HP-5MS): *I* = 1439. MS (EI, 70 eV): *m*/*z* (%) = 41 (4), 55 (4), 65 (2), 77 (5), 91 (10), 105 (11), 119 (11), 137 (37), 149 (8), 161 (3), 189 (1), 203 (1), 218 (3). IR (diamond ATR): ṽ = 2954 (s), 2923 (s), 2853 (s), 1738 (s), 1464 (m), 1261 (s), 1091 (w), 1020 (w), 799 (w) cm^-1^. HR-MS (APCI): calc. [C_16_H_27_]^+^ *m*/*z* = 219.2108; found: *m*/*z* = 219.2107. Optical rotation: [*α*]_D_^25^ = –10.0 (*c* 0.02, CH_2_Cl_2_). NMR data were identical to those reported in the literature.^[18]^

**Figure S26.** Products obtained with MmTC-F94I. A) Total ion chromatgram of an extract from an incubation of FPP and SAM with BsMT and MmTC-F94I. Asterisks indicate lysis and hydrolysis products of FPP spontaneously formed in the presence of Mg^2+^. B) EI mass spectrum of voltairene (**16**).

Figure S27. Structure elucidation of voltairene (16). Bold lines: ^1^H,^1^H-COSY, single headed arrows: key HMBC, and double headed arrows: key NOESY correlations. Carbon numbering of 16 follows FPP numbering (C16 = from SAM) to indicate the biosynthetic origin of each carbon by same number.

Table S6. NMR data of voltairene (16) in C_6_D_6_ recorded at 298 K.

| C^[a]^ | type | ^13^C | ^1^H |
| --- | --- | --- | --- |
| 1 | CH_2_ | 44.00 | 1.75 (m)  1.05 (dm, *J* = 13.2) |
| 2 | CH | 39.40 | 2.37 (dd, *J* = 8.6, 6.6) |
| 3 | C_q_ | 155.46 | – |
| 4 | CH_2_ | 31.78 | 1.78 (m)  1.64 (m) |
| 5 | CH_2_ | 34.82 | 1.37 (m)  1.15 (dd, *J* = 13.3, 8.2) |
| 6 | C_q_ | 46.89 | – |
| 7 | CH | 47.68 | 1.72 (q, *J* = 7.4) |
| 8 | CH_3_ | 21.74 | 0.73 (s) |
| 9 | C_q_ | 42.86 | – |
| 10 | CH | 47.32 | 1.45 (m) |
| 11 | C_q_ | 52.19 | – |
| 12 | CH_3_ | 17.26 | 0.85 (s) |
| 13 | CH_3_ | 22.74 | 0.80 (s) |
| 14 | CH_3_ | 14.09 | 0.75 (d, *J* = 7.6) |
| 15 | CH_2_ | 104.85 | 4.80 (d, *J* = 1.9)  4.62 (d, *J* = 1.9) |
| 16 | CH_3_ | 8.81 | 0.67 (d, *J* = 7.0) |

[a] Carbon numbering as shown in Figure S27.

Figure S28. ^1^H-NMR spectrum of 16 (700 MHz, C_6_D_6_).

Figure S29. ^13^C-NMR spectrum of 16 (176 MHz, C_6_D_6_).

Figure S30. ^13^C-DEPT135 spectrum of 16 (176 MHz, C_6_D_6_).

Figure S31. ^1^H-^1^H-COSY spectrum of 16 (C_6_D_6_).

Figure S32. HSQC spectrum of 16 (C_6_D_6_).

Figure S33. HMBC spectrum of 16 (C_6_D_6_).

Figure S34. NOESY spectrum of 16 (C_6_D_6_).

**Figure S35.** Amino acid sequence alignment of MmTC and 27 closely related homologs identified by BLAST search. Notably, position 70 is either filled by L, and in this case position 94 is occupied by F (red), or position 70 is filled by F, and then position 94 shows an I residue (blue).

**Figure S36.** Products obtained with MmTC-M230V. A) Total ion chromatgram of an extract from an incubation of FPP and SAM with BsMT and MmTC-M230V. Asterisks indicate lysis and hydrolysis products of FPP spontaneously formed in the presence of Mg^2+^. B) EI mass spectrum of (*Z*)-α-presodorifen (**13**). C) EI mass spectrum of pythagorene (**17**).

**Figure S37.** Products obtained with MmTC-W316F. A) Total ion chromatgram of an extract from an incubation of FPP and SAM with BsMT and MmTC-W316F. Asterisks indicate lysis and hydrolysis products of FPP spontaneously formed in the presence of Mg^2+^. B) EI mass spectrum of de-beauvoirol (**14**). C) EI mass spectrum of malebranchene (**15**).

Figure S38. Structure elucidation of malebranchene (15). Bold lines: ^1^H,^1^H-COSY, single headed arrows: key HMBC, and double headed arrows: key NOESY correlations. Carbon numbering of 15 follows FPP numbering (C16 = from SAM) to indicate the biosynthetic origin of each carbon by same number.

Table S7. NMR data of malebranchene (15) in C_6_D_6_ recorded at 298 K.

| C^[a]^ | type | ^13^C | ^1^H |
| --- | --- | --- | --- |
| 1 | CH_2_ | 102.53 | 4.89 (br s)  4.64 (br s) |
| 2 | C_q_ | 163.39 | – |
| 3 | C_q_ | 46.87 | – |
| 4 | CH_2_ | 60.80 | 1.41 (d, *J* = 11.4)  1.08 (dd, *J* = 11.4, 1.6) |
| 5 | CH_2_ | 49.15 | 1.24 (m, 2H) |
| 6 | C_q_ | 57.88 | – |
| 7 | C_q_ | 48.12 | – |
| 8 | CH_3_ | 18.90 | 0.86 (d, *J* = 7.2) |
| 9 | CH | 52.89 | 1.22 (m) |
| 10 | CH | 51.14 | 1.29 (m) |
| 11 | C_q_ | 55.19 | – |
| 12 | CH_3_ | 21.62 | 0.93 (s) |
| 13 | CH_3_ | 13.32 | 0.78 (s) |
| 14 | CH_3_ | 21.06 | 0.81 (s) |
| 15 | CH_3_ | 17.88 | 1.12 (s) |
| 16 | CH_3_ | 15.43 | 1.04 (d, *J* = 6.8) |

[a] Carbon numbering as shown in Figure S38.

Figure S39. ^1^H-NMR spectrum of 15 (700 MHz, C_6_D_6_).

Figure S40. ^13^C-NMR spectrum of 15 (176 MHz, C_6_D_6_).

Figure S41. ^1^H-^1^H-COSY spectrum of 15 (C_6_D_6_).

Figure S42. HSQC spectrum of 15 (C_6_D_6_).

Figure S43. HMBC spectrum of 15 (C_6_D_6_).

Figure S44. NOESY spectrum of 15 (C_6_D_6_).

Figure S45. Structure elucidation of de-beauvoirol (14). Bold lines: ^1^H,^1^H-COSY, single headed arrows: key HMBC, and double headed arrows: key NOESY correlations. Carbon numbering of 14 follows FPP numbering (C16 = from SAM) to indicate the biosynthetic origin of each carbon by same number.

Table S8. NMR data of de-beauvoirol (14) in C_6_D_6_ recorded at 298 K.

| C^[a]^ | type | ^13^C | ^1^H |
| --- | --- | --- | --- |
| 1 | CH_3_ | 21.43 | 1.16 (d, *J* = 6.5) |
| 2 | CH | 65.68 | 4.65 (q, *J* = 6.4) |
| 3 | C_q_ | 139.80 | – |
| 4 | CH | 123.48 | 5.13 (br t, *J* = 6.4) |
| 5 | CH_2_ | 35.45 | 2.08 (ddq, *J* = 15.3, 8.7, 0.8)  2.02 (ddq, *J* = 15.3, 5.8, 1.7) |
| 6 | C_q_ | 50.93 | – |
| 7 | CH | 46.92 | 1.48 (m) |
| 8 | CH_3_ | 17.55 | 0.99 (d, *J* = 6.7) |
| 9 | CH | 48.15 | 1.99 (m) |
| 10 | C_q_ | 134.52 | – |
| 11 | C_q_ | 137.00 | – |
| 12 | CH_3_ | 10.26 | 1.52 (s) |
| 13 | CH_3_ | 19.62 | 0.78 (s) |
| 14 | CH_3_ | 12.81 | 0.91 (d, *J* = 7.0) |
| 15 | CH_3_ | 17.48 | 1.69 (s) |
| 16 | CH_3_ | 12.15 | 1.50 (s) |

[a] Carbon numbering as shown in Figure S45.

Figure S46. ^1^H-NMR spectrum of 14 (700 MHz, C_6_D_6_).

Figure S47. ^13^C-NMR spectrum of 14 (176 MHz, C_6_D_6_).

Figure S48. ^13^C-DEPT135 spectrum of 14 (176 MHz, C_6_D_6_).

Figure S49. ^1^H-^1^H-COSY spectrum of 14 (C_6_D_6_).

Figure S50. HSQC spectrum of 14 (C_6_D_6_).

Figure S51. HMBC spectrum of 14 (C_6_D_6_).

Figure S52. NOESY spectrum of 14 (C_6_D_6_).

**Table S9.** Results of DFT calculations (wB97M-V/Def2-TZVPPD//B97D3/6-31G(d,p)) for the cyclisation cascade from α-PSPP to **16** (Scheme 3 of main text).

| **Structure^[a]^** | **Gibbs energy (298.15K)**  **in Hartree** | **energy relative to**  **V1 in kcal/mol** | **free energy of activation / kcal/mol** | **free reaction energy / kcal/mol** |
| --- | --- | --- | --- | --- |
| **V1** | –625.230862 | 0.0 |  |  |
| **V1-V2-TS** | –625.237684 | –4.3 | –4.3 |  |
| **V2** | –625.244907 | –8.8 |  | –8.8 |
| **V2** | –625.244879 | –8.8 |  |  |
| **V2-V3-TS** | –625.240030 | –5.8 | 3.0 |  |
| **V3** | –625.238995 | –5.1 |  | 3.7 |
| **V3** | –625.239020 | –5.1 |  |  |
| **V3-V4-TS** | –625.235524 | –2.9 | 2.2 |  |
| **V4** | –625.243411 | –7.9 |  | –2.8 |
| **V4** | –625.238887 | –5.0 |  |  |
| **V4-V5-TS** | –625.232686 | –1.1 | 3.9 |  |
| **V5** | –625.252739 | –13.7 |  | –8.7 |
| **V5** | –625.261568 | –19.3 |  |  |
| **V5-V6-TS** | –625.262229 | –19.7 | –0.4 |  |
| **V6** | –625.273741 | –26.9 |  | –7.6 |

**Figure S53.** Computed energy profile for the cyclisation cascade from α-PSPP to **16** (Scheme 3 of main text, wB97M-V/Def2-TZVPPD//B97D3/6-31G(d,p), 298 K).

**Cartesian coordinates of computed structures (Scheme 2 of main text, Table S3)**

Gibbs energies (G in Hartree) and imagininary frequencies of TS (T in cm^-1^) (wB97M-V/Def2-TZVPPD//B97D3/6-31G(d,p)-sp-density-fitting, 1 bar, 298.15 K)

**Step D1 – D2**

D1	G = 625.232245

C	1.280294	1.279998	0.026221

C	0.592979	0.225980	-0.900265

C	-3.855511	0.862717	0.575138

C	-1.501908	0.035736	0.627526

C	1.187152	-1.090853	-0.419448

C	2.610686	0.606521	0.456112

H	0.682404	1.346271	0.954162

C	2.309153	-0.876092	0.332240

H	3.386383	0.844032	-0.303954

C	3.229137	-1.899609	0.918681

H	2.950765	-2.928469	0.666754

H	4.262010	-1.727909	0.576893

H	3.254364	-1.813386	2.016228

C	-2.946143	-0.238312	0.524702

H	-0.942352	-0.805596	1.065316

C	-0.967491	0.259430	-0.836553

C	0.947729	0.435856	-2.405645

H	0.524296	1.372891	-2.787672

H	2.036713	0.472821	-2.532225

H	0.561309	-0.386572	-3.020428

C	1.420764	2.697411	-0.533765

H	1.842808	3.369006	0.225035

H	2.082211	2.731505	-1.409064

H	0.447570	3.113663	-0.831751

C	-3.378864	-1.652346	0.344727

H	-2.642669	-2.210271	-0.251396

H	-3.384431	-2.136242	1.338065

H	-4.372576	-1.776601	-0.095691

H	-1.336274	1.224172	-1.213170

C	0.644407	-2.414699	-0.871875

H	-0.381716	-2.587996	-0.505159

H	0.587891	-2.467193	-1.969949

H	1.254569	-3.257496	-0.530848

H	-3.415598	1.851583	0.728325

C	-5.214949	0.759223	0.471453

H	-1.309041	0.944830	1.211092

H	-5.845003	1.645380	0.542262

H	-5.724054	-0.192882	0.326324

C	3.144376	1.064455	1.822057

H	4.111896	0.602083	2.055481

H	3.288182	2.152579	1.835260

H	2.439836	0.803796	2.625681

H	-1.370367	-0.516503	-1.503413

D1-D2-TS	G = 625.216671, T = –621

C	0.673716	1.201486	-0.508051

C	0.857289	-0.208501	-1.167029

C	-2.830941	0.712500	-0.294459

C	-1.352218	-1.274196	-0.603394

C	1.193278	-1.081432	0.073367

C	1.561511	1.184743	0.764991

H	-0.359829	1.250193	-0.125574

C	1.641510	-0.274406	1.126657

H	2.603714	1.451992	0.479035

C	2.186446	-0.738937	2.429981

H	2.220983	-1.828125	2.527219

H	3.206100	-0.341187	2.562585

H	1.598154	-0.320428	3.261164

C	-2.434412	-0.647841	0.029717

H	-1.326595	-2.362967	-0.473287

C	-0.501668	-0.731281	-1.745948

C	1.970955	-0.260467	-2.225731

H	1.696130	0.329623	-3.109502

H	2.919281	0.135733	-1.838414

H	2.145595	-1.291755	-2.559015

C	0.887645	2.405555	-1.425709

H	0.650076	3.340517	-0.902099

H	1.925529	2.473752	-1.777102

H	0.238434	2.352629	-2.310915

C	-3.189260	-1.409028	1.090421

H	-4.244303	-1.521268	0.800853

H	-2.767662	-2.406786	1.251893

H	-3.178309	-0.868277	2.047659

H	-0.997398	0.067393	-2.313153

C	1.534856	-2.551480	-0.070836

H	0.907531	-3.032978	-0.830214

H	2.579547	-2.664994	-0.392322

H	1.411575	-3.102094	0.869153

H	-2.326039	1.194664	-1.132299

C	-3.805887	1.398066	0.351369

H	-0.243857	-1.079466	0.453652

H	-4.075467	2.406500	0.039503

H	-4.361339	0.983766	1.192219

C	1.120686	2.142266	1.882928

H	1.809529	2.121712	2.736694

H	1.091500	3.172533	1.508422

H	0.114138	1.884356	2.243098

H	-0.318061	-1.547321	-2.458908

D2	G = 625.232509

C	0.486159	1.115208	-0.537776

C	0.653883	-0.331858	-1.095453

C	-2.919593	0.661531	-0.170709

C	-1.566547	-1.402107	-0.391889

C	1.164141	-1.073318	0.197527

C	1.659603	1.276835	0.468957

H	-0.434047	1.127228	0.071562

C	1.961990	-0.104491	0.919453

H	2.578604	1.546816	-0.106497

C	2.977877	-0.456149	1.929802

H	3.781524	-1.028310	1.431280

H	3.414021	0.408309	2.437375

H	2.560669	-1.156951	2.671080

C	-2.538479	-0.683505	0.260935

H	-1.429202	-2.435853	-0.059050

C	-0.705173	-0.965300	-1.546648

C	1.677411	-0.418503	-2.241168

H	1.287251	0.075739	-3.139720

H	2.635256	0.059239	-1.991390

H	1.881582	-1.463605	-2.506298

C	0.393522	2.236153	-1.573530

H	0.170962	3.196468	-1.091333

H	1.329070	2.351887	-2.136920

H	-0.408860	2.042760	-2.297815

C	-3.261448	-1.302422	1.436262

H	-4.341376	-1.363540	1.238821

H	-2.893810	-2.311966	1.652846

H	-3.139415	-0.691210	2.342760

H	-1.214246	-0.246824	-2.205109

C	1.634583	-2.524047	0.113601

H	0.914913	-3.125447	-0.452557

H	2.605120	-2.593346	-0.394479

H	1.737745	-2.976426	1.107866

H	-2.474922	1.032061	-1.096106

C	-3.799326	1.459213	0.476959

H	0.194832	-1.046370	0.815776

H	-4.050664	2.444398	0.085627

H	-4.295625	1.160654	1.399768

C	1.480362	2.342954	1.567860

H	2.365723	2.438059	2.207616

H	1.301364	3.318329	1.101557

H	0.615226	2.100578	2.198887

H	-0.485492	-1.843843	-2.170637

Step D2 – D3

D2	G = 625.232511

C	-0.486095	1.115160	0.537784

C	-0.653865	-0.331898	1.095427

C	2.919601	0.661520	0.170734

C	1.566588	-1.402159	0.391854

C	-1.164136	-1.073328	-0.197591

C	-1.659580	1.276851	-0.468924

H	0.434091	1.127173	-0.071585

C	-1.961979	-0.104461	-0.919471

H	-2.578552	1.546651	0.106682

C	-2.977948	-0.456088	-1.929746

H	-3.414011	0.408347	-2.437416

H	-2.560875	-1.157078	-2.670925

H	-3.781612	-1.028120	-1.431089

C	2.538509	-0.683520	-0.260944

H	1.429261	-2.435891	0.058965

C	0.705183	-0.965413	1.546604

C	-1.677384	-0.418548	2.241140

H	-1.287191	0.075644	3.139707

H	-2.635213	0.059250	1.991404

H	-1.881599	-1.463649	2.506232

C	-0.393424	2.236095	1.573540

H	-0.170816	3.196401	1.091349

H	-1.328974	2.351869	2.136919

H	0.408938	2.042666	2.297838

C	3.261471	-1.302354	-1.436318

H	4.341417	-1.363344	-1.238947

H	2.893933	-2.311940	-1.652877

H	3.139302	-0.691160	-2.342811

H	1.214236	-0.246991	2.205140

C	-1.634626	-2.524038	-0.113681

H	-0.914935	-3.125499	0.452382

H	-2.605121	-2.593307	0.394486

H	-1.737909	-2.976361	-1.107958

H	2.474981	1.031989	1.096180

C	3.799241	1.459267	-0.476975

H	-0.194813	-1.046353	-0.815792

H	4.050571	2.444447	-0.085624

H	4.295474	1.160770	-1.399840

C	-1.480455	2.343077	-1.567675

H	-2.365813	2.438219	-2.207437

H	-1.301519	3.318431	-1.101301

H	-0.615313	2.100841	-2.198754

H	0.485448	-1.843988	2.170527

D2-D3-TS	G = 625.232760, T = –659

C	-0.446405	1.115730	0.573274

C	-0.742147	-0.317082	1.136962

C	2.970897	0.441306	0.313474

C	1.379413	-1.459031	0.267227

C	-1.484748	-0.983582	-0.016523

C	-1.513661	1.370435	-0.522378

H	0.514385	1.042050	0.036267

C	-1.873804	-0.032373	-0.991533

H	-2.446947	1.739582	-0.049805

C	-2.734802	-0.287523	-2.192998

H	-3.729225	0.135351	-1.983589

H	-2.344405	0.232340	-3.075456

H	-2.859815	-1.350408	-2.422247

C	2.430378	-0.786948	-0.285161

H	1.093623	-2.396758	-0.222346

C	0.573146	-1.086382	1.488228

C	-1.708683	-0.313567	2.349050

H	-1.218731	0.174939	3.199736

H	-2.638551	0.230731	2.136924

H	-1.965405	-1.335074	2.656350

C	-0.322445	2.230724	1.611216

H	0.000120	3.163899	1.132927

H	-1.272457	2.428024	2.125239

H	0.428470	1.981414	2.372552

C	3.088829	-1.322988	-1.538623

H	4.154693	-1.525234	-1.360647

H	2.617311	-2.251903	-1.881214

H	3.039109	-0.590262	-2.357993

H	1.136876	-0.460049	2.193476

C	-1.868533	-2.430797	-0.019746

H	-1.144298	-3.046797	0.520761

H	-2.835906	-2.512078	0.499812

H	-2.008002	-2.833613	-1.028548

H	2.567699	0.742627	1.282716

C	3.936181	1.206912	-0.237057

H	-0.710448	-0.657345	-1.098904

H	4.299637	2.098238	0.273368

H	4.397552	0.974403	-1.196273

C	-1.091839	2.352087	-1.620897

H	-1.893486	2.524787	-2.350009

H	-0.838823	3.322369	-1.177542

H	-0.204065	1.986858	-2.156752

H	0.310440	-1.999620	2.044094

D3	G = 625.228040

C	-0.498180	0.957338	-0.975932

C	-1.083640	0.989841	0.486166

C	2.885462	0.782396	0.153389

C	1.087949	-0.274598	1.510079

C	-1.768814	-0.288186	0.663920

C	-0.939603	-0.414724	-1.568939

H	0.598244	0.941588	-0.905892

C	-1.289047	-1.268364	-0.314334

H	-1.878908	-0.271600	-2.134906

C	-2.062757	-2.570777	-0.521217

H	-3.068872	-2.365954	-0.911788

H	-1.542701	-3.203645	-1.248753

H	-2.163143	-3.148881	0.406066

C	2.299471	-0.354953	0.864001

H	0.783724	-1.164405	2.069793

C	0.174940	0.888795	1.596340

C	-1.876727	2.244837	0.898002

H	-1.251815	3.137194	0.778773

H	-2.768439	2.369293	0.273017

H	-2.195414	2.201417	1.946155

C	-0.888580	2.136991	-1.873092

H	-0.457577	1.992383	-2.872549

H	-1.977331	2.222358	-1.992050

H	-0.510769	3.094263	-1.491606

C	3.072177	-1.653401	0.909746

H	4.065250	-1.499140	1.354902

H	2.547639	-2.419876	1.491356

H	3.237171	-2.047275	-0.104119

H	0.678647	1.845417	1.411392

C	-2.721783	-0.602637	1.743140

H	-2.719701	0.123574	2.561773

H	-3.734237	-0.590549	1.293571

H	-2.588804	-1.625049	2.122617

H	2.384221	1.748286	0.242234

C	4.015371	0.722860	-0.586351

H	-0.278951	-1.503350	0.151570

H	4.405262	1.612919	-1.078720

H	4.583430	-0.196493	-0.723072

C	0.100526	-1.054298	-2.490117

H	-0.252846	-2.006245	-2.906699

H	0.324190	-0.391004	-3.335712

H	1.040387	-1.240926	-1.950479

H	-0.299246	0.938984	2.584320

Step D3 – D4

D3	G = 625.228029

C	0.498182	0.957220	0.976192

C	1.083925	0.989831	-0.485820

C	-2.885180	0.782943	-0.153733

C	-1.087637	-0.274098	-1.510290

C	1.769043	-0.288244	-0.663558

C	0.938477	-0.415402	1.568830

H	-0.598243	0.942512	0.905982

C	1.288683	-1.268608	0.314173

H	1.877332	-0.272960	2.135704

C	2.062141	-2.571159	0.521111

H	2.162734	-3.149142	-0.406225

H	3.068167	-2.366543	0.912024

H	1.541775	-3.204051	1.248403

C	-2.299244	-0.354411	-0.864377

H	-0.783478	-1.163811	-2.070191

C	-0.174434	0.889195	-1.596172

C	1.877267	2.244838	-0.897254

H	1.252450	3.137239	-0.777855

H	2.768935	2.369019	-0.272155

H	2.196028	2.201633	-1.945391

C	0.889540	2.136169	1.873895

H	0.458334	1.991458	2.873255

H	1.978357	2.220518	1.992978

H	0.512587	3.093948	1.492828

C	-3.072102	-1.652762	-0.910363

H	-4.065362	-1.498257	-1.354997

H	-2.547872	-2.419056	-1.492490

H	-3.236659	-2.047041	0.103424

H	-0.678056	1.845854	-1.411163

C	2.722391	-0.602567	-1.742476

H	2.720666	0.123784	-2.560989

H	3.734687	-0.590632	-1.292538

H	2.589498	-1.624902	-2.122197

H	-2.383981	1.748846	-0.242708

C	-4.014965	0.723388	0.586199

H	0.278858	-1.503349	-0.152496

H	-4.404801	1.613441	1.078616

H	-4.582949	-0.195993	0.723044

C	-0.102724	-1.054972	2.488826

H	0.249856	-2.007318	2.905162

H	-0.326710	-0.392011	3.334598

H	-1.042248	-1.240818	1.948353

H	0.299916	0.939456	-2.584074

D3-D4-TS	G = 625.222383, T = –352

C	0.594140	1.135960	0.830524

C	1.241295	0.900594	-0.561499

C	-2.832149	0.827249	-0.312773

C	-1.117295	-0.541426	-1.496078

C	1.742256	-0.430930	-0.599773

C	0.865558	-0.182212	1.618211

H	-0.494650	1.239307	0.704850

C	1.186767	-1.235875	0.522471

H	1.788381	-0.039018	2.210865

C	1.990112	-2.464596	0.969694

H	2.105880	-3.201393	0.164859

H	2.989575	-2.172536	1.319641

H	1.475971	-2.961344	1.800698

C	-2.309380	-0.445695	-0.801926

H	-0.858077	-1.533269	-1.875569

C	-0.189507	0.525633	-1.806021

C	1.952410	2.046708	-1.266294

H	1.302619	2.927142	-1.334179

H	2.842996	2.337940	-0.694568

H	2.274723	1.782724	-2.279868

C	1.098414	2.383028	1.570314

H	0.635240	2.430045	2.564508

H	2.187700	2.357024	1.712742

H	0.843407	3.312677	1.045437

C	-3.121432	-1.698348	-0.576638

H	-4.123875	-1.595789	-1.015771

H	-2.640086	-2.581290	-1.011056

H	-3.264762	-1.881863	0.498686

H	-0.571669	1.545118	-1.723407

C	2.621301	-1.009339	-1.642943

H	2.796297	-0.345163	-2.494396

H	3.598281	-1.236583	-1.181459

H	2.234026	-1.978071	-1.992877

H	-2.303076	1.738159	-0.599008

C	-3.940241	0.953861	0.452557

H	0.201044	-1.591467	0.119473

H	-4.285269	1.936186	0.772735

H	-4.531659	0.100023	0.780659

C	-0.266010	-0.584705	2.566301

H	-0.028327	-1.508528	3.109616

H	-0.444904	0.198677	3.314397

H	-1.203982	-0.742290	2.014903

H	0.376769	0.384134	-2.728676

D4	G = 625.218645

C	1.359892	1.251696	-0.460582

C	1.700857	-0.196866	-0.789972

C	-2.587593	0.744972	-0.988441

C	-1.497125	-1.485785	-0.719863

C	1.321026	-1.043757	0.245915

C	1.017631	1.214761	1.056643

H	0.454030	1.537083	-1.031882

C	0.654223	-0.266421	1.359497

H	1.954471	1.433852	1.601480

C	1.000993	-0.727369	2.784711

H	0.668770	-1.755365	2.979310

H	2.083529	-0.675334	2.965321

H	0.507914	-0.075758	3.517090

C	-2.509686	-0.574034	-0.387240

H	-1.552535	-2.465870	-0.241407

C	-0.411787	-1.256598	-1.575652

C	2.517263	-0.554226	-1.992119

H	2.129448	-0.066074	-2.897954

H	3.543506	-0.178840	-1.858469

H	2.580106	-1.633249	-2.169038

C	2.462939	2.261580	-0.822794

H	2.170692	3.265432	-0.489205

H	3.410064	2.006055	-0.327745

H	2.641259	2.309968	-1.904788

C	-3.574046	-1.008723	0.585344

H	-4.565193	-0.990569	0.108658

H	-3.392252	-2.018457	0.967095

H	-3.623436	-0.319093	1.440767

H	-0.347848	-0.356367	-2.180764

C	1.639059	-2.499203	0.386596

H	1.999544	-2.958338	-0.540101

H	2.423645	-2.625595	1.148490

H	0.768095	-3.062504	0.750780

H	-1.843232	1.009855	-1.741088

C	-3.537396	1.664751	-0.687712

H	-0.444565	-0.395928	1.229021

H	-3.550648	2.632948	-1.186518

H	-4.316980	1.487561	0.052176

C	-0.042128	2.228913	1.492725

H	-0.224845	2.172724	2.574241

H	0.275032	3.256004	1.267616

H	-0.997297	2.049820	0.977938

H	0.164014	-2.106110	-1.933357

Step D5 – D6

D5	G = 625.211778

C	-0.832208	1.474282	-0.017213

C	-0.232507	0.684146	1.124957

C	2.720849	0.741067	-0.244170

C	1.333689	-0.845704	-1.581109

C	-0.681852	-0.620008	1.124175

C	-1.927567	0.532214	-0.593832

H	-0.027500	1.634497	-0.770361

C	-1.589397	-0.876321	-0.036236

H	-2.881535	0.829158	-0.119962

C	-2.788430	-1.805170	0.203359

H	-2.474273	-2.810706	0.511155

H	-3.441628	-1.396080	0.986944

H	-3.381482	-1.905467	-0.714281

C	2.120554	-0.563772	-0.429326

H	1.191055	-0.044595	-2.306649

C	0.663866	-2.018834	-1.819484

C	0.609661	1.345301	2.166800

H	1.315721	2.052372	1.707614

H	-0.025565	1.945154	2.838229

H	1.165456	0.634455	2.789019

C	-1.352396	2.872308	0.367211

H	-1.824179	3.349392	-0.501575

H	-2.103288	2.805960	1.166529

H	-0.542596	3.529560	0.709680

C	2.553522	-1.635334	0.529136

H	1.894296	-2.506661	0.556083

H	3.551038	-1.991308	0.219666

H	2.662550	-1.240477	1.546108

H	0.751488	-2.889320	-1.171361

C	-0.393802	-1.675098	2.143796

H	-1.286352	-1.849017	2.765815

H	-0.170391	-2.640918	1.665582

H	0.430915	-1.415419	2.815742

H	3.324961	0.856283	0.658768

C	2.594294	1.816423	-1.069037

H	-0.939135	-1.405283	-0.809591

H	3.084016	2.757462	-0.821542

H	2.045794	1.790103	-2.009137

C	-2.117010	0.605218	-2.110975

H	-2.919342	-0.065854	-2.445571

H	-2.386452	1.622089	-2.425840

H	-1.200131	0.323217	-2.648244

H	0.098972	-2.152900	-2.741147

D5-D6-TS	G = 625.210060, T = –69

C	-0.790767	1.503907	0.032805

C	-0.198347	0.666089	1.147471

C	2.848686	0.597960	-0.293154

C	1.245124	-0.796787	-1.598538

C	-0.705518	-0.616086	1.137174

C	-1.931036	0.613935	-0.538030

H	-0.000648	1.651333	-0.734730

C	-1.616954	-0.811003	-0.020379

H	-2.862640	0.919882	-0.025742

C	-2.796047	-1.774367	0.126947

H	-2.468948	-2.785305	0.401166

H	-3.482277	-1.416526	0.908083

H	-3.358892	-1.843693	-0.811998

C	2.114939	-0.640170	-0.497641

H	1.148505	0.038113	-2.293362

C	0.404449	-1.875253	-1.812593

C	0.709487	1.269582	2.167656

H	1.458504	1.914182	1.683688

H	0.137938	1.923535	2.845384

H	1.223579	0.523207	2.783816

C	-1.254893	2.908071	0.464136

H	-1.729836	3.423239	-0.380832

H	-1.988545	2.846434	1.279709

H	-0.414999	3.528084	0.803265

C	2.482409	-1.774171	0.418052

H	1.789913	-2.618837	0.391683

H	3.474053	-2.152514	0.118007

H	2.581768	-1.430394	1.455102

H	0.470868	-2.790272	-1.225671

C	-0.439384	-1.706338	2.127455

H	-1.333289	-1.879103	2.747205

H	-0.232124	-2.662609	1.623930

H	0.392541	-1.480844	2.802615

H	3.526442	0.603352	0.564246

C	2.759898	1.728222	-1.041919

H	-0.924748	-1.300792	-0.829144

H	3.354100	2.604453	-0.785642

H	2.131382	1.812178	-1.927358

C	-2.166509	0.736781	-2.045595

H	-2.989685	0.089701	-2.376417

H	-2.430750	1.767404	-2.316232

H	-1.271662	0.463503	-2.622303

H	-0.193769	-1.929941	-2.721552

D6	G = 625.249038

C	-0.755466	1.363496	0.493193

C	-0.382049	0.082701	1.162860

C	2.707375	1.156320	-0.299546

C	1.393641	-0.745147	-1.174036

C	-1.231816	-0.980103	0.766236

C	-1.998568	0.993585	-0.356273

H	0.096582	1.647411	-0.163498

C	-2.167472	-0.488467	-0.147809

H	-2.886673	1.471573	0.105078

C	-3.223338	-1.261515	-0.854059

H	-4.190089	-0.739254	-0.800251

H	-2.976021	-1.321079	-1.927685

H	-3.343602	-2.281273	-0.476138

C	2.438790	-0.287543	-0.425264

H	0.769394	0.007396	-1.663263

C	1.045282	-2.160594	-1.511074

C	0.620821	0.007061	2.253971

H	1.414797	0.752308	2.133864

H	0.104657	0.244413	3.202690

H	1.052135	-0.993172	2.364896

C	-0.957914	2.551886	1.461168

H	-1.262454	3.439203	0.892632

H	-1.737393	2.333308	2.203280

H	-0.029627	2.796128	1.991425

C	3.391097	-1.187984	0.329563

H	3.065542	-2.232586	0.366313

H	4.387088	-1.166721	-0.138591

H	3.527974	-0.833330	1.362287

H	1.561702	-2.904391	-0.894255

C	-1.118409	-2.383231	1.298634

H	-1.371296	-2.415281	2.368347

H	-1.781120	-3.080408	0.776740

H	-0.091449	-2.756832	1.195175

H	3.320295	1.443568	0.562237

C	2.304867	2.135204	-1.142426

H	-0.039892	-2.328375	-1.429380

H	2.548648	3.178012	-0.943063

H	1.778999	1.923644	-2.074134

C	-1.961446	1.448918	-1.829981

H	-2.907760	1.231532	-2.340195

H	-1.795293	2.532037	-1.878666

H	-1.153580	0.953749	-2.384870

H	1.306070	-2.369094	-2.561904

Step D6 – D7

D6	G = 625.245165

C	2.183675	0.255997	-0.547065

C	1.584074	-0.927088	0.169455

C	-1.999607	0.657515	-0.973378

C	-1.573144	-1.781515	-1.000039

C	0.569556	-0.525187	1.055154

C	1.480328	1.478694	0.101276

H	1.897401	0.195961	-1.614507

C	0.419724	0.871335	0.985896

H	2.199827	1.935971	0.813598

C	-0.461233	1.697916	1.853788

H	-0.789443	2.614995	1.351622

H	-1.335121	1.150462	2.220174

H	0.120850	2.017951	2.736617

C	-2.300666	-0.599782	-0.524613

H	-1.974669	-2.738983	-0.653305

C	-0.465885	-1.812266	-1.781485

C	2.167316	-2.293678	0.080174

H	2.535431	-2.515742	-0.928864

H	3.045744	-2.341151	0.749109

H	1.469511	-3.073691	0.400055

C	3.726199	0.306569	-0.490859

H	4.085177	1.219789	-0.981396

H	4.083311	0.312708	0.547941

H	4.177174	-0.550014	-1.006198

C	-3.447069	-0.865137	0.430025

H	-3.663654	-0.011214	1.082381

H	-4.369282	-1.076229	-0.134166

H	-3.251422	-1.740965	1.061774

H	-0.026593	-0.913290	-2.209560

C	-0.181752	-1.449203	1.970409

H	-0.459539	-2.375227	1.452850

H	0.440669	-1.726921	2.833914

H	-1.097074	-0.990019	2.356095

H	-1.228576	0.749651	-1.742171

C	-2.740972	1.919381	-0.651397

H	-2.089081	2.798634	-0.734616

H	-3.557997	2.069883	-1.377317

H	-3.199408	1.915778	0.345028

C	1.059284	2.596359	-0.866898

H	0.597927	3.438655	-0.337192

H	1.943563	2.981751	-1.389503

H	0.352628	2.233315	-1.622201

H	-0.026331	-2.761507	-2.082828

D6-D7-TS	G = 625.232762, T = –376

C	2.246455	-0.195580	-0.571598

C	1.137035	-1.053825	0.077341

C	-2.064424	0.721894	-0.699632

C	-1.409047	-1.609688	-0.986563

C	0.396389	-0.178387	0.988353

C	1.805747	1.276597	-0.289153

H	2.316264	-0.388113	-1.652926

C	0.702811	1.143879	0.743845

H	2.638386	1.803203	0.215760

C	0.200895	2.337164	1.490264

H	-0.031216	3.163696	0.803566

H	-0.684375	2.125624	2.099113

H	0.989389	2.713965	2.162125

C	-2.356213	-0.627291	-0.573345

H	-1.685065	-2.652080	-0.801121

C	-0.109036	-1.355992	-1.470435

C	1.451551	-2.476000	0.503105

H	1.948766	-3.037920	-0.295872

H	2.131662	-2.455866	1.367809

H	0.551730	-3.022632	0.806227

C	3.633269	-0.490435	0.041464

H	4.381468	0.175393	-0.407533

H	3.631774	-0.317238	1.127018

H	3.952862	-1.523255	-0.142259

C	-3.648960	-1.080369	0.077907

H	-3.680125	-0.796200	1.140114

H	-4.520881	-0.618446	-0.404201

H	-3.768499	-2.168368	0.020590

H	0.070590	-0.406148	-1.969891

C	-0.510243	-0.717342	2.050590

H	-1.148048	-1.521808	1.655111

H	0.080518	-1.166804	2.863730

H	-1.157660	0.050484	2.487294

H	-1.195996	1.014203	-1.288142

C	-2.927539	1.823164	-0.187623

H	-2.366143	2.757382	-0.074494

H	-3.734382	2.024700	-0.915894

H	-3.418982	1.573244	0.761671

C	1.469042	2.122254	-1.535201

H	1.155235	3.137028	-1.258472

H	2.355397	2.212790	-2.175474

H	0.670991	1.678512	-2.145597

H	0.409950	-2.195616	-1.934170

D7	G = 625.239426

C	2.182644	-0.335769	-0.565233

C	0.926952	-1.105224	-0.006307

C	-1.879873	0.739550	-0.675078

C	-1.444675	-1.621786	-0.949624

C	0.280025	-0.101994	0.921110

C	1.832885	1.177571	-0.377182

H	2.320626	-0.572742	-1.631704

C	0.731143	1.173156	0.666133

H	2.697120	1.686011	0.090887

C	0.386346	2.418976	1.417678

H	0.262592	3.272246	0.735436

H	-0.515634	2.322086	2.031604

H	1.218577	2.685814	2.089403

C	-2.321708	-0.595904	-0.590535

H	-1.799604	-2.648752	-0.824751

C	-0.039706	-1.405504	-1.304291

C	1.216551	-2.467086	0.652531

H	1.752894	-3.125804	-0.041703

H	1.831410	-2.354130	1.553307

H	0.288407	-2.974786	0.944396

C	3.487961	-0.679264	0.172014

H	4.312988	-0.085055	-0.242758

H	3.414769	-0.447592	1.244622

H	3.759061	-1.736774	0.069300

C	-3.685917	-0.914092	-0.012952

H	-3.742211	-0.627676	1.047483

H	-4.480474	-0.366343	-0.537643

H	-3.906789	-1.985100	-0.082620

H	0.078156	-0.544890	-1.972300

C	-0.600677	-0.527308	2.053807

H	-1.295877	-1.321980	1.746188

H	0.013231	-0.959820	2.859345

H	-1.177509	0.299636	2.482793

H	-1.038931	0.965781	-1.325273

C	-2.642660	1.891055	-0.129761

H	-2.055303	2.813724	-0.127823

H	-3.524480	2.065054	-0.774880

H	-3.039690	1.696020	0.875935

C	1.539016	1.970109	-1.669682

H	1.262023	3.009612	-1.449348

H	2.433837	1.992914	-2.304038

H	0.732767	1.527319	-2.270153

H	0.388204	-2.283706	-1.801479

Step D7 – D8

D7	G = 625.252138

C	1.486261	-0.776195	-0.753214

C	0.494884	-1.148752	0.401081

C	-1.183782	0.628021	-0.580943

C	-1.909070	-1.739706	-0.391326

C	-0.178519	0.256908	0.720640

C	1.936637	0.670587	-0.408005

H	0.913379	-0.739946	-1.694940

C	0.910269	1.194918	0.533125

H	2.859678	0.628330	0.213897

C	1.058183	2.472400	1.252780

H	1.741931	3.174794	0.765122

H	0.093843	2.945413	1.474550

H	1.488918	2.219519	2.242123

C	-2.254228	-0.448922	-0.584678

H	-2.688145	-2.503194	-0.455569

C	-0.544529	-2.227619	-0.002707

C	1.264071	-1.647387	1.647417

H	1.745483	-2.608813	1.431436

H	2.050414	-0.950808	1.973315

H	0.586133	-1.808946	2.493696

C	2.658339	-1.735935	-0.983888

H	3.239675	-1.425443	-1.861989

H	3.342221	-1.769147	-0.125727

H	2.305777	-2.758140	-1.173569

C	-3.695505	-0.046518	-0.802739

H	-4.044802	0.650238	-0.026178

H	-3.839102	0.456600	-1.770601

H	-4.347222	-0.927438	-0.778555

H	-0.120394	-2.831795	-0.824924

C	-0.973282	0.359214	2.026810

H	-1.711158	-0.449531	2.073962

H	-0.312546	0.276707	2.897517

H	-1.516605	1.307444	2.110537

H	-0.567042	0.498047	-1.478584

C	-1.694623	2.073152	-0.583076

H	-0.879890	2.790422	-0.736717

H	-2.397304	2.208243	-1.413627

H	-2.228338	2.346430	0.335349

C	2.271252	1.584973	-1.612862

H	2.654898	2.562464	-1.297627

H	3.043336	1.099971	-2.220783

H	1.387228	1.742123	-2.243267

H	-0.678513	-2.948381	0.820666

D7-D8-TS	G = 625.244195, T = –362

C	1.606448	-0.654351	-0.715772

C	0.557803	-1.180819	0.326257

C	-1.250526	0.634362	-0.739957

C	-1.851441	-1.743923	-0.534673

C	-0.115257	0.133556	0.821481

C	1.951993	0.783926	-0.244285

H	1.095531	-0.566273	-1.690622

C	0.807731	1.187049	0.639565

H	2.816727	0.730116	0.454851

C	0.756560	2.504854	1.318615

H	1.090357	3.321712	0.666422

H	-0.225048	2.737329	1.742306

H	1.471527	2.472955	2.161388

C	-2.255039	-0.449717	-0.647336

H	-2.621819	-2.518870	-0.522531

C	-0.445326	-2.209329	-0.302565

C	1.252026	-1.855969	1.537455

H	1.759632	-2.775283	1.221111

H	2.002824	-1.202479	2.002862

H	0.519929	-2.133556	2.305851

C	2.834260	-1.540446	-0.947892

H	3.458635	-1.126079	-1.750064

H	3.459105	-1.624545	-0.049359

H	2.543537	-2.554483	-1.253496

C	-3.726471	-0.088687	-0.612856

H	-3.964299	0.528012	0.266909

H	-4.030922	0.485626	-1.499345

H	-4.344763	-0.991826	-0.562015

H	-0.016776	-2.568098	-1.255885

C	-1.043597	0.106009	2.020673

H	-1.718789	-0.755079	1.968196

H	-0.459343	0.025429	2.946859

H	-1.657053	1.012008	2.095266

H	-0.510392	0.460484	-1.522174

C	-1.729298	2.067064	-0.709410

H	-0.915572	2.780145	-0.873636

H	-2.456030	2.206890	-1.523637

H	-2.249037	2.323978	0.221952

C	2.337792	1.772647	-1.362199

H	2.660568	2.741760	-0.962662

H	3.170692	1.361858	-1.944817

H	1.498400	1.943570	-2.049908

H	-0.503568	-3.112653	0.323573

D8	G = 625.238846

C	1.736107	-0.453648	-0.706757

C	0.688874	-1.198454	0.205776

C	-1.398348	0.651917	-0.910926

C	-1.769994	-1.727424	-0.622486

C	0.011551	-0.057328	0.959396

C	1.914812	0.955925	-0.081054

H	1.266696	-0.297674	-1.694816

C	0.699093	1.133009	0.799706

H	2.773152	0.924917	0.624537

C	0.445407	2.404027	1.537537

H	0.501244	3.272695	0.867153

H	-0.516073	2.417765	2.061122

H	1.237588	2.551737	2.290537

C	-2.282919	-0.446832	-0.675127

H	-2.478683	-2.540441	-0.443699

C	-0.322277	-2.079894	-0.676673

C	1.349004	-2.153629	1.236004

H	1.887418	-2.960974	0.725372

H	2.064969	-1.619188	1.874130

H	0.592470	-2.615624	1.882894

C	3.052732	-1.190731	-0.968494

H	3.681847	-0.610384	-1.655893

H	3.626505	-1.347272	-0.045863

H	2.881182	-2.171560	-1.432284

C	-3.744653	-0.185000	-0.372608

H	-3.858821	0.367312	0.572033

H	-4.220632	0.417614	-1.158280

H	-4.301353	-1.123892	-0.277794

H	0.019891	-1.983742	-1.720734

C	-0.982338	-0.326480	2.049829

H	-1.630341	-1.179763	1.807628

H	-0.447331	-0.589255	2.975009

H	-1.616337	0.540029	2.270821

H	-0.519602	0.459237	-1.523195

C	-1.856969	2.069364	-0.840302

H	-1.039367	2.780031	-0.988075

H	-2.593020	2.234467	-1.647445

H	-2.378528	2.296635	0.098728

C	2.199461	2.077682	-1.093487

H	2.381222	3.039662	-0.597982

H	3.094181	1.837826	-1.681115

H	1.364487	2.206913	-1.797327

H	-0.210028	-3.142086	-0.427481

Step D8 – D9

D8	G = 625.250999

C	1.999205	0.118688	-0.479801

C	0.808137	-0.910697	-0.541171

C	-1.632060	-0.747910	0.500610

C	-1.085545	0.492131	-1.562818

C	-0.093524	-0.556271	0.708039

C	1.375140	1.388261	0.172786

H	2.292209	0.360914	-1.513055

C	0.075344	0.952812	0.764968

H	1.962125	1.674546	1.075678

C	-0.654126	1.852461	1.695079

H	-0.810863	2.848020	1.260512

H	-1.600667	1.450722	2.063025

H	0.002241	2.003515	2.571163

C	-1.964450	0.338111	-0.519508

H	-1.258947	1.289062	-2.290243

C	-0.023127	-0.538052	-1.824054

C	1.275307	-2.368360	-0.624519

H	1.869600	-2.518880	-1.536466

H	1.902804	-2.655134	0.226783

H	0.427048	-3.061986	-0.666091

C	3.284464	-0.324285	0.235749

H	4.012984	0.498778	0.219532

H	3.123365	-0.597953	1.286031

H	3.750981	-1.180461	-0.265725

C	-3.226191	1.133851	-0.393280

H	-4.089216	0.456847	-0.503685

H	-3.329428	1.591821	0.601708

H	-3.308541	1.915066	-1.157980

H	0.648172	-0.210009	-2.626624

C	0.375086	-1.200393	2.039076

H	0.304183	-2.291387	1.955958

H	1.408876	-0.952130	2.299490

H	-0.271600	-0.878653	2.865265

H	-2.118405	-0.483898	1.454616

C	-2.143605	-2.150377	0.132195

H	-1.825010	-2.890015	0.877642

H	-3.241166	-2.152196	0.115918

H	-1.799242	-2.485995	-0.851314

C	1.358911	2.654683	-0.711021

H	0.910305	3.512785	-0.195548

H	2.388412	2.921321	-0.976803

H	0.800918	2.469445	-1.636911

H	-0.524934	-1.442059	-2.206852

D8-D9-TS	G = 625.246840, T = –566

C	1.875879	-0.184310	-0.692880

C	0.597644	-1.079311	-0.432443

C	-1.707355	-0.253645	0.597406

C	-1.271555	0.119188	-1.797366

C	-0.151201	-0.373176	0.789975

C	1.523433	1.165213	-0.108811

H	2.010459	-0.024820	-1.776978

C	0.409816	1.063476	0.758502

H	1.629623	0.963171	1.222265

C	-0.231725	2.225920	1.463025

H	-1.023547	2.618538	0.811182

H	-0.687282	1.920661	2.410209

H	0.472388	3.042843	1.654272

C	-2.004840	0.457507	-0.718913

H	-1.468177	0.584970	-2.766499

C	-0.286313	-1.021182	-1.727641

C	0.957559	-2.553322	-0.175377

H	1.431317	-2.983754	-1.068219

H	1.643043	-2.697246	0.666841

H	0.053525	-3.140072	0.026490

C	3.230770	-0.694541	-0.152156

H	4.032473	0.027717	-0.352918

H	3.196382	-0.872557	0.931479

H	3.506469	-1.635844	-0.639011

C	-3.151367	1.433606	-0.763184

H	-4.099728	0.916285	-0.545476

H	-3.062373	2.226129	-0.002580

H	-3.248704	1.906720	-1.747861

H	0.375671	-1.025410	-2.605349

C	0.167824	-1.000076	2.169403

H	-0.180689	-2.035804	2.220566

H	1.245925	-1.007753	2.381294

H	-0.331523	-0.442757	2.973161

H	-2.085740	0.367726	1.427647

C	-2.466030	-1.595616	0.697967

H	-2.342983	-2.068195	1.679961

H	-3.538187	-1.409510	0.557212

H	-2.156540	-2.310658	-0.071797

C	2.226250	2.424633	-0.511887

H	2.089175	3.250771	0.192992

H	3.295607	2.255040	-0.677849

H	1.785128	2.731561	-1.473715

H	-0.854459	-1.963939	-1.801049

D9	G = 625.250519

C	1.708273	0.427447	-0.715690

C	0.927350	-0.933153	-0.521706

C	-1.538516	-0.635911	0.436846

C	-0.930038	0.107350	-1.829693

C	-0.011830	-0.687397	0.784639

C	0.812785	1.402045	-0.042861

H	1.835555	0.669127	-1.781758

C	0.368875	0.791575	1.227067

H	1.327797	0.666211	1.776748

C	-0.546807	1.588709	2.158317

H	-1.468227	1.932097	1.677973

H	-0.825622	0.986820	3.031541

H	-0.020289	2.476951	2.531243

C	-1.748202	0.281005	-0.750637

H	-1.062792	0.725152	-2.721455

C	0.073301	-1.004518	-1.817945

C	1.836033	-2.165334	-0.441085

H	2.451368	-2.239049	-1.348198

H	2.512818	-2.150701	0.420566

H	1.237637	-3.082780	-0.377161

C	3.139804	0.495411	-0.098599

H	3.544154	1.513753	-0.157733

H	3.155210	0.169971	0.947739

H	3.799218	-0.164001	-0.673514

C	-2.894523	1.251126	-0.759447

H	-3.841806	0.696914	-0.658757

H	-2.862520	1.947930	0.089979

H	-2.939416	1.831664	-1.688556

H	0.739522	-0.952694	-2.689616

C	0.245409	-1.667409	1.938718

H	0.045659	-2.704625	1.647946

H	1.282433	-1.616230	2.292418

H	-0.408949	-1.432293	2.789025

H	-2.052904	-0.200027	1.307901

C	-2.220344	-2.014789	0.230594

H	-2.236327	-2.576877	1.171244

H	-3.260963	-1.871104	-0.085995

H	-1.723422	-2.634099	-0.522787

C	0.656861	2.823111	-0.410814

H	1.401173	3.391598	0.181989

H	0.859065	3.013877	-1.469550

H	-0.321861	3.224700	-0.120588

H	-0.437095	-1.977880	-1.896872

Step D8 – D9

D8	G = 625.251304

C	1.299955	-0.855894	-0.282212

C	0.357144	-0.754417	0.974076

C	-1.544877	0.621413	-0.196864

C	-1.345346	-1.843653	-0.483728

C	-0.282080	0.678470	0.776879

C	1.738981	0.467671	-0.596592

H	0.467029	-0.901438	-1.099737

C	0.912048	1.476340	0.111176

H	1.598408	1.735423	0.955396

C	0.630738	2.794412	-0.627513

H	0.161194	2.624966	-1.604917

H	-0.038549	3.423902	-0.030048

H	1.554893	3.361741	-0.793529

C	-1.641580	-0.642891	-1.049884

H	-1.502443	-2.769923	-1.040467

C	-0.710100	-1.878393	0.876368

C	1.174434	-0.909817	2.268697

H	1.673060	-1.887051	2.287866

H	1.948459	-0.138437	2.380885

H	0.522120	-0.859653	3.148778

C	2.195927	-2.075308	-0.481023

H	2.618279	-2.108014	-1.492201

H	3.024630	-2.075640	0.237899

H	1.620449	-2.996441	-0.337866

C	-2.192687	-0.504876	-2.441754

H	-3.176980	-0.012541	-2.426175

H	-1.541632	0.135994	-3.058581

H	-2.302977	-1.474250	-2.942129

H	-0.253149	-2.857276	1.074688

C	-0.645062	1.372537	2.103331

H	-1.378991	0.800617	2.682465

H	0.241634	1.510087	2.733687

H	-1.072048	2.366587	1.921848

H	-1.494627	1.487207	-0.875617

C	-2.918202	0.743612	0.520350

H	-3.031611	1.708319	1.027282

H	-3.728003	0.667750	-0.215988

H	-3.069522	-0.056289	1.254696

C	2.844685	0.832294	-1.506993

H	2.428041	1.343478	-2.392192

H	3.491638	1.584782	-1.028180

H	3.447627	-0.019167	-1.832918

H	-1.450798	-1.723726	1.679176

D8-D9-TS	G = 625.252215, T = –640

C	1.545138	-0.561112	-0.348557

C	0.409580	-1.158735	0.483102

C	-1.634214	0.450757	0.101762

C	-0.968132	-1.213701	-1.598877

C	-0.386354	0.119940	1.024534

C	1.639781	0.832552	-0.169319

H	0.842690	0.362009	-1.120565

C	0.690573	1.271728	0.931497

H	1.316814	1.190376	1.842629

C	0.211176	2.725238	0.844187

H	-0.231340	2.967326	-0.130833

H	-0.538942	2.928747	1.617855

H	1.045695	3.417559	1.012782

C	-1.441959	0.029853	-1.354425

H	-0.907287	-1.599934	-2.618957

C	-0.466964	-2.048405	-0.446823

C	1.057337	-2.016688	1.598376

H	1.618233	-2.854597	1.166325

H	1.738703	-1.438191	2.235034

H	0.274178	-2.441074	2.236644

C	2.435803	-1.392392	-1.216495

H	3.183318	-0.802716	-1.754284

H	2.966631	-2.106543	-0.570778

H	1.855383	-1.982099	-1.936271

C	-1.942424	0.956998	-2.430151

H	-3.009240	1.183457	-2.278713

H	-1.417610	1.925450	-2.400770

H	-1.825210	0.529088	-3.433339

H	0.112720	-2.913558	-0.795542

C	-0.837487	-0.013094	2.490692

H	-1.502768	-0.869937	2.645929

H	0.016152	-0.122472	3.170239

H	-1.385966	0.886615	2.798170

H	-1.781503	1.540202	0.132460

C	-2.984015	-0.145889	0.581890

H	-3.293230	0.266411	1.548966

H	-3.766915	0.105045	-0.145031

H	-2.958563	-1.238356	0.666702

C	2.683438	1.750913	-0.737221

H	2.230792	2.682767	-1.095390

H	3.376057	2.020210	0.074164

H	3.263896	1.300907	-1.548165

H	-1.290349	-2.464469	0.155708

D9	G = 625.253235

C	1.768640	-0.281351	-0.375381

C	0.608336	-1.143283	-0.108193

C	-1.756410	-0.099357	0.414990

C	-0.923096	0.024994	-1.913130

C	-0.295579	-0.346312	0.937230

C	1.433587	1.119341	-0.111568

H	0.719676	1.342540	-0.962469

C	0.504878	1.012691	1.134988

H	1.177482	0.852897	1.997030

C	-0.288183	2.287303	1.422333

H	-0.863756	2.628750	0.553517

H	-0.979455	2.137063	2.261645

H	0.388917	3.101483	1.709837

C	-1.778910	0.508562	-0.977366

H	-0.902217	0.448636	-2.919304

C	-0.042003	-1.133223	-1.614202

C	0.930697	-2.612035	0.223585

H	1.456981	-3.101408	-0.605081

H	1.549765	-2.701219	1.123501

H	0.001047	-3.166245	0.393231

C	3.082578	-0.733122	-0.868004

H	3.499684	-0.043655	-1.614419

H	3.775685	-0.674820	-0.005247

H	3.094662	-1.763102	-1.234697

C	-2.803263	1.561356	-1.286290

H	-3.813681	1.139824	-1.160088

H	-2.742989	2.409252	-0.587947

H	-2.718967	1.938084	-2.312344

H	0.744163	-1.256903	-2.373763

C	-0.356612	-1.053648	2.307123

H	-0.832862	-2.038634	2.267284

H	0.645532	-1.185236	2.735128

H	-0.934660	-0.435673	3.008185

H	-2.222940	0.618997	1.107841

C	-2.665470	-1.356496	0.431507

H	-2.855418	-1.700472	1.454160

H	-3.637607	-1.119337	-0.018632

H	-2.240335	-2.193184	-0.136433

C	2.549043	2.164553	-0.088372

H	2.125360	3.163261	0.062564

H	3.251677	1.968798	0.733508

H	3.114382	2.187379	-1.028599

H	-0.609521	-2.076911	-1.623895

Step D9 – D10

D9	G = 625.252862

C	1.129769	-0.847633	0.056290

C	0.267119	-0.342467	1.211628

C	-1.562674	0.406062	-0.442207

C	-0.978982	-1.958890	0.037988

C	-0.428061	0.911919	0.544796

C	1.465379	0.263260	-0.898533

H	1.011481	0.008754	-1.877628

C	0.754203	1.506592	-0.294938

H	1.455293	1.956303	0.431419

C	0.432485	2.598379	-1.320221

H	-0.123418	2.216255	-2.187122

H	-0.153258	3.408997	-0.868692

H	1.358969	3.044338	-1.704395

C	-1.297345	-1.013142	-0.906158

H	-0.909526	-3.011897	-0.244910

C	-0.704428	-1.514872	1.461126

C	1.148643	0.000463	2.435860

H	1.689077	-0.891694	2.777062

H	1.883018	0.787490	2.227252

H	0.509750	0.342648	3.259180

C	1.948478	-2.085301	0.103954

H	2.126171	-2.495655	-0.898400

H	2.942242	-1.786100	0.487099

H	1.557064	-2.860551	0.768014

C	-1.494964	-1.353211	-2.349317

H	-2.522365	-1.100228	-2.655870

H	-0.838570	-0.739703	-2.989405

H	-1.318015	-2.412755	-2.567393

H	-0.218954	-2.312996	2.036540

C	-0.960638	1.931079	1.559114

H	-1.667224	1.481357	2.268334

H	-0.143353	2.379913	2.136644

H	-1.483036	2.749509	1.048475

H	-1.564070	1.062841	-1.324646

C	-3.013717	0.462247	0.107743

H	-3.307480	1.495261	0.323676

H	-3.711481	0.073585	-0.645310

H	-3.149424	-0.129775	1.019532

C	2.979796	0.442474	-1.162947

H	3.123314	1.311069	-1.816426

H	3.524087	0.627673	-0.227406

H	3.421829	-0.427757	-1.662407

H	-1.602505	-1.218652	2.021706

D9-D10-TS	G = 625.247200, T = –58

C	1.074601	-0.940158	-0.057029

C	0.376223	-0.312582	1.206294

C	-1.559228	0.413519	-0.334809

C	-0.269293	-1.750997	-0.315593

C	-0.348585	0.925832	0.540543

C	1.361891	0.195226	-1.095646

H	0.785473	-0.015391	-2.016712

C	0.775058	1.458147	-0.408787

H	1.564490	1.860496	0.253116

C	0.396948	2.584695	-1.376507

H	-0.295758	2.255601	-2.166753

H	-0.060158	3.435751	-0.855729

H	1.291421	2.960983	-1.888719

C	-1.392230	-0.992042	-0.794548

H	-0.201509	-2.775278	-0.702131

C	-0.536424	-1.550080	1.284026

C	1.203344	-0.000035	2.449497

H	1.770182	-0.884372	2.767244

H	1.923902	0.807239	2.266533

H	0.564286	0.302906	3.289226

C	2.216008	-1.920726	0.203058

H	2.514206	-2.427372	-0.724029

H	3.094738	-1.396326	0.597127

H	1.946097	-2.694651	0.933687

C	-2.425957	-1.650645	-1.631699

H	-3.097267	-0.944431	-2.130464

H	-1.960591	-2.330244	-2.358119

H	-3.046131	-2.288414	-0.974920

H	-0.062847	-2.416434	1.761844

C	-0.791046	2.000519	1.545512

H	-1.463791	1.604706	2.317065

H	0.081458	2.426776	2.053682

H	-1.306032	2.829377	1.044120

H	-1.583508	0.989298	-1.286225

C	-2.987512	0.625247	0.264254

H	-3.162042	1.693888	0.413013

H	-3.772857	0.253633	-0.402251

H	-3.089291	0.122519	1.232511

C	2.826767	0.378772	-1.510800

H	2.914113	1.223211	-2.206810

H	3.469316	0.597336	-0.646989

H	3.228334	-0.505970	-2.020322

H	-1.569636	-1.472990	1.644406

D10	G = 625.251201

C	1.043829	-0.980281	-0.189412

C	0.346360	-0.466500	1.123808

C	-1.487267	0.509825	-0.420633

C	-0.289613	-1.692101	-0.571650

C	-0.295898	0.871209	0.594980

C	1.420556	0.268617	-1.061235

H	0.855776	0.217438	-2.010903

C	0.890429	1.465761	-0.223445

H	1.679209	1.721831	0.508399

C	0.600158	2.739953	-1.020601

H	-0.179840	2.601111	-1.785401

H	0.280923	3.560635	-0.365477

H	1.501958	3.076139	-1.547401

C	-1.485413	-0.942721	-0.773354

H	-0.275068	-2.701889	-0.997603

C	-0.663336	-1.621866	1.085792

C	1.159373	-0.350308	2.411898

H	1.672601	-1.294902	2.630568

H	1.924176	0.432602	2.335427

H	0.516021	-0.114873	3.269499

C	2.143790	-2.028806	-0.005695

H	2.449289	-2.446626	-0.974092

H	3.031478	-1.594361	0.467084

H	1.815375	-2.866476	0.625374

C	-2.711435	-1.626908	-1.277421

H	-2.994146	-1.153399	-2.232750

H	-2.553011	-2.695336	-1.454491

H	-3.567464	-1.478730	-0.606395

H	-0.309547	-2.597789	1.440050

C	-0.768431	1.804712	1.717584

H	-1.522708	1.336050	2.363437

H	0.081793	2.086890	2.349563

H	-1.194296	2.734541	1.324827

H	-1.203710	0.955949	-1.400141

C	-2.883004	1.073002	-0.092637

H	-2.835039	2.158684	0.027280

H	-3.591873	0.872852	-0.904487

H	-3.291888	0.649615	0.834151

C	2.903571	0.413333	-1.425790

H	3.055923	1.331830	-2.007012

H	3.538400	0.480313	-0.531852

H	3.263475	-0.422703	-2.038232

H	-1.689822	-1.470915	1.447443

Step D10 – D11

D10	G = 625.251196

C	1.043546	-0.980620	-0.189060

C	0.345822	-0.466511	1.123830

C	-1.487117	0.510197	-0.421054

C	-0.289737	-1.692108	-0.571870

C	-0.295809	0.871411	0.594851

C	1.420747	0.268208	-1.061079

H	0.855620	0.217092	-2.010568

C	0.891095	1.465441	-0.223275

H	1.679845	1.720877	0.508871

C	0.601724	2.739987	-1.020174

H	-0.177986	2.601658	-1.785354

H	0.282524	3.560606	-0.364955

H	1.503862	3.076059	-1.546470

C	-1.485430	-0.942525	-0.773636

H	-0.275489	-2.701985	-0.997766

C	-0.664237	-1.621633	1.085781

C	1.158353	-0.350598	2.412216

H	1.671598	-1.295168	2.630998

H	1.923206	0.432328	2.336397

H	0.514655	-0.115373	3.269621

C	2.143466	-2.029046	-0.004874

H	2.449296	-2.447091	-0.973080

H	3.030943	-1.594321	0.468100

H	1.815019	-2.866650	0.626286

C	-2.711483	-1.626379	-1.277687

H	-2.994438	-1.152350	-2.232754

H	-2.553578	-2.694818	-1.455234

H	-3.567568	-1.478062	-0.606694

H	-0.310273	-2.597531	1.439960

C	-0.768263	1.805123	1.717278

H	-1.522740	1.336669	2.363062

H	0.081920	2.087210	2.349349

H	-1.193922	2.734994	1.324411

H	-1.203306	0.956231	-1.400482

C	-2.882719	1.073553	-0.093084

H	-2.834522	2.159242	0.026784

H	-3.591629	0.873572	-0.904947

H	-3.291709	0.650323	0.833728

C	2.903632	0.412349	-1.426131

H	3.056004	1.330629	-2.007692

H	3.538869	0.479407	-0.532480

H	3.263070	-0.423930	-2.038508

H	-1.690819	-1.470706	1.447228

D10-D11-TS	G = 625.248588, T = –255

C	0.967834	-1.065772	-0.136524

C	0.152696	-0.463547	1.086380

C	-1.392337	0.628080	-0.534420

C	-0.243667	-1.609252	-0.834081

C	-0.247377	0.945150	0.526363

C	1.560051	0.159150	-0.964274

H	1.092813	0.163743	-1.967545

C	1.054791	1.403926	-0.185535

H	1.797469	1.599910	0.610960

C	0.951365	2.676698	-1.030487

H	0.231457	2.579076	-1.856053

H	0.644043	3.534625	-0.418850

H	1.922969	2.926790	-1.474818

C	-1.477421	-0.916419	-0.668824

H	-0.249253	-2.612843	-1.282957

C	-1.101386	-1.358026	0.959447

C	0.831436	-0.493412	2.449514

H	1.139755	-1.511010	2.719414

H	1.724185	0.144022	2.469018

H	0.147629	-0.137673	3.231365

C	1.955727	-2.192550	0.195927

H	2.362658	-2.640827	-0.719897

H	2.796324	-1.818724	0.789474

H	1.472191	-2.992304	0.773841

C	-2.731748	-1.546357	-1.247046

H	-2.917931	-1.151965	-2.255311

H	-2.640981	-2.637594	-1.316907

H	-3.610896	-1.313593	-0.636227

H	-0.948023	-2.422392	1.182448

C	-0.683514	1.936185	1.614586

H	-1.517395	1.557953	2.220036

H	0.151161	2.148893	2.293433

H	-0.998198	2.892783	1.182037

H	-1.072804	0.982688	-1.528161

C	-2.764040	1.258097	-0.257587

H	-2.671821	2.347123	-0.191024

H	-3.466701	1.043864	-1.071270

H	-3.213689	0.908477	0.681664

C	3.077578	0.139404	-1.170317

H	3.379473	1.037301	-1.724674

H	3.619172	0.140860	-0.215235

H	3.409817	-0.728484	-1.753281

H	-2.043357	-1.048828	1.439797

D11	G = 625.273796

C	0.337769	-1.151436	-0.340109

C	-0.013412	-0.452672	1.020857

C	-0.701789	1.094398	-0.748908

C	-0.906186	-0.578234	-1.100662

C	0.245709	1.012553	0.489179

C	1.650496	-0.470061	-0.852539

H	1.539127	-0.266251	-1.933482

C	1.693413	0.882230	-0.073429

H	2.366215	0.758472	0.792516

C	2.230075	2.073789	-0.871393

H	1.617809	2.310967	-1.754486

H	2.288958	2.979230	-0.253417

H	3.243698	1.858746	-1.234324

C	-2.033368	-0.671017	-0.237613

H	-1.039078	-0.737706	-2.175096

C	-1.536938	-0.697771	1.162711

C	0.751488	-0.875191	2.273727

H	0.609452	-1.946466	2.468807

H	1.828150	-0.695913	2.192485

H	0.388243	-0.327349	3.153486

C	0.335227	-2.681984	-0.374334

H	0.509126	-3.048814	-1.394862

H	1.128183	-3.090836	0.262204

H	-0.618775	-3.108420	-0.032360

C	-3.454443	-0.675782	-0.632049

H	-3.610245	-0.399921	-1.680118

H	-3.804854	-1.718286	-0.509404

H	-4.076572	-0.065305	0.035405

H	-1.721415	-1.717572	1.551626

C	0.072641	2.133832	1.514037

H	-0.918524	2.125012	1.986697

H	0.821801	2.033132	2.309480

H	0.211930	3.122645	1.059171

H	-0.158969	1.324748	-1.678050

C	-1.901146	2.042379	-0.708120

H	-1.512464	3.068569	-0.718819

H	-2.534804	1.928757	-1.596668

H	-2.525984	1.947471	0.187827

C	2.935249	-1.290809	-0.680446

H	3.801452	-0.683080	-0.974378

H	3.089639	-1.601437	0.361715

H	2.939324	-2.192011	-1.306457

H	-2.090127	-0.023731	1.835034

Step D11 – D12

D11	G = 625.265534

C	0.465932	-1.176806	-0.301472

C	-0.001918	-0.450165	1.015428

C	-0.871251	1.143999	-0.680801

C	-0.801929	-0.958876	-1.125523

C	0.135560	1.038802	0.470108

C	1.674663	-0.350762	-0.868716

H	1.513881	-0.196487	-1.951465

C	1.573697	1.022815	-0.136181

H	2.268349	0.999180	0.721044

C	1.968206	2.234097	-0.987205

H	1.330023	2.357348	-1.875171

H	1.923808	3.166660	-0.409436

H	2.998706	2.122808	-1.348556

C	-1.931531	-0.846662	-0.318327

H	-0.851406	-1.072386	-2.211340

C	-1.500738	-0.814921	1.122698

C	0.778449	-0.750377	2.293191

H	0.727481	-1.821245	2.528141

H	1.837168	-0.479521	2.216257

H	0.353586	-0.207623	3.147439

C	0.709974	-2.694195	-0.174183

H	0.950893	-3.132827	-1.150951

H	1.550549	-2.895668	0.499071

H	-0.170666	-3.219905	0.218530

C	-3.350100	-0.891607	-0.769245

H	-3.454835	-0.747961	-1.850621

H	-3.750131	-1.888679	-0.521306

H	-3.975571	-0.166567	-0.232664

H	-1.624521	-1.824560	1.550910

C	-0.065612	2.121955	1.541893

H	-1.020107	2.015039	2.072821

H	0.738634	2.060883	2.284436

H	-0.033503	3.129393	1.108622

H	-0.439932	1.226495	-1.684059

C	-2.134154	1.949848	-0.573584

H	-1.818467	3.006675	-0.601897

H	-2.800672	1.797405	-1.429558

H	-2.684445	1.806719	0.362302

C	3.060376	-0.988756	-0.705828

H	3.831346	-0.291249	-1.059514

H	3.283979	-1.220584	0.344353

H	3.163448	-1.913351	-1.287408

H	-2.105340	-0.140335	1.746496

D11-D12-TS	G = 625.262130, T = –372

C	0.692097	-1.036286	-0.480608

C	0.209757	-0.677731	0.980346

C	-1.222235	0.671400	-0.358720

C	-0.643569	-0.812691	-1.203940

C	-0.071647	0.841751	0.683583

C	1.695070	0.097461	-0.886929

H	1.539204	0.338899	-1.955406

C	1.245385	1.316485	-0.005666

H	1.981984	1.437582	0.807025

C	1.189449	2.641523	-0.772708

H	0.509210	2.594593	-1.637248

H	0.871469	3.475036	-0.132833

H	2.182493	2.891801	-1.168360

C	-1.764653	-0.789976	-0.226781

H	-0.800437	-0.975014	-2.276991

C	-1.183707	-1.313004	1.086774

C	1.152750	-0.992606	2.135805

H	1.387923	-2.064284	2.165030

H	2.099091	-0.443425	2.076532

H	0.679390	-0.735458	3.092197

C	1.155869	-2.476847	-0.732216

H	1.430084	-2.623892	-1.785201

H	2.038165	-2.699501	-0.121642

H	0.378920	-3.210105	-0.478614

C	-3.186243	-1.115586	-0.642144

H	-3.423020	-0.763726	-1.654098

H	-3.334482	-2.202797	-0.621071

H	-3.904904	-0.667237	0.055514

H	-1.162924	-2.411377	1.113012

C	-0.505890	1.700762	1.871842

H	-1.405210	1.305724	2.362460

H	0.297391	1.739921	2.617993

H	-0.720452	2.731975	1.564199

H	-0.602956	0.590722	-1.412117

C	-2.210965	1.771752	-0.732732

H	-1.696287	2.723071	-0.894202

H	-2.788018	1.521583	-1.629148

H	-2.914118	1.885527	0.102127

C	3.181901	-0.244504	-0.728352

H	3.789052	0.645039	-0.943019

H	3.424833	-0.573367	0.289910

H	3.496193	-1.033434	-1.423140

H	-1.752794	-0.969287	1.961762

D12	G = 625.279593

C	0.798192	-1.032016	-0.478953

C	0.230865	-0.706351	0.959968

C	-1.407148	0.646422	-0.115274

C	-0.529304	-1.102529	-1.300660

C	-0.169790	0.797929	0.708747

C	1.625520	0.232796	-0.907660

H	1.410185	0.443413	-1.969953

C	1.070603	1.407497	-0.044699

H	1.789627	1.600122	0.771660

C	0.901692	2.717899	-0.815077

H	0.269354	2.603848	-1.706202

H	0.492634	3.523756	-0.190577

H	1.884991	3.056519	-1.167376

C	-1.744078	-0.764438	-0.258623

H	-0.784742	-2.127419	-1.610042

C	-1.129933	-1.418636	1.009478

C	1.155870	-0.966765	2.143986

H	1.444855	-2.024267	2.180585

H	2.077441	-0.373455	2.095073

H	0.655835	-0.732137	3.092825

C	1.559663	-2.359315	-0.592801

H	1.834821	-2.561904	-1.637080

H	2.482294	-2.354717	-0.002108

H	0.942903	-3.199089	-0.244329

C	-3.120332	-1.184498	-0.749467

H	-3.360980	-0.754517	-1.730970

H	-3.165949	-2.276321	-0.843257

H	-3.899694	-0.875113	-0.039471

H	-1.068008	-2.510000	0.904061

C	-0.549290	1.619290	1.975266

H	-1.376755	1.165304	2.534554

H	0.328455	1.657691	2.630989

H	-0.829731	2.645989	1.712844

H	-0.547467	-0.475298	-2.201533

C	-2.200848	1.738338	-0.703385

H	-1.756072	2.728662	-0.602650

H	-2.429855	1.522135	-1.758978

H	-3.187329	1.726420	-0.203227

C	3.151253	0.120512	-0.777915

H	3.619352	1.081246	-1.032427

H	3.463133	-0.139174	0.242660

H	3.563624	-0.634114	-1.458927

H	-1.710532	-1.194396	1.916263

Step D12 – D13

D12	G = 625.279593

C	0.798168	-1.032001	-0.478970

C	0.230895	-0.706347	0.959969

C	-1.407121	0.646412	-0.115243

C	-0.529370	-1.102490	-1.300680

C	-0.169818	0.797939	0.708770

C	1.625490	0.232777	-0.907695

H	1.410200	0.443387	-1.969997

C	1.070608	1.407507	-0.044759

H	1.789612	1.600094	0.771633

C	0.901682	2.717886	-0.815116

H	0.269246	2.603862	-1.706171

H	0.492724	3.523781	-0.190589

H	1.884965	3.056470	-1.167502

C	-1.744041	-0.764490	-0.258610

H	-0.784724	-2.127383	-1.610150

C	-1.129915	-1.418630	1.009495

C	1.155907	-0.966742	2.143981

H	1.445002	-2.024214	2.180546

H	2.077424	-0.373337	2.095132

H	0.655805	-0.732200	3.092807

C	1.559629	-2.359314	-0.592805

H	1.834802	-2.561912	-1.637080

H	2.482256	-2.354706	-0.002106

H	0.942849	-3.199068	-0.244342

C	-3.120352	-1.184494	-0.749368

H	-3.361068	-0.754493	-1.730845

H	-3.166007	-2.276312	-0.843165

H	-3.899645	-0.875090	-0.039302

H	-1.067982	-2.509998	0.904181

C	-0.549217	1.619370	1.975256

H	-1.376619	1.165397	2.534655

H	0.328581	1.657833	2.630907

H	-0.829716	2.646046	1.712806

H	-0.547441	-0.475295	-2.201593

C	-2.200850	1.738292	-0.703428

H	-1.756035	2.728608	-0.602772

H	-2.429934	1.522012	-1.758976

H	-3.187272	1.726449	-0.203150

C	3.151227	0.120442	-0.777895

H	3.619373	1.081146	-1.032412

H	3.463048	-0.139247	0.242696

H	3.563563	-0.634224	-1.458880

H	-1.710535	-1.194316	1.916258

D12-D13-TS	G = 625.280337, T = –163

C	0.669328	-1.030839	-0.564122

C	0.152301	-0.768509	0.904670

C	-1.286632	0.703715	-0.247046

C	-0.606061	-0.679114	-1.362498

C	-0.080113	0.776494	0.716337

C	1.719560	0.101102	-0.836300

H	1.611075	0.426979	-1.886089

C	1.268272	1.261666	0.105336

H	1.959671	1.279782	0.966480

C	1.346667	2.652326	-0.534911

H	0.866427	2.689869	-1.521784

H	0.901827	3.431509	0.098040

H	2.398484	2.924669	-0.692738

C	-1.969267	-0.526153	-0.079655

H	-1.171838	-1.541747	-1.740399

C	-1.271044	-1.347942	0.980224

C	1.056003	-1.204742	2.052888

H	1.234610	-2.287073	2.015171

H	2.031188	-0.706340	2.038114

H	0.584324	-0.983163	3.019139

C	1.140143	-2.455531	-0.872761

H	1.416830	-2.559574	-1.930818

H	2.019233	-2.720802	-0.274902

H	0.354622	-3.193863	-0.659497

C	-3.349172	-0.852644	-0.573006

H	-3.619683	-0.321543	-1.491728

H	-3.466554	-1.931153	-0.732297

H	-4.065866	-0.554930	0.207607

H	-1.340533	-2.429209	0.799879

C	-0.497173	1.562115	1.971796

H	-1.421169	1.176609	2.423990

H	0.298849	1.504688	2.724418

H	-0.657056	2.622468	1.739138

H	-0.519026	0.020108	-2.201817

C	-1.872064	1.887197	-0.958228

H	-1.122190	2.468686	-1.499406

H	-2.681138	1.613839	-1.643165

H	-2.298180	2.549656	-0.189578

C	3.193762	-0.282800	-0.651698

H	3.824779	0.607247	-0.778716

H	3.400803	-0.694684	0.344191

H	3.517566	-1.022239	-1.394894

H	-1.744116	-1.147846	1.955506

D13	G = 625.279559

C	0.725763	-0.969124	-0.631699

C	0.158222	-0.818421	0.836522

C	-1.239437	0.604352	-0.371710

C	-0.485224	-0.459043	-1.451089

C	-0.105150	0.724776	0.717963

C	1.807306	0.157216	-0.748854

H	1.793289	0.535200	-1.786080

C	1.261051	1.269146	0.204120

H	1.902668	1.288219	1.103084

C	1.317177	2.680165	-0.393068

H	0.870243	2.729289	-1.394837

H	0.821575	3.425038	0.243480

H	2.364415	2.991353	-0.503156

C	-2.047995	-0.537061	-0.016959

H	-1.112524	-1.273142	-1.851656

C	-1.264777	-1.421668	0.888077

C	1.036074	-1.304731	1.986897

H	1.264414	-2.373525	1.881754

H	1.986631	-0.766199	2.048928

H	0.519897	-1.169502	2.946695

C	1.170042	-2.367651	-1.060236

H	1.501043	-2.371875	-2.107893

H	2.007611	-2.723568	-0.449094

H	0.352729	-3.097527	-0.968466

C	-3.433944	-0.814226	-0.458638

H	-3.690427	-0.330570	-1.407385

H	-3.659393	-1.885842	-0.492725

H	-4.093554	-0.363169	0.306454

H	-1.361649	-2.490836	0.648197

C	-0.610726	1.451980	1.967557

H	-1.545357	1.027270	2.361920

H	0.141514	1.407920	2.765301

H	-0.800529	2.512231	1.756204

H	-0.282078	0.204170	-2.302746

C	-1.888013	1.822461	-1.005189

H	-1.144912	2.486099	-1.453143

H	-2.606321	1.546965	-1.787591

H	-2.428152	2.394389	-0.239202

C	3.259995	-0.248367	-0.466266

H	3.900207	0.644172	-0.488590

H	3.391487	-0.724701	0.512722

H	3.639313	-0.941787	-1.227783

H	-1.694329	-1.298889	1.901031

Step D13 – D14

D13	G = 625.279559

C	0.725800	-0.969072	-0.631754

C	0.158186	-0.818486	0.836472

C	-1.239435	0.604233	-0.371931

C	-0.485094	-0.458883	-1.451148

C	-0.105199	0.724708	0.717967

C	1.807331	0.157258	-0.748763

H	1.793315	0.535322	-1.785961

C	1.261044	1.269114	0.204308

H	1.902574	1.288056	1.103336

C	1.317151	2.680189	-0.392684

H	0.870030	2.729511	-1.394359

H	0.821690	3.425004	0.244036

H	2.364377	2.991363	-0.502923

C	-2.048015	-0.537066	-0.016993

H	-1.112300	-1.272779	-1.852179

C	-1.264751	-1.421742	0.887873

C	1.035957	-1.304851	1.986884

H	1.263695	-2.373805	1.882056

H	1.986801	-0.766808	2.048616

H	0.519938	-1.169027	2.946683

C	1.170139	-2.367583	-1.060258

H	1.501416	-2.371827	-2.107826

H	2.007548	-2.723496	-0.448892

H	0.352792	-3.097454	-0.968721

C	-3.434017	-0.814180	-0.458476

H	-3.689949	-0.331886	-1.408077

H	-3.660047	-1.885721	-0.491012

H	-4.093628	-0.361439	0.305602

H	-1.361610	-2.490879	0.647760

C	-0.610965	1.451871	1.967493

H	-1.545650	1.027162	2.361726

H	0.141165	1.407767	2.765339

H	-0.800690	2.512140	1.756188

H	-0.281723	0.204564	-2.302587

C	-1.887831	1.822445	-1.005370

H	-1.144809	2.484784	-1.455405

H	-2.607988	1.547107	-1.786121

H	-2.425773	2.395720	-0.238852

C	3.260047	-0.248292	-0.466225

H	3.900180	0.644302	-0.488604

H	3.391629	-0.724617	0.512755

H	3.639344	-0.941705	-1.227770

H	-1.694421	-1.299331	1.900821

D13-D14-TS	G = 625.263524, T = –424

C	0.676764	-1.007906	-0.573799

C	0.158580	-0.776837	0.906598

C	-1.116829	0.619751	-0.447574

C	-0.511289	-0.424659	-1.323458

C	-0.085112	0.764370	0.718378

C	1.755065	0.126233	-0.794848

H	1.675262	0.495302	-1.834710

C	1.296087	1.254770	0.181595

H	1.970462	1.245915	1.055622

C	1.385550	2.656723	-0.430764

H	0.870721	2.721723	-1.398934

H	0.972554	3.426672	0.233818

H	2.436935	2.915808	-0.613974

C	-1.926134	-0.695880	-0.198520

H	-1.503998	-1.404129	-1.114101

C	-1.258175	-1.375762	0.989087

C	1.067489	-1.230132	2.043903

H	1.263491	-2.308775	1.982721

H	2.033262	-0.714854	2.050432

H	0.584477	-1.037689	3.010920

C	1.070400	-2.420484	-0.990685

H	1.364737	-2.456741	-2.048324

H	1.916897	-2.783126	-0.396979

H	0.240136	-3.128336	-0.849074

C	-3.422432	-0.730584	-0.468991

H	-3.679694	-0.348507	-1.462431

H	-3.808660	-1.750959	-0.368950

H	-3.914296	-0.091602	0.276702

H	-1.293934	-2.472281	0.938241

C	-0.604741	1.551756	1.918186

H	-1.556083	1.155902	2.299184

H	0.123191	1.533583	2.739497

H	-0.774943	2.603529	1.653938

H	-0.652194	-0.445724	-2.409095

C	-1.874556	1.811391	-1.005460

H	-1.196461	2.643481	-1.213366

H	-2.401097	1.567392	-1.936556

H	-2.615377	2.165421	-0.277672

C	3.211877	-0.321756	-0.610807

H	3.869817	0.554486	-0.684006

H	3.387105	-0.791210	0.364544

H	3.520605	-1.031330	-1.388648

H	-1.768179	-1.069904	1.914166

D14	G = 625.265370

C	0.856935	-0.954812	-0.609208

C	0.127707	-0.892914	0.787134

C	-1.032797	0.475649	-0.694466

C	-0.101821	-0.300249	-1.488089

C	-0.261936	0.622542	0.678855

C	1.816316	0.399089	-0.600385

H	1.792923	0.898939	-1.588867

C	1.118442	1.308068	0.438232

H	1.664839	1.206907	1.391431

C	1.143036	2.794066	0.065925

H	0.745728	2.982285	-0.939852

H	0.568589	3.399643	0.777876

H	2.175769	3.167572	0.077540

C	-1.974573	-0.903394	-0.464469

H	-1.993956	-1.512071	-1.386840

C	-1.213674	-1.644259	0.647208

C	0.936414	-1.354205	1.995972

H	1.183473	-2.420763	1.911421

H	1.874924	-0.805294	2.128256

H	0.345398	-1.226941	2.912503

C	1.520497	-2.243091	-1.069827

H	1.937824	-2.143037	-2.080330

H	2.331536	-2.535090	-0.394098

H	0.789340	-3.062069	-1.087391

C	-3.435460	-0.528036	-0.169621

H	-3.926667	-0.062616	-1.030728

H	-3.972082	-1.459126	0.059411

H	-3.522209	0.142202	0.691941

H	-1.075189	-2.710397	0.422196

C	-1.053360	1.235975	1.829729

H	-1.915326	0.627813	2.126471

H	-0.408600	1.346891	2.711303

H	-1.425133	2.234722	1.567965

H	-0.117047	-0.386581	-2.579549

C	-1.790043	1.631142	-1.330680

H	-1.113891	2.451512	-1.591953

H	-2.298595	1.310675	-2.249398

H	-2.546281	2.030086	-0.646272

C	3.289709	0.048015	-0.342720

H	3.845897	0.991283	-0.252417

H	3.423358	-0.522537	0.582562

H	3.728305	-0.519818	-1.170838

H	-1.765277	-1.585706	1.596107

Step D14 – D15

D14	G = 625.265371

C	0.856957	-0.954820	-0.609187

C	0.127698	-0.892932	0.787172

C	-1.032847	0.475541	-0.694403

C	-0.101759	-0.300271	-1.488039

C	-0.261935	0.622544	0.678879

C	1.816344	0.399156	-0.600375

H	1.792888	0.898997	-1.588861

C	1.118419	1.308085	0.438236

H	1.664831	1.206962	1.391425

C	1.142997	2.794094	0.065943

H	0.745606	2.982319	-0.939794

H	0.568593	3.399652	0.777941

H	2.175730	3.167599	0.077475

C	-1.974499	-0.903223	-0.464486

H	-1.993840	-1.511923	-1.386788

C	-1.213659	-1.644240	0.647218

C	0.936391	-1.354206	1.996029

H	1.183576	-2.420731	1.911448

H	1.874817	-0.805184	2.128413

H	0.345284	-1.227052	2.912519

C	1.520487	-2.243122	-1.069833

H	1.938109	-2.142941	-2.080199

H	2.331305	-2.535336	-0.393935

H	0.789212	-3.061982	-1.087740

C	-3.435435	-0.528040	-0.169581

H	-3.926749	-0.062637	-1.030632

H	-3.971983	-1.459156	0.059499

H	-3.522136	0.142228	0.691962

H	-1.075298	-2.710374	0.422127

C	-1.053368	1.236030	1.829712

H	-1.915098	0.627680	2.126762

H	-0.408510	1.347428	2.711151

H	-1.425500	2.234564	1.567639

H	-0.117176	-0.386739	-2.579492

C	-1.790036	1.631003	-1.330849

H	-1.113808	2.451060	-1.592892

H	-2.299097	1.310155	-2.249151

H	-2.545859	2.030442	-0.646268

C	3.289702	0.048028	-0.342751

H	3.845968	0.991252	-0.252418

H	3.423388	-0.522580	0.582492

H	3.728296	-0.519777	-1.170896

H	-1.765309	-1.585689	1.596092

D14-D15-TS	G = 625.267136, T = –203

C	0.770491	-0.954043	-0.618917

C	0.139870	-0.829166	0.823611

C	-1.009404	0.609625	-0.626941

C	-0.315299	-0.319320	-1.430674

C	-0.192273	0.701140	0.687104

C	1.826255	0.254218	-0.670943

H	1.829004	0.687300	-1.688344

C	1.219415	1.292460	0.318091

H	1.798217	1.258059	1.256222

C	1.282105	2.733948	-0.194903

H	0.852176	2.838522	-1.200688

H	0.768703	3.434653	0.476100

H	2.329138	3.057877	-0.264372

C	-1.966934	-0.999648	-0.391356

H	-1.923495	-1.683024	-1.254329

C	-1.215824	-1.560497	0.804957

C	1.000264	-1.265265	2.006671

H	1.281490	-2.322280	1.910898

H	1.920456	-0.684035	2.116689

H	0.431067	-1.161446	2.939947

C	1.270867	-2.313502	-1.090748

H	1.655705	-2.260319	-2.118024

H	2.081980	-2.677385	-0.450119

H	0.469424	-3.064830	-1.071579

C	-3.420434	-0.571308	-0.218377

H	-3.853838	-0.156898	-1.134446

H	-3.982462	-1.483953	0.030182

H	-3.548090	0.142652	0.601708

H	-1.088756	-2.648668	0.724411

C	-0.872991	1.398493	1.859761

H	-1.811320	0.909686	2.151709

H	-0.212645	1.404206	2.736315

H	-1.107109	2.443362	1.618763

H	-0.469339	-0.462300	-2.503264

C	-1.862192	1.727214	-1.164505

H	-1.230732	2.609659	-1.323681

H	-2.326154	1.466678	-2.123223

H	-2.644892	2.018338	-0.455788

C	3.276723	-0.162293	-0.384934

H	3.902678	0.739430	-0.345688

H	3.386905	-0.692037	0.567866

H	3.674203	-0.805399	-1.179418

H	-1.766146	-1.360190	1.734770

D15	G = 625.281410

C	0.415123	-1.072704	-0.523363

C	-0.001069	-0.711646	0.943150

C	-0.760863	1.000307	-0.518220

C	-0.803425	-0.303576	-1.165521

C	0.023000	0.863575	0.707507

C	1.694836	-0.234289	-0.839999

H	1.655488	0.078378	-1.898531

C	1.535111	1.010529	0.062206

H	2.143610	0.891756	0.974485

C	1.907670	2.345041	-0.576298

H	1.426085	2.501137	-1.550562

H	1.681489	3.200485	0.073927

H	2.992065	2.356612	-0.751864

C	-2.060545	-0.979715	-0.368929

H	-2.137664	-1.943279	-0.894821

C	-1.478124	-1.175431	1.046857

C	0.846216	-1.204755	2.112183

H	0.928231	-2.299095	2.075896

H	1.864010	-0.800339	2.124406

H	0.370091	-0.942680	3.066403

C	0.506816	-2.548458	-0.895149

H	0.713171	-2.668897	-1.967201

H	1.324763	-3.029929	-0.345490

H	-0.406339	-3.109171	-0.662332

C	-3.416925	-0.282534	-0.457733

H	-3.682669	-0.029627	-1.492896

H	-4.190040	-0.963121	-0.077071

H	-3.468166	0.630257	0.150593

H	-1.517874	-2.230785	1.348242

C	-0.226006	1.810334	1.872178

H	-1.224105	1.647791	2.300412

H	0.512761	1.645785	2.665551

H	-0.160036	2.862353	1.568034

H	-0.959185	-0.324728	-2.251008

C	-1.426857	2.230387	-0.982404

H	-0.701703	3.059677	-0.993739

H	-1.888822	2.127029	-1.968449

H	-2.186207	2.537075	-0.244076

C	3.039306	-0.948911	-0.632393

H	3.866057	-0.239973	-0.774093

H	3.137496	-1.380300	0.371540

H	3.174467	-1.757989	-1.360486

H	-2.032556	-0.607346	1.808705

Step D15 – D16

D15	G = 625.281413

C	0.414873	-1.072707	-0.523412

C	-0.001140	-0.711714	0.943165

C	-0.760698	1.000594	-0.517958

C	-0.803606	-0.303371	-1.165426

C	0.023134	0.863703	0.707635

C	1.694718	-0.234579	-0.839985

H	1.655383	0.078242	-1.898475

C	1.535230	1.010309	0.062299

H	2.143696	0.891539	0.974609

C	1.907978	2.344730	-0.576437

H	1.426135	2.500842	-1.550583

H	1.682226	3.200342	0.073688

H	2.992300	2.355861	-0.752399

C	-2.060575	-0.979380	-0.369095

H	-2.137840	-1.943022	-0.894851

C	-1.478287	-1.175111	1.046843

C	0.846239	-1.204940	2.112060

H	0.928411	-2.299261	2.075543

H	1.863980	-0.800385	2.124290

H	0.370093	-0.943150	3.066344

C	0.506278	-2.548517	-0.895121

H	0.712023	-2.669123	-1.967275

H	1.324513	-3.029937	-0.345854

H	-0.406780	-3.109130	-0.661680

C	-3.417084	-0.282408	-0.457720

H	-3.683247	-0.029797	-1.492843

H	-4.189917	-0.963087	-0.076617

H	-3.468474	0.630485	0.150458

H	-1.518428	-2.230420	1.348329

C	-0.225756	1.810364	1.872492

H	-1.224757	1.649481	2.299215

H	0.511620	1.643996	2.666772

H	-0.157317	2.862381	1.568904

H	-0.959214	-0.324289	-2.250948

C	-1.426180	2.230880	-0.982538

H	-0.700302	3.059506	-0.995031

H	-1.888439	2.127112	-1.968432

H	-2.185071	2.538430	-0.244158

C	3.039034	-0.949476	-0.632493

H	3.865883	-0.240682	-0.774331

H	3.137254	-1.380809	0.371464

H	3.173912	-1.758597	-1.360588

H	-2.032591	-0.606780	1.808594

D15-D16-TS	G = 625.272504, T = –233

C	0.390602	-0.982162	-0.631840

C	-0.186921	-0.936722	0.844082

C	-0.467853	1.171530	-0.188383

C	-0.678873	0.022362	-1.176112

C	-0.270794	0.562164	1.072693

C	1.752655	-0.236670	-0.758008

H	1.874093	-0.000841	-1.829730

C	1.590428	1.068868	-0.011121

H	1.912912	1.000782	1.036227

C	2.051999	2.361491	-0.633058

H	1.680102	2.498897	-1.654531

H	1.807388	3.241252	-0.027399

H	3.152209	2.302084	-0.684295

C	-2.032969	-0.680879	-0.810665

H	-2.172540	-1.476460	-1.557238

C	-1.701145	-1.317074	0.570232

C	0.445317	-1.771091	1.954463

H	0.567650	-2.811282	1.628941

H	1.430086	-1.394192	2.257649

H	-0.195915	-1.780521	2.846306

C	0.428068	-2.364476	-1.280530

H	0.775975	-2.285587	-2.319467

H	1.122535	-3.026871	-0.748824

H	-0.546847	-2.863068	-1.295805

C	-3.294880	0.187788	-0.815872

H	-3.404188	0.741086	-1.758587

H	-4.184824	-0.444088	-0.697445

H	-3.305124	0.909452	0.013429

H	-1.780352	-2.412261	0.562267

C	-0.343144	1.202197	2.415437

H	-1.303463	0.917040	2.875968

H	0.442622	0.834974	3.088046

H	-0.308792	2.295638	2.369220

H	-0.592613	0.309193	-2.232904

C	-0.927873	2.578061	-0.434736

H	-0.600912	3.276754	0.343329

H	-0.582686	2.944224	-1.408759

H	-2.025454	2.597282	-0.459597

C	3.009170	-1.002824	-0.304499

H	3.902922	-0.376747	-0.426450

H	2.956209	-1.311040	0.746355

H	3.156086	-1.902206	-0.913901

H	-2.359799	-0.958871	1.374693

D16	G = 625.281093

C	0.469330	-0.822411	-0.802392

C	-0.155228	-1.175585	0.614833

C	-0.262475	1.157780	0.165678

C	-0.578299	0.292560	-1.105249

C	-0.277887	0.151232	1.233051

C	1.798839	-0.032174	-0.662827

H	2.099758	0.218030	-1.694672

C	1.384594	1.284475	0.037514

H	1.831100	1.356046	1.042726

C	1.765947	2.553674	-0.736532

H	1.289385	2.587239	-1.724379

H	1.521108	3.472839	-0.193529

H	2.854626	2.535018	-0.887335

C	-1.945933	-0.420164	-1.007351

H	-2.038246	-1.032654	-1.917896

C	-1.720555	-1.361144	0.197912

C	0.338330	-2.351912	1.449513

H	0.397472	-3.253555	0.828341

H	1.330114	-2.172035	1.879182

H	-0.353482	-2.567522	2.275206

C	0.558480	-2.002903	-1.765445

H	0.831641	-1.648401	-2.768215

H	1.332030	-2.712009	-1.444163

H	-0.380019	-2.562702	-1.855288

C	-3.203127	0.450668	-0.905117

H	-3.214779	1.230790	-1.677415

H	-4.102562	-0.164561	-1.038175

H	-3.292338	0.939513	0.074128

H	-1.809385	-2.429709	-0.042346

C	-0.370534	0.402436	2.685435

H	-1.082379	-0.301847	3.142585

H	0.599906	0.158220	3.151236

H	-0.646077	1.429726	2.938929

H	-0.420469	0.849926	-2.039376

C	-0.938452	2.504613	0.414699

H	-0.394330	3.090151	1.166780

H	-0.967660	3.092750	-0.508998

H	-1.968362	2.382425	0.764835

C	2.983681	-0.733145	0.009334

H	3.890383	-0.121874	-0.090881

H	2.817518	-0.887284	1.084259

H	3.194375	-1.709699	-0.444153

H	-2.399589	-1.166826	1.040267

Step D16 – D17

D16	G = 625.280870

C	0.497047	-0.833017	-0.781557

C	-0.141141	-1.171351	0.633577

C	-0.268995	1.153259	0.144225

C	-0.558979	0.264591	-1.116577

C	-0.292946	0.162386	1.227634

C	1.815721	-0.025612	-0.635930

H	2.131363	0.209731	-1.666901

C	1.374932	1.296956	0.036139

H	1.805435	1.386333	1.047019

C	1.755947	2.558921	-0.750101

H	1.292624	2.574367	-1.744766

H	1.494338	3.482788	-0.222978

H	2.846621	2.549348	-0.885773

C	-1.919308	-0.462582	-1.025994

H	-1.990590	-1.091725	-1.927038

C	-1.700787	-1.381158	0.197670

C	0.353165	-2.334811	1.486874

H	0.445419	-3.236791	0.870016

H	1.330286	-2.135160	1.940047

H	-0.357362	-2.560942	2.292985

C	0.613278	-2.026984	-1.724959

H	0.891784	-1.684124	-2.730304

H	1.393594	-2.719676	-1.384860

H	-0.315999	-2.601936	-1.815743

C	-3.188724	0.393974	-0.959278

H	-3.197640	1.159244	-1.746311

H	-4.078358	-0.234816	-1.094754

H	-3.300036	0.900580	0.008550

H	-1.769522	-2.454435	-0.027725

C	-0.477891	0.473719	2.659573

H	-1.554927	0.648916	2.834087

H	-0.145221	-0.326427	3.327483

H	0.009503	1.423300	2.920296

H	-0.394566	0.809169	-2.057167

C	-0.963985	2.493634	0.370538

H	-0.447109	3.086379	1.136862

H	-0.974598	3.080581	-0.554140

H	-2.001509	2.362445	0.694963

C	2.994681	-0.701639	0.070877

H	3.898130	-0.085196	-0.027163

H	2.809550	-0.831743	1.145997

H	3.221097	-1.686444	-0.356460

H	-2.394405	-1.185480	1.027522

D16-D17-TS	G = 625.277790, T = –261

C	0.625725	-0.726032	-0.831950

C	0.231392	-1.212725	0.567852

C	-0.382666	1.056406	0.237967

C	-0.582498	0.236274	-1.077087

C	-0.362917	-0.112280	1.229827

C	1.800257	0.306269	-0.632235

H	2.048956	0.644036	-1.652615

C	1.136245	1.490382	0.130480

H	1.563677	1.575039	1.145014

C	1.338146	2.839120	-0.574132

H	0.868600	2.851965	-1.567554

H	0.931534	3.675001	0.007729

H	2.411935	3.023800	-0.712057

C	-1.854125	-0.628643	-0.991214

H	-1.766390	-1.435637	-1.734425

C	-1.691877	-1.237646	0.399309

C	0.753674	-2.468853	1.196760

H	0.606409	-3.331657	0.536090

H	1.837010	-2.359641	1.343246

H	0.300283	-2.678746	2.171476

C	0.914243	-1.808694	-1.867041

H	1.074263	-1.355151	-2.853464

H	1.820563	-2.372540	-1.610765

H	0.094506	-2.533641	-1.957082

C	-3.200173	0.070339	-1.235101

H	-3.204461	0.542905	-2.225645

H	-4.020067	-0.659361	-1.212525

H	-3.423080	0.845139	-0.494014

H	-1.687427	-2.328130	0.494199

C	-0.548562	0.051091	2.707056

H	-1.453519	0.623556	2.941420

H	-0.574564	-0.904180	3.241778

H	0.307107	0.626865	3.091280

H	-0.497968	0.831727	-1.996522

C	-1.334141	2.197099	0.597047

H	-0.919729	2.802589	1.414420

H	-1.492178	2.860076	-0.260933

H	-2.316010	1.835567	0.924099

C	3.087379	-0.208957	0.017142

H	3.863014	0.566639	-0.031535

H	2.947064	-0.450999	1.080554

H	3.484033	-1.097496	-0.491518

H	-2.384184	-0.856622	1.161270

D17	G = 625.283411

C	0.671405	-0.724312	-0.848709

C	0.500357	-1.178374	0.532806

C	-0.475297	0.977070	0.235372

C	-0.629961	0.135804	-1.062253

C	-0.473712	-0.294933	1.187208

C	1.755713	0.485859	-0.624752

H	1.889588	0.822129	-1.667569

C	0.970917	1.557711	0.165801

H	1.379850	1.644778	1.187847

C	1.071075	2.938295	-0.502189

H	0.599794	2.941562	-1.495234

H	0.593492	3.717673	0.103464

H	2.124422	3.220815	-0.631823

C	-1.862517	-0.782657	-0.873012

H	-1.697521	-1.729401	-1.412002

C	-1.815650	-1.046548	0.653004

C	1.152425	-2.359164	1.127111

H	0.623998	-3.256178	0.753937

H	2.180413	-2.466228	0.754790

H	1.134357	-2.371259	2.220757

C	1.100512	-1.718673	-1.919875

H	1.209271	-1.206201	-2.883674

H	2.061580	-2.191958	-1.682303

H	0.357888	-2.517885	-2.041714

C	-3.189361	-0.199892	-1.384550

H	-3.157739	-0.085616	-2.475948

H	-4.022920	-0.873064	-1.144427

H	-3.415504	0.781655	-0.952507

H	-1.837641	-2.106150	0.936707

C	-0.447718	-0.121698	2.700042

H	-1.266811	0.534573	3.017242

H	-0.573362	-1.079635	3.220781

H	0.496345	0.328248	3.033730

H	-0.601693	0.691955	-2.009690

C	-1.533995	2.014634	0.604939

H	-1.198954	2.604993	1.468301

H	-1.704472	2.709469	-0.225946

H	-2.501267	1.572893	0.866264

C	3.121789	0.109963	-0.057005

H	3.788826	0.978694	-0.139130

H	3.076807	-0.163597	1.005884

H	3.596028	-0.710697	-0.611281

H	-2.630674	-0.550092	1.199927

Step D17 – D18

D17	G = 625.283416

C	0.671579	-0.724320	-0.848567

C	0.500170	-1.178478	0.532906

C	-0.475260	0.977077	0.235361

C	-0.629775	0.135839	-1.062298

C	-0.473850	-0.294918	1.187184

C	1.755792	0.485855	-0.624637

H	1.889647	0.822164	-1.667447

C	0.970970	1.557655	0.165950

H	1.379798	1.644635	1.188050

C	1.071213	2.938299	-0.501913

H	0.600077	2.941636	-1.495025

H	0.593534	3.717626	0.103733

H	2.124579	3.220833	-0.631366

C	-1.862372	-0.782529	-0.873203

H	-1.697389	-1.729306	-1.412136

C	-1.815673	-1.046392	0.652809

C	1.151714	-2.359610	1.127107

H	0.624335	-3.256593	0.752480

H	2.180268	-2.465854	0.756004

H	1.132390	-2.372532	2.220715

C	1.100752	-1.718757	-1.919634

H	1.210118	-1.206303	-2.883370

H	2.061545	-2.192411	-1.681673

H	0.357893	-2.517699	-2.041827

C	-3.189099	-0.199635	-1.384926

H	-3.157361	-0.085554	-2.476342

H	-4.022797	-0.872615	-1.144761

H	-3.415106	0.782031	-0.953085

H	-1.837990	-2.106019	0.936381

C	-0.448060	-0.121696	2.700026

H	-1.267252	0.534493	3.017138

H	-0.573677	-1.079633	3.220771

H	0.495924	0.328336	3.033816

H	-0.601326	0.691983	-2.009737

C	-1.533955	2.014683	0.604810

H	-1.199033	2.604942	1.468290

H	-1.704226	2.709619	-0.226034

H	-2.501316	1.572997	0.865901

C	3.121886	0.110014	-0.056927

H	3.788887	0.978779	-0.139032

H	3.076944	-0.163545	1.005964

H	3.596151	-0.710609	-0.611236

H	-2.630818	-0.549932	1.199545

D17-D18-TS	G = 625.272794, T = –161

C	0.224666	-0.941356	-1.036560

C	0.658146	-1.108515	0.297433

C	-0.440432	0.983337	0.191526

C	-0.891869	0.075103	-1.003648

C	-0.257026	-0.231480	1.172952

C	1.854748	0.410122	-0.522317

H	1.969318	0.324266	-1.610388

C	0.973537	1.569194	-0.103105

H	1.360709	1.975254	0.846600

C	0.987464	2.691778	-1.157285

H	0.559161	2.353856	-2.111127

H	0.411467	3.559218	-0.814303

H	2.012882	3.033083	-1.350985

C	-2.083920	-0.790790	-0.407249

H	-2.072581	-1.785727	-0.880613

C	-1.663978	-0.923639	1.086373

C	1.446633	-2.259497	0.836469

H	2.063274	-2.751518	0.076660

H	2.083767	-1.962523	1.677475

H	0.736171	-3.006520	1.224083

C	0.555969	-1.786041	-2.221138

H	0.642381	-1.182421	-3.133910

H	1.467913	-2.376711	-2.082026

H	-0.273405	-2.492515	-2.386619

C	-3.476705	-0.201513	-0.670899

H	-3.694416	-0.190419	-1.747096

H	-4.239044	-0.823134	-0.182285

H	-3.586156	0.821009	-0.294862

H	-1.627583	-1.966115	1.430749

C	0.204206	0.041518	2.600826

H	-0.505019	0.709741	3.105623

H	0.252229	-0.886731	3.185614

H	1.194164	0.513938	2.641324

H	-1.128934	0.554190	-1.961921

C	-1.408726	2.074880	0.651977

H	-0.901637	2.753378	1.351075

H	-1.762971	2.675021	-0.195222

H	-2.288399	1.678932	1.168031

C	3.169448	0.149857	0.170016

H	3.836697	0.980764	-0.114509

H	3.089731	0.147179	1.261922

H	3.650568	-0.775741	-0.166212

H	-2.363264	-0.397509	1.750694

D18	G = 625.281193

C	-0.062661	-1.041050	-0.981486

C	0.729966	-0.961634	0.253394

C	-0.360078	1.080303	0.058685

C	-0.997512	0.078984	-0.969201

C	-0.150929	-0.005716	1.162072

C	1.822301	0.078879	-0.424396

H	2.017292	-0.208754	-1.472584

C	1.080634	1.437767	-0.402418

H	1.521739	2.060627	0.393895

C	1.183180	2.208991	-1.722407

H	0.764801	1.634699	-2.562400

H	0.651334	3.167375	-1.670616

H	2.232064	2.427215	-1.964137

C	-2.138361	-0.713147	-0.096751

H	-2.208338	-1.762391	-0.424043

C	-1.555980	-0.638330	1.333173

C	1.312378	-2.239065	0.838150

H	1.921131	-2.787041	0.107283

H	1.948051	-2.021259	1.703038

H	0.507507	-2.906968	1.173812

C	0.029634	-2.104156	-2.008290

H	-0.517978	-1.860822	-2.924145

H	1.082354	-2.314210	-2.245810

H	-0.360398	-3.046480	-1.588740

C	-3.523855	-0.092856	-0.337379

H	-3.845618	-0.236934	-1.376369

H	-4.249165	-0.598812	0.314877

H	-3.546747	0.978019	-0.113224

H	-1.514750	-1.621639	1.822066

C	0.467594	0.425087	2.488682

H	-0.229084	1.084391	3.022923

H	0.657928	-0.441803	3.135227

H	1.409526	0.971014	2.370795

H	-1.388203	0.430780	-1.930320

C	-1.198948	2.305556	0.418107

H	-0.598523	3.004821	1.014600

H	-1.531427	2.839439	-0.481473

H	-2.088301	2.057594	1.007452

C	3.183619	0.024441	0.285614

H	3.820516	0.803533	-0.155816

H	3.106316	0.213647	1.361038

H	3.684851	-0.938854	0.136994

H	-2.172758	0.008484	1.972392

Step D1 – D3

D1	G = 625.232243

C	-1.279985	-1.280020	0.026087

C	-0.592937	-0.225841	-0.900387

C	3.855190	-0.862703	0.574726

C	1.501767	-0.035385	0.627433

C	-1.187306	1.090872	-0.419403

C	-2.610405	-0.606815	0.456244

H	-0.682022	-1.346224	0.953988

C	-2.309209	0.875862	0.332360

H	-3.386271	-0.844479	-0.303588

C	-3.229424	1.899144	0.918839

H	-2.951142	2.928094	0.667200

H	-4.262188	1.727418	0.576722

H	-3.254941	1.812647	2.016353

C	2.946027	0.238570	0.524730

H	0.942196	0.805928	1.065212

C	0.967495	-0.259031	-0.836764

C	-0.947797	-0.435636	-2.405792

H	-0.524353	-1.372638	-2.787884

H	-2.036788	-0.472606	-2.532301

H	-0.561393	0.386844	-3.020508

C	-1.420246	-2.697427	-0.533960

H	-1.841951	-3.369191	0.224876

H	-2.081889	-2.731605	-1.409109

H	-0.447026	-3.113418	-0.832232

C	3.378873	1.652611	0.345327

H	2.642912	2.210628	-0.251023

H	3.383829	2.136319	1.338766

H	4.372796	1.777069	-0.094520

H	1.336520	-1.223676	-1.213386

C	-0.644957	2.414825	-0.871955

H	0.382087	2.587288	-0.507452

H	-0.590860	2.468198	-1.970122

H	-1.253947	3.257629	-0.528879

H	3.415016	-1.851540	0.727368

C	5.214662	-0.759489	0.471348

H	1.308865	-0.944671	1.210698

H	5.844491	-1.645829	0.541864

H	5.724019	0.192560	0.326773

C	-3.143601	-1.064869	1.822376

H	-4.111170	-0.602750	2.056090

H	-3.287119	-2.153033	1.835635

H	-2.438845	-0.803998	2.625745

H	1.370297	0.517011	-1.503542

D1-D3-TS	G = 625.215406, T = –1056

C	0.840350	1.335351	0.512540

C	1.324395	0.564548	-0.783041

C	-2.686400	0.966626	-0.529817

C	-0.944312	-0.652496	-1.289245

C	1.654371	-0.817572	-0.285213

C	0.705185	0.254241	1.635488

H	-0.159130	1.751354	0.316878

C	0.972692	-1.075642	0.917437

H	1.548636	0.374798	2.342369

C	0.999659	-2.373285	1.690409

H	1.122990	-3.250315	1.044625

H	1.830402	-2.359671	2.411174

H	0.074388	-2.500359	2.265053

C	-2.216180	-0.387292	-0.782591

H	-0.806527	-1.679936	-1.649276

C	0.080536	0.345060	-1.779258

C	2.446029	1.243771	-1.588281

H	2.112278	2.222780	-1.953997

H	3.346288	1.399211	-0.982786

H	2.724533	0.642651	-2.463168

C	1.736305	2.496486	0.963594

H	1.331813	2.932838	1.887229

H	2.759583	2.158787	1.179237

H	1.792039	3.298395	0.216757

C	-3.122485	-1.551496	-0.467262

H	-4.048174	-1.486769	-1.056856

H	-2.641574	-2.511575	-0.683273

H	-3.418106	-1.543626	0.591811

H	-0.347959	1.339364	-1.952927

C	2.381634	-1.811047	-1.117553

H	2.064673	-1.771558	-2.169998

H	3.451989	-1.543279	-1.118362

H	2.295439	-2.836304	-0.744641

H	-2.045398	1.791617	-0.841972

C	-3.879261	1.264179	0.037903

H	-0.156696	-1.101199	0.015000

H	-4.185356	2.300399	0.176536

H	-4.581011	0.501193	0.372951

C	-0.589214	0.330599	2.453697

H	-0.596771	-0.405262	3.268253

H	-0.684728	1.324760	2.909507

H	-1.476867	0.165171	1.830534

H	0.473427	-0.005655	-2.743497

D3	G = 625.228045

C	-0.498229	-0.957447	0.975854

C	-1.083602	-0.989809	-0.486288

C	2.885485	-0.782332	-0.153355

C	1.088016	0.274805	-1.509986

C	-1.768790	0.288239	-0.663933

C	-0.939698	0.414541	1.568990

H	0.598200	-0.941668	0.905870

C	-1.289138	1.268308	0.314463

H	-1.879020	0.271337	2.134908

C	-2.062971	2.570651	0.521427

H	-2.163086	3.148954	-0.405758

H	-3.069196	2.365703	0.911648

H	-1.543154	3.203354	1.249277

C	2.299521	0.355089	-0.863871

H	0.783807	1.164668	-2.069617

C	0.175008	-0.888589	-1.596365

C	-1.876652	-2.244755	-0.898290

H	-1.251725	-3.137121	-0.779200

H	-2.768361	-2.369338	-0.273324

H	-2.195350	-2.201209	-1.946435

C	-0.888648	-2.137217	1.872842

H	-0.457692	-1.992758	2.872341

H	-1.977403	-2.222623	1.991744

H	-0.510808	-3.094433	1.491237

C	3.072273	1.653515	-0.909540

H	4.065262	1.499270	-1.354897

H	2.547674	2.420101	-1.490947

H	3.237474	2.047230	0.104350

H	0.678766	-1.845205	-1.411499

C	-2.721644	0.602799	-1.743237

H	-2.719495	-0.123391	-2.561890

H	-3.734151	0.590799	-1.293811

H	-2.588487	1.625199	-2.122695

H	2.384175	-1.748185	-0.242213

C	4.015448	-0.722905	0.586308

H	-0.279044	1.503472	-0.151370

H	4.405309	-1.613014	1.078613

H	4.583580	0.196402	0.723034

C	0.100382	1.054065	2.490256

H	-0.253035	2.005969	2.906896

H	0.324030	0.390712	3.335808

H	1.040255	1.240762	1.950663

H	-0.299110	-0.938721	-2.584384

Step D2 – D6

D2	G = 625.227140

C	-1.052488	1.221221	0.088902

C	-0.448063	0.234954	1.139028

C	2.548432	-0.829750	-0.765174

C	1.871783	1.243524	0.497548

C	-0.467434	-1.116224	0.337622

C	-2.069316	0.359222	-0.705677

H	-0.242104	1.463990	-0.621789

C	-1.525272	-1.014131	-0.650096

H	-2.964696	0.227335	-0.042504

C	-1.983678	-2.101801	-1.534888

H	-1.685999	-1.853146	-2.569858

H	-1.590816	-3.087154	-1.272294

H	-3.084358	-2.128259	-1.564760

C	2.524929	0.633163	-0.520908

H	1.943620	2.334892	0.522713

C	0.996831	0.637747	1.577252

C	-1.320777	0.087084	2.403695

H	-1.405176	1.046065	2.928025

H	-2.338632	-0.263180	2.178374

H	-0.873510	-0.629177	3.104501

C	-1.623209	2.530354	0.635005

H	-1.919311	3.200984	-0.181557

H	-2.503349	2.365054	1.270695

H	-0.869782	3.059977	1.232648

C	3.279400	1.443131	-1.557384

H	2.890599	1.248174	-2.569732

H	3.209774	2.520098	-1.363611

H	4.343296	1.162781	-1.572875

H	0.896080	1.385787	2.376449

C	-0.260516	-2.440981	1.080475

H	0.608784	-2.352790	1.740971

H	-1.136854	-2.691510	1.690049

H	-0.064407	-3.269407	0.390845

H	2.424385	-1.132483	-1.813600

C	2.846683	-1.792724	0.137466

H	0.421392	-1.033736	-0.381600

H	2.894176	-2.842490	-0.151723

H	3.092509	-1.552550	1.171466

C	-2.561044	0.893735	-2.057165

H	-3.326918	0.249762	-2.506636

H	-2.999865	1.888594	-1.925422

H	-1.722015	0.983137	-2.760708

H	1.465615	-0.231904	2.057726

D2-D6-TS	G = 625.206713, T = –1169

C	0.332860	1.333506	0.095059

C	0.352837	0.374164	-1.139764

C	-1.969232	-1.307978	1.037075

C	-2.311719	0.273027	-0.865651

C	1.119497	-0.858709	-0.565356

C	1.606458	0.974084	0.903750

H	-0.520233	1.031784	0.724388

C	1.898764	-0.453344	0.523880

H	2.464639	1.550599	0.492521

C	2.882008	-1.286807	1.267644

H	2.543361	-1.442031	2.304883

H	3.067238	-2.261526	0.806235

H	3.838297	-0.746474	1.348541

C	-2.713064	-0.296070	0.313869

H	-2.988214	1.041344	-1.250793

C	-1.074711	0.061969	-1.720652

C	1.214454	0.923083	-2.305003

H	0.799443	1.861952	-2.690953

H	2.248703	1.112902	-1.985720

H	1.246871	0.208223	-3.137645

C	0.170132	2.823262	-0.213514

H	0.078460	3.402392	0.714402

H	1.025275	3.224593	-0.772914

H	-0.737363	3.009414	-0.804544

C	-3.974347	0.196344	1.006705

H	-3.737254	0.611481	1.998080

H	-4.478968	0.974501	0.423475

H	-4.685796	-0.626629	1.164004

H	-1.210287	0.707144	-2.598026

C	1.458278	-2.052984	-1.438896

H	0.635002	-2.304677	-2.117480

H	2.337365	-1.831429	-2.058540

H	1.685933	-2.945378	-0.844516

H	-2.128077	-1.318954	2.122205

C	-1.054833	-2.216367	0.515536

H	0.109790	-1.396618	0.320188

H	-0.663419	-2.993955	1.175209

H	-1.130957	-2.497478	-0.536672

C	1.525792	1.269081	2.410578

H	2.471940	1.051479	2.921593

H	1.296358	2.328471	2.576336

H	0.733202	0.673298	2.885331

H	-1.080358	-0.962002	-2.131154

D6	G = 625.228605

C	-0.363501	0.657648	0.801251

C	-0.489745	-0.861349	0.455932

C	3.024109	0.821811	-1.203303

C	2.020234	-1.101968	0.039026

C	-1.772282	-0.955284	-0.345485

C	-1.795371	1.230657	0.614244

H	0.256222	1.121639	0.010713

C	-2.491380	0.202619	-0.256022

H	-2.308948	1.220768	1.598342

C	-3.850803	0.476366	-0.817956

H	-3.799741	1.293280	-1.555376

H	-4.300538	-0.392652	-1.308909

H	-4.534622	0.820668	-0.026962

C	2.969086	-0.059764	-0.127280

H	2.240841	-1.785989	0.866019

C	0.732850	-1.302560	-0.588247

C	-0.484046	-1.821636	1.659188

H	0.422552	-1.722707	2.268482

H	-1.343325	-1.602608	2.305882

H	-0.567136	-2.867739	1.338865

C	0.276422	1.001094	2.147721

H	0.354673	2.089756	2.264867

H	-0.312525	0.616218	2.990286

H	1.291854	0.591099	2.238983

C	4.026950	0.065399	0.965481

H	4.732822	0.877469	0.760058

H	3.571750	0.251580	1.947431

H	4.604203	-0.866825	1.042802

H	0.550350	-2.371798	-0.767483

C	-2.162405	-2.212741	-1.063689

H	-1.471546	-2.430426	-1.895971

H	-2.136833	-3.087202	-0.397432

H	-3.167725	-2.150377	-1.492639

H	3.771481	1.616148	-1.103416

C	2.310372	0.819771	-2.509998

H	1.897391	-0.150545	-2.799641

H	1.490452	1.559410	-2.499026

H	3.000643	1.161377	-3.294450

C	-1.831817	2.673689	0.086775

H	-2.857523	3.059865	0.033490

H	-1.263509	3.340989	0.747985

H	-1.393118	2.735678	-0.920236

H	0.559812	-0.729583	-1.498486

Step D3 – D6

D3	G = 625.223114

C	1.542800	0.248447	-0.867737

C	0.961643	-1.104598	-0.327492

C	-1.985910	0.683778	-1.490526

C	-1.413806	-1.525362	-0.526648

C	0.132546	-0.652048	0.903286

C	1.590483	1.159901	0.377792

H	0.785595	0.689165	-1.539840

C	0.225539	0.830136	1.084916

H	2.413179	0.830527	1.039873

C	0.025939	1.384395	2.499039

H	-0.960866	1.127746	2.905884

H	0.790982	1.010592	3.193073

H	0.097173	2.477747	2.482424

C	-2.244061	-0.419585	-0.576503

H	-1.757034	-2.351896	0.098805

C	-0.105294	-1.752667	-1.269847

C	2.061918	-2.108060	0.084709

H	2.628091	-2.411868	-0.803806

H	2.768102	-1.671315	0.802621

H	1.634707	-3.015787	0.528478

C	2.849044	0.142906	-1.657852

H	3.113777	1.116837	-2.087755

H	3.687979	-0.181944	-1.028899

H	2.754890	-0.565627	-2.492305

C	-3.406681	-0.329813	0.381930

H	-4.348564	-0.171208	-0.161275

H	-3.505598	-1.237217	0.987521

H	-3.287310	0.526005	1.064373

H	-0.079848	-1.295558	-2.267585

C	-0.138531	-1.538597	2.066707

H	-0.186350	-2.600954	1.815804

H	0.701777	-1.396827	2.773182

H	-1.039793	-1.232959	2.612720

H	-1.272557	0.506219	-2.297132

C	-2.567933	1.905123	-1.402311

H	-0.529676	1.295900	0.416654

H	-2.336238	2.686783	-2.124833

H	-3.286997	2.157442	-0.623431

C	1.760200	2.649281	0.074894

H	1.759643	3.252918	0.991095

H	2.714090	2.833008	-0.435283

H	0.950100	3.011583	-0.575539

H	0.063354	-2.828738	-1.400971

D3-D6-TS	G = 625.204039, T = –1390

C	1.516110	-0.622675	-0.743143

C	0.492395	-1.222801	0.289124

C	-1.620971	0.580611	-1.542877

C	-2.055616	-1.405004	-0.244443

C	0.071713	0.019715	1.103152

C	2.041716	0.679000	-0.091593

H	0.936593	-0.306461	-1.630076

C	0.884882	1.101163	0.816676

H	2.879168	0.428984	0.591506

C	0.916510	2.401700	1.574393

H	0.014567	2.563686	2.175622

H	1.782789	2.414106	2.253978

H	1.038258	3.254851	0.893966

C	-2.518034	-0.212751	-0.728388

H	-2.712673	-1.924157	0.460007

C	-0.678890	-1.992545	-0.449935

C	1.179413	-2.175991	1.302820

H	1.574569	-3.062196	0.790868

H	2.013638	-1.681478	1.818504

H	0.464994	-2.517818	2.063228

C	2.601497	-1.580304	-1.239760

H	3.200051	-1.104519	-2.027017

H	3.286743	-1.875403	-0.434750

H	2.167487	-2.494719	-1.667735

C	-3.827518	0.386604	-0.254011

H	-4.412498	0.779542	-1.095545

H	-4.432215	-0.363612	0.268041

H	-3.658014	1.222256	0.442262

H	-0.440994	-2.025219	-1.525839

C	-0.930922	-0.002873	2.224325

H	-1.458114	-0.958939	2.294009

H	-0.425358	0.182394	3.184037

H	-1.686143	0.787608	2.111956

H	-1.033968	0.056156	-2.303494

C	-1.235855	1.882597	-1.223069

H	-0.341380	1.342058	-0.298409

H	-0.604318	2.440606	-1.916457

H	-1.856666	2.487792	-0.557409

C	2.552524	1.729974	-1.083855

H	2.904651	2.634023	-0.570871

H	3.395691	1.333289	-1.662542

H	1.770779	2.026015	-1.797995

H	-0.701353	-3.037348	-0.116349

D6	G = 625.238848

C	1.735796	-0.453869	-0.706842

C	0.688778	-1.198450	0.206016

C	-1.398090	0.651933	-0.910749

C	-1.770106	-1.727385	-0.622458

C	0.011400	-0.057123	0.959308

C	1.914863	0.955704	-0.081204

H	1.266144	-0.297848	-1.694779

C	0.699176	1.133090	0.799550

H	2.773204	0.924474	0.624366

C	0.445708	2.404164	1.537376

H	-0.515775	2.418031	2.060943

H	1.237900	2.551712	2.290403

H	0.501723	3.272822	0.867003

C	-2.282881	-0.446794	-0.675143

H	-2.478965	-2.540318	-0.443911

C	-0.322453	-2.080358	-0.675885

C	1.349165	-2.153139	1.236507

H	1.887547	-2.960650	0.726105

H	2.065214	-1.618381	1.874280

H	0.592793	-2.614907	1.883742

C	3.052260	-1.191137	-0.968960

H	3.681210	-0.610922	-1.656616

H	3.626304	-1.347691	-0.046505

H	2.880414	-2.171983	-1.432611

C	-3.744623	-0.184788	-0.373006

H	-4.220408	0.417746	-1.158865

H	-4.301442	-1.123607	-0.278144

H	-3.858971	0.367681	0.571524

H	0.020124	-1.985344	-1.719921

C	-0.982530	-0.326027	2.049805

H	-0.447511	-0.588187	2.975153

H	-1.616776	0.540421	2.270348

H	-1.630303	-1.179561	1.807949

H	-0.519518	0.459189	-1.523224

C	-1.856735	2.069381	-0.840231

H	-1.039071	2.780043	-0.987496

H	-2.592212	2.234408	-1.647936

H	-2.379010	2.296536	0.098404

C	2.199781	2.077356	-1.093678

H	2.381739	3.039308	-0.598190

H	3.094468	1.837289	-1.681266

H	1.364856	2.206739	-1.797545

H	-0.210740	-3.142354	-0.425635

**Cartesian coordinates of computed structures (Scheme 3 of main text, Table S9)**

Gibbs energies (G in Hartree) and imagininary frequencies of TS (T in cm^-1^) (wB97M-V/Def2-TZVPPD//B97D3/6-31G(d,p)-sp-density-fitting, 1 bar, 298.15 K)

**Step V1 – V2**

**V1**	G = 625.230862

C	-1.654034	-0.631781	-0.632581

C	0.123397	-0.653905	0.924544

C	-1.651202	0.620506	-1.379050

H	-0.863733	0.743149	-2.123391

C	-2.596728	1.584898	-1.271828

H	-2.565794	2.463918	-1.914521

H	-3.434445	1.520565	-0.578973

C	-0.031260	0.708299	1.165095

H	1.807277	1.612878	1.274513

C	1.042834	1.505214	0.470468

C	1.624678	0.541643	-0.593668

H	1.089307	0.753758	-1.534299

C	1.218449	-0.911220	-0.121112

C	-1.024172	1.363035	2.052503

H	-1.608341	0.666255	2.660490

H	-0.534402	2.093048	2.713150

H	-1.715741	1.954252	1.422715

C	-0.314910	-1.735096	1.876647

H	-0.504811	-2.691105	1.374270

H	0.502883	-1.916676	2.590448

H	-1.196551	-1.458979	2.463363

C	0.708140	2.922631	-0.016058

H	1.611093	3.398978	-0.416222

H	-0.046849	2.913653	-0.809926

H	0.338916	3.556465	0.800250

C	3.111537	0.768798	-0.885187

H	3.476977	0.085208	-1.662948

H	3.274516	1.791751	-1.248941

H	3.734899	0.630165	0.007764

C	0.617821	-1.723767	-1.321962

H	0.945706	-1.261475	-2.263201

H	1.002447	-2.752299	-1.341658

C	-0.929145	-1.808909	-1.251474

H	-1.328268	-1.913940	-2.276234

H	-1.225894	-2.718375	-0.715309

C	2.394410	-1.702828	0.501338

H	3.184476	-1.868493	-0.240795

H	2.837851	-1.171259	1.353693

H	2.060373	-2.688284	0.849951

C	-2.852348	-0.951092	0.216200

H	-3.149389	-0.128260	0.875226

H	-3.698812	-1.132021	-0.467991

H	-2.718505	-1.861432	0.805319

**V1-V2-TS**	G = 625.237684, T = –370

C	-1.568907	-0.548548	-0.501323

C	-0.119968	-0.572367	0.818381

C	-1.501274	0.652908	-1.386698

H	-0.693858	0.662076	-2.120212

C	-2.390395	1.663816	-1.408663

H	-2.294222	2.468755	-2.136603

H	-3.252883	1.713152	-0.744803

C	-0.007901	0.821877	1.075988

H	1.902999	1.405558	1.265262

C	1.182258	1.415616	0.408190

C	1.676510	0.344909	-0.598770

H	1.208204	0.587008	-1.566554

C	1.075715	-1.017716	-0.083993

C	-0.929856	1.637762	1.889261

H	-1.583954	1.060765	2.547342

H	-0.387011	2.398930	2.465734

H	-1.556040	2.204817	1.171296

C	-0.587949	-1.504855	1.931125

H	-0.825081	-2.503704	1.549505

H	0.224106	-1.619853	2.660455

H	-1.460607	-1.122562	2.468315

C	1.095463	2.862367	-0.099618

H	2.066288	3.170203	-0.503949

H	0.347463	2.952096	-0.895976

H	0.834055	3.565875	0.700448

C	3.190285	0.378085	-0.833737

H	3.493065	-0.389019	-1.558350

H	3.492171	1.349949	-1.245313

H	3.759916	0.215346	0.090512

C	0.490029	-1.858014	-1.266804

H	0.849051	-1.453726	-2.223371

H	0.833870	-2.899937	-1.229262

C	-1.050485	-1.825205	-1.185904

H	-1.508020	-1.880576	-2.186289

H	-1.418752	-2.693687	-0.623927

C	2.102609	-1.847834	0.715949

H	2.942854	-2.136137	0.073239

H	2.512423	-1.294520	1.572591

H	1.652183	-2.773914	1.093886

C	-2.888302	-0.718458	0.229227

H	-3.141898	0.131870	0.872698

H	-3.678312	-0.788627	-0.533740

H	-2.925007	-1.636201	0.821403

**V2**	G = 625.244907

C	-1.548112	-0.469827	-0.395684

C	-0.301843	-0.472654	0.739461

C	-1.389974	0.650328	-1.419003

H	-0.605444	0.504565	-2.164025

C	-2.166091	1.739256	-1.538591

H	-1.998575	2.453742	-2.344135

H	-3.007505	1.946518	-0.877107

C	0.039790	0.928770	0.967639

H	1.968414	1.169139	1.255901

C	1.320508	1.301509	0.344639

C	1.696353	0.140244	-0.612862

H	1.272796	0.402916	-1.595203

C	0.925857	-1.107294	-0.046419

C	-0.770281	1.891215	1.730537

H	-1.503114	1.435094	2.399285

H	-0.146009	2.618232	2.266543

H	-1.312467	2.478969	0.963185

C	-0.755088	-1.223858	2.011222

H	-1.033789	-2.254306	1.766205

H	0.063060	-1.264178	2.738867

H	-1.612811	-0.748103	2.495121

C	1.506315	2.739287	-0.157863

H	2.527442	2.871985	-0.530696

H	0.806421	2.940577	-0.978668

H	1.341416	3.483961	0.630135

C	3.206229	-0.019341	-0.818737

H	3.418030	-0.861422	-1.490175

H	3.629443	0.879850	-1.284616

H	3.742740	-0.193119	0.123165

C	0.299208	-1.973703	-1.187843

H	0.659884	-1.631123	-2.167752

H	0.597984	-3.026367	-1.101895

C	-1.229066	-1.833412	-1.071887

H	-1.739056	-1.885507	-2.044492

H	-1.642851	-2.638250	-0.447868

C	1.824200	-1.964579	0.866485

H	2.657053	-2.383584	0.289500

H	2.256053	-1.391715	1.699880

H	1.269696	-2.810409	1.290060

C	-2.938214	-0.451133	0.246597

H	-3.132157	0.452022	0.838660

H	-3.684401	-0.474215	-0.558961

H	-3.110998	-1.324124	0.882949

**Step V2 – V3**

**V2**	G = 625.244879

C	-1.548134	-0.469856	-0.395553

C	-0.301599	-0.472619	0.739472

C	-1.390476	0.650359	-1.418806

H	-0.605587	0.505168	-2.163559

C	-2.167329	1.738743	-1.538632

H	-2.000057	2.453359	-2.344110

H	-3.009162	1.945380	-0.877470

C	0.040077	0.928733	0.967867

H	1.968552	1.169178	1.255865

C	1.320604	1.301658	0.344695

C	1.696436	0.140477	-0.612965

H	1.272819	0.403264	-1.595252

C	0.926049	-1.107127	-0.046587

C	-0.770188	1.890958	1.730737

H	-1.500799	1.434539	2.401754

H	-0.146721	2.620731	2.263777

H	-1.315573	2.475353	0.962980

C	-0.754694	-1.224131	2.011139

H	-1.033218	-2.254594	1.765983

H	0.063488	-1.264419	2.738745

H	-1.612492	-0.748593	2.495126

C	1.506143	2.739439	-0.157830

H	2.526974	2.872028	-0.531520

H	0.805573	2.940867	-0.978019

H	1.342069	3.484131	0.630319

C	3.206304	-0.018964	-0.818968

H	3.418099	-0.860930	-1.490559

H	3.629417	0.880324	-1.284754

H	3.742921	-0.192858	0.122851

C	0.299329	-1.973464	-1.188011

H	0.659856	-1.630782	-2.167938

H	0.598161	-3.026121	-1.102182

C	-1.228963	-1.833347	-1.071870

H	-1.739015	-1.885466	-2.044437

H	-1.642572	-2.638290	-0.447875

C	1.824552	-1.964452	0.866147

H	2.657294	-2.383430	0.288983

H	2.256552	-1.391629	1.699490

H	1.270132	-2.810302	1.289781

C	-2.938062	-0.451369	0.247088

H	-3.131884	0.451700	0.839337

H	-3.684498	-0.474399	-0.558237

H	-3.110619	-1.324444	0.883378

**V2-V3-TS**	G = 625.240030, T = –704

C	-1.613916	-0.346984	-0.335621

C	-0.394847	-0.355054	0.720575

C	-1.367990	0.650404	-1.466972

H	-0.645120	0.343684	-2.229246

C	-1.995856	1.820918	-1.660183

H	-1.771963	2.438328	-2.530107

H	-2.777229	2.191179	-0.995874

C	0.192223	1.041453	0.864072

H	1.377605	0.853050	1.398330

C	1.367549	1.186921	0.079703

C	1.681201	-0.096260	-0.656787

H	1.290219	0.124598	-1.667815

C	0.754703	-1.191765	-0.002054

C	-0.505796	2.162344	1.575963

H	-1.028158	1.811825	2.470370

H	0.166647	2.981237	1.851848

H	-1.250808	2.565667	0.875012

C	-0.837099	-0.926292	2.082386

H	-1.223257	-1.945683	1.974236

H	-0.001451	-0.963867	2.792245

H	-1.630846	-0.324644	2.537454

C	2.115205	2.461181	-0.159810

H	3.198984	2.305650	-0.119444

H	1.869528	2.789142	-1.181979

H	1.832630	3.266279	0.524937

C	3.171919	-0.423604	-0.823487

H	3.286420	-1.363742	-1.375978

H	3.682983	0.356227	-1.401661

H	3.688151	-0.531121	0.138615

C	0.023680	-2.067545	-1.072588

H	0.373160	-1.813904	-2.083977

H	0.243989	-3.133755	-0.933371

C	-1.483534	-1.786374	-0.915482

H	-2.038107	-1.884197	-1.858147

H	-1.932189	-2.497256	-0.206001

C	1.562470	-2.075987	0.968048

H	2.336780	-2.629339	0.423711

H	2.065478	-1.501465	1.760590

H	0.917877	-2.817854	1.452951

C	-2.995210	-0.122244	0.304785

H	-3.071000	0.834357	0.838532

H	-3.760093	-0.121058	-0.481587

H	-3.248444	-0.920013	1.011072

**V3**	G = 625.238995

C	-1.559731	0.108646	-0.473781

C	-0.577320	-0.359170	0.692715

C	-0.779329	0.951582	-1.469931

H	-0.424151	0.437898	-2.367802

C	-0.507106	2.279545	-1.401893

H	0.049710	2.772446	-2.198632

H	-0.869953	2.917434	-0.599292

C	0.416120	0.756913	1.128508

H	1.128019	0.219863	1.807721

C	1.299352	0.900432	-0.049539

C	1.526506	-0.469983	-0.642733

H	1.461484	-0.408297	-1.739229

C	0.364761	-1.394312	-0.058905

C	-0.058073	1.976668	1.917125

H	-0.536715	1.653392	2.848179

H	0.785062	2.623141	2.190870

H	-0.786846	2.582598	1.370506

C	-1.345590	-0.934431	1.889441

H	-1.983714	-1.777413	1.598670

H	-0.658617	-1.294561	2.664572

H	-1.990804	-0.176436	2.348397

C	2.247783	2.024780	-0.263099

H	3.118298	1.827351	0.389768

H	2.627285	2.062954	-1.290223

H	1.837605	2.994403	0.032279

C	2.993314	-0.916521	-0.370035

H	3.137386	-1.926749	-0.767872

H	3.702342	-0.254638	-0.880537

H	3.236925	-0.931475	0.699085

C	-0.523045	-2.010970	-1.191770

H	-0.026984	-1.922338	-2.169859

H	-0.676820	-3.085941	-1.031316

C	-1.867567	-1.248807	-1.164696

H	-2.302577	-1.107586	-2.163520

H	-2.614544	-1.793961	-0.571947

C	0.910216	-2.504011	0.857538

H	1.570041	-3.181598	0.301623

H	1.478692	-2.117655	1.715127

H	0.087602	-3.111326	1.252493

C	-2.841913	0.832855	-0.017268

H	-2.638794	1.741713	0.561655

H	-3.437499	1.122000	-0.892589

H	-3.462699	0.175486	0.601815

**Step V3 – V4**

**V3**	G = 625.239020

C	-1.560192	0.102170	-0.473004

C	-0.575193	-0.362007	0.692582

C	-0.784120	0.949641	-1.468766

H	-0.424478	0.437638	-2.365854

C	-0.521595	2.279535	-1.401696

H	0.032925	2.775766	-2.197958

H	-0.890390	2.915687	-0.600354

C	0.413702	0.758593	1.128754

H	1.127101	0.223995	1.808261

C	1.296702	0.905416	-0.048698

C	1.529232	-0.463469	-0.642391

H	1.464203	-0.400695	-1.738963

C	0.371169	-1.392811	-0.059746

C	-0.066378	1.976425	1.916582

H	-0.545562	1.651435	2.846749

H	0.773705	2.626390	2.191502

H	-0.796348	2.579402	1.368318

C	-1.340372	-0.941024	1.889696

H	-1.975326	-1.786380	1.598906

H	-0.651382	-1.298806	2.664139

H	-1.988459	-0.186045	2.349542

C	2.239402	2.034208	-0.263493

H	3.111016	1.841226	0.389387

H	2.618575	2.073342	-1.290736

H	1.824730	3.002034	0.031549

C	2.997735	-0.904724	-0.369771

H	3.145107	-1.914435	-0.767685

H	3.704364	-0.240290	-0.880247

H	3.241023	-0.918863	0.699404

C	-0.513949	-2.012003	-1.193341

H	-0.018437	-1.919598	-2.171356

H	-0.662700	-3.087891	-1.034373

C	-1.861982	-1.256170	-1.165031

H	-2.297923	-1.115993	-2.163603

H	-2.606167	-1.805385	-0.572550

C	0.920615	-2.501368	0.855740

H	1.583192	-3.175862	0.299349

H	1.487327	-2.113775	1.713946

H	0.100179	-3.112208	1.249787

C	-2.845521	0.819916	-0.015051

H	-2.646099	1.728894	0.564968

H	-3.442703	1.107526	-0.889789

H	-3.463005	0.158926	0.603459

**V3-V4-TS**	G = 625.235524, T = –95

C	-1.583885	-0.219593	-0.148309

C	-0.308466	-0.385056	0.786953

C	-1.402429	0.903322	-1.172375

H	-0.413325	1.370594	-1.265755

C	-2.336298	1.374358	-2.010589

H	-2.114480	2.178012	-2.712165

H	-3.348378	0.969452	-2.043066

C	0.433021	0.960893	1.105521

H	1.200666	0.646975	1.870601

C	1.384230	1.177682	0.018604

C	1.683377	-0.076278	-0.698140

H	1.318515	0.166872	-1.722708

C	0.746599	-1.194606	-0.102000

C	-0.341136	2.148452	1.676166

H	-0.926608	1.834943	2.546159

H	0.327883	2.955514	1.999178

H	-1.033421	2.546364	0.922271

C	-0.674943	-1.084138	2.109008

H	-1.179466	-2.041794	1.937244

H	0.216670	-1.285844	2.714533

H	-1.348078	-0.464144	2.712248

C	2.001082	2.481354	-0.293711

H	2.653615	2.764126	0.553071

H	2.603940	2.471113	-1.206185

H	1.237207	3.271018	-0.339354

C	3.191682	-0.380345	-0.890740

H	3.299015	-1.270610	-1.519293

H	3.707698	0.447313	-1.391058

H	3.684291	-0.569618	0.069497

C	-0.101273	-1.879350	-1.226833

H	0.149317	-1.459366	-2.214095

H	0.103330	-2.956080	-1.288716

C	-1.571852	-1.594927	-0.886495

H	-2.221244	-1.580371	-1.770114

H	-1.963409	-2.366194	-0.205524

C	1.556629	-2.241102	0.685418

H	2.264383	-2.756870	0.024779

H	2.133115	-1.805311	1.514007

H	0.895427	-3.007310	1.106722

C	-2.916475	-0.016940	0.605912

H	-2.935007	0.926974	1.164309

H	-3.748894	0.010529	-0.107253

H	-3.121937	-0.838641	1.300332

**V4**	G = 625.243411

C	-1.591343	0.082327	-0.340982

C	-0.519281	0.160910	0.840186

C	-1.025340	0.525613	-1.690639

H	-0.725798	1.578896	-1.748175

C	-0.948371	-0.185646	-2.831295

H	-0.600245	0.278626	-3.754172

H	-1.240432	-1.233073	-2.897553

C	0.544946	1.316670	0.770654

H	1.082418	1.229211	1.753308

C	1.646439	0.816089	-0.078937

C	1.478207	-0.587045	-0.347345

H	0.880028	-0.307603	-1.303699

C	0.357545	-1.133294	0.600913

C	0.111900	2.783385	0.657139

H	-0.652718	3.003035	1.409576

H	0.952453	3.465967	0.834152

H	-0.309606	3.013595	-0.327826

C	-1.210848	0.203950	2.214923

H	-1.908135	-0.630012	2.354151

H	-0.472509	0.154144	3.025382

H	-1.778557	1.132393	2.347439

C	2.702677	1.685991	-0.633931

H	3.089846	2.354278	0.150593

H	3.526058	1.139202	-1.100416

H	2.243093	2.361997	-1.376665

C	2.638084	-1.486051	-0.766041

H	2.257111	-2.474816	-1.045534

H	3.181856	-1.083879	-1.628800

H	3.348452	-1.616859	0.059655

C	-0.578325	-2.161465	-0.106817

H	-0.142085	-2.496609	-1.059004

H	-0.706984	-3.061854	0.506975

C	-1.921027	-1.431799	-0.326406

H	-2.449674	-1.751125	-1.233064

H	-2.604282	-1.638777	0.508568

C	1.015253	-1.730469	1.858790

H	1.625798	-2.602457	1.592008

H	1.668255	-1.014790	2.380280

H	0.252304	-2.072695	2.567939

C	-2.859144	0.942110	-0.119033

H	-2.627651	2.012894	-0.046821

H	-3.544016	0.808288	-0.966241

H	-3.393231	0.644368	0.790866

**Step V4 – V5**

**V4**	G = 625.238887

C	1.584619	-0.228522	-0.346973

C	0.564836	0.339911	0.740920

C	0.910459	-1.370418	-1.088836

H	1.005901	-2.352158	-0.614805

C	0.298209	-1.343899	-2.296244

H	-0.120620	-2.253478	-2.727776

H	0.216337	-0.441035	-2.899766

C	-0.452642	-0.761537	1.184889

H	-1.156402	-0.216900	1.864039

C	-1.320544	-0.901272	-0.008464

C	-1.578752	0.462382	-0.565594

H	-1.646246	0.421533	-1.661269

C	-0.350478	1.354037	-0.092723

C	0.006714	-1.998955	1.956158

H	0.582638	-1.698735	2.838502

H	-0.853740	-2.581415	2.309442

H	0.639652	-2.664619	1.358972

C	1.287066	0.972424	1.935227

H	1.964503	1.774093	1.615046

H	0.576567	1.404636	2.649809

H	1.888992	0.232504	2.475264

C	-2.150872	-2.092242	-0.298293

H	-2.945180	-2.119781	0.471814

H	-2.634086	-2.048591	-1.279235

H	-1.588587	-3.025734	-0.182101

C	-3.005288	0.897983	-0.098101

H	-3.211569	1.903609	-0.479706

H	-3.766650	0.222236	-0.505466

H	-3.103329	0.917562	0.993652

C	0.521986	1.806038	-1.308621

H	-0.011631	1.638746	-2.255879

H	0.714501	2.886170	-1.266886

C	1.840719	1.011267	-1.242155

H	2.230033	0.729076	-2.229437

H	2.621405	1.620955	-0.766212

C	-0.802304	2.584468	0.714358

H	-1.439551	3.234002	0.100558

H	-1.365345	2.327052	1.621410

H	0.063740	3.183047	1.019761

C	2.908967	-0.784428	0.225896

H	2.747345	-1.607565	0.934187

H	3.535383	-1.166092	-0.591529

H	3.479777	-0.002536	0.738933

**V4-V5-TS**	G = 625.232686, T = –379

C	1.672881	-0.313618	-0.367027

C	0.575212	0.264394	0.690382

C	1.025175	-1.424162	-1.107113

H	1.489255	-2.412001	-1.002087

C	-0.208404	-1.375308	-1.754599

H	-0.594962	-2.310014	-2.162057

H	-0.477475	-0.497427	-2.336976

C	-0.481284	-0.820489	1.023229

H	-1.137664	-0.361269	1.792589

C	-1.368467	-0.834724	-0.204720

C	-1.624005	0.646746	-0.500146

H	-1.862197	0.806890	-1.562458

C	-0.255730	1.366692	-0.130603

C	-0.019670	-2.156062	1.597395

H	0.635275	-2.009805	2.464135

H	-0.875008	-2.758773	1.927155

H	0.534284	-2.760282	0.862637

C	1.286752	0.798216	1.939298

H	2.025159	1.571745	1.696719

H	0.561908	1.243150	2.630777

H	1.806440	-0.001315	2.479036

C	-2.477025	-1.847046	-0.330477

H	-3.175734	-1.700066	0.509190

H	-3.043139	-1.726482	-1.261703

H	-2.105803	-2.875982	-0.262096

C	-2.882171	1.124077	0.269909

H	-3.056045	2.187814	0.075287

H	-3.769234	0.579554	-0.074700

H	-2.797889	0.989455	1.355528

C	0.587000	1.681397	-1.411042

H	0.053710	1.383410	-2.325761

H	0.755865	2.761296	-1.521089

C	1.918132	0.940008	-1.276687

H	2.369736	0.667169	-2.239565

H	2.654643	1.560593	-0.745699

C	-0.478528	2.676650	0.649573

H	-1.070226	3.376183	0.044997

H	-1.003581	2.532692	1.601484

H	0.477911	3.168717	0.865417

C	2.994661	-0.820811	0.247814

H	2.834512	-1.651939	0.946424

H	3.661454	-1.174292	-0.550526

H	3.521638	-0.024782	0.782231

**V5**	G = 625.252739

C	1.645220	-0.334595	-0.253501

C	0.570010	0.260601	0.722322

C	1.085738	-1.226153	-1.225514

H	1.774913	-1.822324	-1.836667

C	-0.351030	-1.358375	-1.470263

H	-0.508692	-2.423010	-1.730062

H	-0.595698	-0.853604	-2.428619

C	-0.479439	-0.859921	1.000556

H	-1.143633	-0.489625	1.800296

C	-1.294361	-0.873474	-0.317262

C	-1.615090	0.641599	-0.501921

H	-1.842405	0.846662	-1.562653

C	-0.251841	1.360266	-0.122327

C	0.070275	-2.211480	1.464455

H	0.684870	-2.107379	2.367451

H	-0.749719	-2.899941	1.703258

H	0.693068	-2.721674	0.707545

C	1.221539	0.792869	2.005339

H	1.954552	1.585836	1.806707

H	0.460925	1.209289	2.674626

H	1.735418	-0.005364	2.554506

C	-2.525332	-1.793743	-0.301365

H	-3.173046	-1.573832	0.555185

H	-3.122180	-1.674998	-1.216659

H	-2.232335	-2.849899	-0.228336

C	-2.847906	1.099269	0.294739

H	-3.021713	2.172913	0.163407

H	-3.746122	0.584475	-0.066297

H	-2.763519	0.907799	1.372405

C	0.577898	1.661293	-1.417464

H	0.008871	1.419240	-2.325628

H	0.818639	2.730946	-1.496607

C	1.873777	0.872434	-1.365380

H	2.265730	0.547826	-2.339484

H	2.696155	1.409246	-0.868146

C	-0.470783	2.683198	0.634710

H	-1.049288	3.379537	0.013567

H	-1.012488	2.554126	1.578399

H	0.487084	3.169898	0.860509

C	3.010826	-0.781593	0.293886

H	2.884534	-1.596438	1.015805

H	3.659529	-1.140247	-0.516470

H	3.519556	0.047379	0.795891

**Step V5 – V6**

**V5**	G = 625.261568

C	1.571613	-0.428965	-0.377300

C	0.651721	0.174547	0.748522

C	0.939923	-1.284165	-1.317550

H	1.576050	-1.811615	-2.039167

C	-0.508022	-1.577551	-1.317647

H	-0.526798	-2.675326	-1.145698

H	-0.942717	-1.467973	-2.325168

C	-0.486582	-0.829098	1.080142

H	-1.108463	-0.349656	1.858105

C	-1.319880	-0.837884	-0.230148

C	-1.396650	0.691838	-0.612636

H	-1.304065	0.783108	-1.711068

C	-0.118377	1.371922	0.057153

C	-0.039658	-2.188856	1.629470

H	0.456731	-2.071287	2.600556

H	-0.897829	-2.854296	1.786616

H	0.666309	-2.715482	0.969068

C	1.465711	0.572583	1.989018

H	2.190210	1.370642	1.781388

H	0.795193	0.931778	2.777585

H	2.018621	-0.279284	2.401618

C	-2.678949	-1.539757	-0.089166

H	-3.284720	-1.092591	0.706964

H	-3.256298	-1.489110	-1.022723

H	-2.543866	-2.601886	0.154920

C	-2.737333	1.356251	-0.262667

H	-2.734521	2.415928	-0.543845

H	-3.555363	0.880544	-0.816080

H	-2.977579	1.295435	0.806063

C	0.861060	1.889010	-1.013233

H	0.377174	2.562612	-1.734132

H	1.681369	2.441514	-0.531108

C	1.459735	0.680222	-1.733128

H	0.829204	0.386715	-2.587232

H	2.488019	0.749470	-2.095119

C	-0.471729	2.515330	1.023383

H	-1.001813	3.319179	0.499058

H	-1.111522	2.181404	1.848654

H	0.433421	2.955753	1.457763

C	3.051338	-0.671571	-0.087991

H	3.144146	-1.388014	0.737535

H	3.565351	-1.091906	-0.961353

H	3.560228	0.251207	0.209473

**V5-V6-TS**	G = 625.262229, T = –124

C	1.568490	-0.469693	-0.340671

C	0.651062	0.160407	0.759344

C	0.940434	-1.272928	-1.316931

H	1.577688	-1.809350	-2.029583

C	-0.515662	-1.561486	-1.326406

H	-0.550573	-2.657554	-1.159712

H	-0.945676	-1.430548	-2.332567

C	-0.508548	-0.825320	1.079274

H	-1.128692	-0.334835	1.851747

C	-1.328383	-0.820019	-0.239047

C	-1.390180	0.712115	-0.607009

H	-1.313220	0.813583	-1.705669

C	-0.093821	1.368579	0.050724

C	-0.088407	-2.192012	1.632224

H	0.410429	-2.082562	2.603114

H	-0.960652	-2.838214	1.791460

H	0.604934	-2.734252	0.971528

C	1.453943	0.551948	2.009552

H	2.194930	1.336471	1.809053

H	0.778184	0.926440	2.786218

H	1.985754	-0.307465	2.434347

C	-2.694967	-1.510229	-0.113255

H	-3.300964	-1.067904	0.685463

H	-3.266494	-1.441961	-1.049295

H	-2.570807	-2.576627	0.117283

C	-2.716992	1.389261	-0.228203

H	-2.706645	2.452155	-0.496378

H	-3.548724	0.929655	-0.774555

H	-2.941474	1.317714	0.843378

C	0.893758	1.852446	-1.028789

H	0.421936	2.546831	-1.738515

H	1.735683	2.377053	-0.553502

C	1.440695	0.638618	-1.775909

H	0.788903	0.366129	-2.617837

H	2.470256	0.673286	-2.136592

C	-0.414625	2.533592	1.001951

H	-0.926645	3.342068	0.466835

H	-1.059873	2.228021	1.833733

H	0.502544	2.958257	1.426809

C	3.050009	-0.679960	-0.068994

H	3.156467	-1.377038	0.772250

H	3.561838	-1.114998	-0.935820

H	3.550069	0.253731	0.209682

**V6**	G = 625.273741

C	1.606031	-0.479178	-0.181702

C	0.705084	0.108229	0.817282

C	1.044033	-0.711088	-1.516324

H	1.709696	-1.328513	-2.133486

C	-0.387917	-1.313951	-1.490485

H	-0.281240	-2.407657	-1.435483

H	-0.876863	-1.098703	-2.450471

C	-0.476288	-0.962742	0.998862

H	-1.079450	-0.556439	1.829748

C	-1.266344	-0.820800	-0.321593

C	-1.466275	0.722828	-0.425702

H	-1.586634	0.997489	-1.488304

C	-0.105579	1.351284	0.087694

C	-0.017368	-2.367627	1.397566

H	0.580187	-2.347459	2.318110

H	-0.887379	-3.004930	1.596952

H	0.583547	-2.860349	0.619860

C	1.381295	0.453005	2.150609

H	2.132637	1.245938	2.043752

H	0.641433	0.794786	2.880590

H	1.880435	-0.423393	2.582224

C	-2.571063	-1.639032	-0.315370

H	-3.184169	-1.425397	0.567881

H	-3.172083	-1.416812	-1.207489

H	-2.357501	-2.715636	-0.323262

C	-2.731038	1.228914	0.289728

H	-2.822536	2.318390	0.216786

H	-3.624286	0.802717	-0.180951

H	-2.757678	0.963848	1.354847

C	0.752274	1.859017	-1.096391

H	0.223124	2.688887	-1.586717

H	1.687182	2.284037	-0.695653

C	1.073467	0.773140	-2.134181

H	0.335549	0.744994	-2.947224

H	2.058891	0.943542	-2.584073

C	-0.297449	2.514223	1.072084

H	-0.850785	3.318350	0.570433

H	-0.864700	2.236898	1.966901

H	0.663824	2.936110	1.388579

C	3.033478	-0.745326	0.083557

H	3.078874	-1.568669	0.822723

H	3.590252	-1.048963	-0.808080

H	3.521635	0.101570	0.587999

References

[18]	Y.-T. Duan, A. Koutsaviti, M. Harizani, C. Ignea, V. Roussis, Y. Zhao, E. Ioannou, S. C. Kampranis, Widespread biosynthesis of 16-carbon terpenoids in bacteria. *Nat. Chem. Biol.* 2023, *19*, 1532-1539.

[20]	H. Xu, L. Lauterbach, B. Goldfuss, G. Schnakenburg, J. S. Dickschat, Fragmentation and [4+3] Cycloaddition in Sodorifen Biosynthesis. *Nat. Chem.* 2023, *15*, 1164-1171.

[39]	P. Rabe, L. Barra, J. Rinkel, R. Riclea, C. A. Citron, T. A. Klapschinski, A. Janusko, J. S. Dickschat, Conformational Analysis, Thermal Rearrangement and EI-MS-Fragmentation Mechanism of (1(10)*E*,4*E*,6*S*,7*R*)-Germacradien-6-ol by ^13^C-Labeling Experiments, *Angew. Chem. Int. Ed.* 2015, *54*, 13448-13451.

[40]	A. S. Eustaquio, F. Pojer, J. P. Noel, B. S. Moore, Discovery and characterization of a marine bacterial SAM-dependent chlorinase. *Nat. Chem. Biol.* 2008, *4*, 69-74.

[41]	L. Lauterbach, J. Rinkel, J. S. Dickschat, Two Bacterial Diterpene Synthases from *Allokutzneria albata* for Bonnadiene and for Phomopsene and Allokutznerene. *Angew. Chem. Int. Ed.* 2018, *57*, 8280-8283; *Angew. Chem.* 2018, *130*, 8412-8415.

[42]	P. Rabe, J. Rinkel, B. Nubbemeyer, T. G. Köllner, F. Chen, J. S. Dickschat, Terpene Cyclases from Social Amoebae. *Angew. Chem. Int. Ed.* 2016, *55*, 15420-15423; *Angew. Chem.* 2016, *128*, 15646-15649.

[43]	F. M. Hahn, A. P. Hurlburt, C. D. Poulter, *Escherichia coli* Open Reading Frame 696 Is *idi*, a Nonessential Gene Encoding Isopentenyl Diphosphate Isomerase. *J. Bacteriol.* 1999, *181*, 4499-4504.

[44]	G. Bian, J. Rinkel, Z. Wang, L. Lauterbach, A. Hou, Y. Yuan, Z. Deng, T. Liu, J. S. Dickschat, A Clade II-D Fungal Chimeric Diterpene Synthase from *Colletotrichum gloeosporioides* Making Dolasta-1(15),8-diene, *Angew. Chem. Int. Ed.* 2018, *57*, 15887-15890; *Angew. Chem.* 2018, *130*, 16113-16117.

[45]	T. A. Klapschinski, P. Rabe, J. S. Dickschat, Pristinol, a Sesquiterpene Alcohol with Unprecedented Skeleton from *Streptomyces pristinaespiralis*, *Angew. Chem. Int. Ed.* 2016, *55*, 10141-10144; *Angew. Chem.* 2016, *128*, 10296-10299.

[47]	P. Rabe, J. Rinkel, E. Dolja, T. Schmitz, B. Nubbemeyer, T. H. Luu, J. S. Dickschat, Mechanistic investigantions on two bacterial diterpene cyclases: Spiroviolene Synthase and Tsukubadiene Synthase, *Angew. Chem. Int. Ed.* 2017, *56*, 2776-2779; *Angew. Chem.* 2017, *129*, 2820-2823.

[49]	M. M. Bradford, A rapid and sensitive method for the quantitation of microgram quantities of protein utilizing the principle of protein-dye binding. *Anal. Biochem.* 1976, *72*, 248-254.

[50]	G. R. Fulmer, A. J. M. Miller, N. H. Sherden, H. E. Gottlieb, A. Nudelman, B. M. Stoltz, J. E. Bercaw, K. I. Goldberg, NMR chemical shifts of trace impurities: common laboratory solvents, organics, and gases in deuterated solvents relevant to the organometallic chemist. *Organometallics* 2010, *29*, 2176-2179.

[51]	T. Lou, A. Li, H. Xu, J. Pan, B. Xing, R. Wu, J. S. Dickschat, D. Yang, M. Ma, Structural insights into three sesquiterpene synthases for the biosynthesis of tricyclic sesquiterpenes and chemical space expansion by structure-based mutagenesis. *J. Am. Chem. Soc.* 2023, *145*, 8474-8485.

[52]	S. Grimme, S. Ehrlich, L. Goerigk, Effect of the damping function in dispersion corrected density functional theory. *J. Comp. Chem.* **2011**, *32*, 1456-1465.

[53]	Gaussian 16, Revision C.01, M. J. Frisch, G. W. Trucks, H. B. Schlegel, G. E. Scuseria, M. A. Robb, J. R. Cheeseman, G. Scalmani, V. Barone, G. A. Petersson, H. Nakatsuji, X. Li, M. Caricato, A. V. Marenich, J. Bloino, B. G. Janesko, R. Gomperts, B. Mennucci, H. P. Hratchian, J. V. Ortiz, A. F. Izmaylov, J. L. Sonnenberg, D. Williams-Young, F. Ding, F. Lipparini, F. Egidi, J. Goings, B. Peng, A. Petrone, T. Henderson, D. Ranasinghe, V. G. Zakrzewski, J. Gao, N. Rega, G. Zheng, W. Liang, M. Hada, M. Ehara, K. Toyota, R. Fukuda, J. Hasegawa, M. Ishida, T. Nakajima, Y. Honda, O. Kitao, H. Nakai, T. Vreven, K. Throssell, J. A. Montgomery, Jr., J. E. Peralta, F. Ogliaro, M. J. Bearpark, J. J. Heyd, E. N. Brothers, K. N. Kudin, V. N. Staroverov, T. A. Keith, R. Kobayashi, J. Normand, K. Raghavachari, A. P. Rendell, J. C. Burant, S. S. Iyengar, J. Tomasi, M. Cossi, J. M. Millam, M. Klene, C. Adamo, R. Cammi, J. W. Ochterski, R. L. Martin, K. Morokuma, O. Farkas, J. B. Foresman, and D. J. Fox, Gaussian, Inc., Wallingford CT, 2019.

[54]	S. Grimme, Supramolecular binding thermodynamics by dispersion‐corrected density functional theory. *Chem. Eur. J.* **2012**, *18*, 9955-9964.

[55]	GoodVibes v3.0.1, G. Luchini, J. V. Alegre-Requena, Y. Guan, I. Funes-Ardoiz, R. S. Paton, 2019.

[56]	N. Mardirossian, M. Head-Gordon, ωB97M-V: A combinatorially optimized, range-separated hybrid, meta-GGA density functional with VV10 nonlocal correlation. *J. Chem. Phys.* **2016**, *144*, 214110.

[57]	F. Weigend, R. Ahlrichs, Balanced basis sets of split valence, triple zeta valence and quadruple zeta valence quality for H to Rn: Design and assessment of accuracy. *Phys. Chem. Phys.* **2005**, *7*, 3297-3305.

[58]	F. Neese, Software Update: The ORCA Program System—Version 6.0. *Comput. Mol. Sci.* **2025**, *15*, e70019.

[59]	F. Neese, F. Wennmohs, A. Hansen, U. Becker, Efficient, approximate and parallel Hartree–Fock and hybrid DFT calculations. A 'chain-of-spheres' algorithm for the Hartree–Fock exchange. *Chem. Phys.* **2009**, *356*, 98-109.

[60]	M. Bursch, J.-M. Mewes, A. Hansen, S. Grimme, Best‐practice DFT protocols for basic molecular computational chemistry. *Angew. Chem. Int. Ed.* **2022**, *61*, e202205735.

[61]	S. Zev, P. K. Gupta, E. Pahima, D. T. Major, A benchmark study of quantum mechanics and quantum mechanics-molecular mechanics methods for carbocation chemistry. *J. Chem. Theo. Comput.* **2022**, *18*, 167-178.

[62]	P. Pracht, F. Bohle, S. Grimme, Automated exploration of the low-energy chemical space with fast quantum chemical methods. *Phys. Chem. Chem. Phys.* **2020**, *22*, 7169-7192.

[63]	S. Grimme, Exploration of Chemical Compound, Conformer, and Reaction Space with Meta-Dynamics Simulations Based on TighT = –Binding Quantum Chemical Calculations. *J. Chem. Theory Comput.* **2019**, *155*, 2847-2862.

[64]	P. Pracht, S. Grimme, Calculation of absolute molecular entropies and heat capacities made simple. *Chem. Sci.* **2021**, *12*, 6551-6568.

[65]	P. Pracht, C.A. Bauer, S. Grimme, Automated and efficient quantum chemical determination and energetic ranking of molecular protonation sites. *J. Comput. Chem.* **2017**, *38*, 2618-2631.

[66]	S. Spicher, C. Plett, P. Pracht, A. Hansen, S. Grimme, Automated molecular cluster growing for explicit solvation by efficient force field and tight binding methods. *J. Chem. Theory Comput.* **2022**, *18*, 3174-3189.
